# Rapid emergence of a maths gender gap in first grade

**DOI:** 10.1038/s41586-025-09126-4

**Published:** 2025-06-11

**Authors:** P. Martinot, B. Colnet, T. Breda, J. Sultan, L. Touitou, P. Huguet, E. Spelke, G. Dehaene-Lambertz, P. Bressoux, S. Dehaene

**Affiliations:** 1 University Paris Cité, Paris, France; 2 NeuroSpin Center, Cognitive Neuroimaging Unit, CEA, INSERM, Université Paris-Saclay, Gif/Yvette, France; 3 Soda Project Team, Premedical Project Team, INRIA Paris-Saclay, Palaiseau, France; 4 Paris School of Economics, CNRS, ENS-PSL, Paris, France; 5 Institute for Public Policies IPP, Paris, France; 6 LAPSCO, University of Clermont Auvergne and CNRS, Clermont-Ferrand, France; 7 Department of Psychology, Harvard University, Cambridge, MA, USA; 8 The Center for Brains, Minds and Machines, Cambridge, MA, USA; 9 LaRAC, University Grenoble Alpes, Grenoble, France; 10 Collège de France, Université Paris-Sciences-Lettres (PSL), Paris, France

## Abstract

Preventing gender disparities in mathematics is a worldwide preoccupation^1,2^. In infancy and early childhood, boys and girls exhibit similar core knowledge of number and space^3–8^. Gender disparities in maths are, therefore, thought to primarily reflect an internalization of the sociocultural stereotype that ‘girls are bad at maths’. However, where, when and how widely this stereotype becomes entrenched remains uncertain. Here, we report the results of a 4-year longitudinal assessment of language and mathematical performance of all French first and second graders (2,653,082 children). Boys and girls exhibited very similar maths scores upon school entry, but a gender gap in favour of boys became highly significant after 4 months of schooling and reached an effect size of about 0.20 after 1 year. These findings were repeated each year and varied only slightly across family, class or school type and socio-economic level. Although schooling correlated with age, exploiting the near-orthogonal variations indicated that the gender gap increased with schooling rather than with age. These findings point to the first year of school as the time and place where a maths gender gap emerges in favour of boys, thus helping focus the search for solutions and interventions.

Why are women under-represented in science, technology, engineering and mathematics domains^1,2^? Biologically, all humans start in life with a core knowledge of objects, space and number that serves as the foundation for mathematical development^8–10^. Number sense, the ability to distinguish sets of objects based on their numerosity, is identical in male and female infants^5^. In young children, most maths-related cognitive tasks exhibit near-zero gender differences in overall performance, and distributions of interindividual variability overlap massively across both genders^3,4,7,8^. Notably, a male advantage for mental rotation and spatial navigation skills is occasionally reported in infancy^7,11^, but it is small, disputed and does not consistently appear before age five^6,12^. Most importantly, gender disparities in maths vary across cultures and testing conditions^7,13,14^. For instance, gender differences favouring males in mental rotation and maths diminish when time pressure or competition are removed, both characteristics frequently associated with maths assessments^14–17^.

For such reasons, young children’s mathematical attitudes, perceptions, interests and competence are thought to constitute universally shared ‘neurocognitive start-up tools’^18^ that are later shaped by a sociocultural belief that girls exhibit lesser proficiency in mathematics relative to boys^5,19–21^. Previous research by economists, sociologists, educational researchers and psychologists has demonstrated that a maths gender gap favouring boys emerges within the first years of schooling in the USA, even when regressing out the effect of age^21–24^. This finding was confirmed by a cohort study following 2,633 children in France, which revealed that the maths gender gap is absent in kindergarten and becomes favourable to boys at age 7–8 (ref. 25). In the USA, the boy advantage appeared earlier among high-achieving students before extending across the entire distribution^21,22^, but this trend varied significantly among ethnic minorities^24^. Both the size and the direction of the maths gender gap, as well as attitudinal variables such as confidence in mathematics, valuing mathematics and maths anxiety, can change rapidly with certain affirmative interventions^2,20,26,27^.

Previous research has explored several potential factors that either exacerbate or mitigate the maths gender gap. Adults’ beliefs and stereotypes, including teachers’ techniques and ratings, could interfere with the neutral estimation of students’ performance and reinforce gender disparities in maths achievement, in both elementary and secondary school^21,28–32^. For example, teachers commonly underestimate girls’ mathematical abilities, assuming that boys possess innate talents whereas girls progress only through diligence and effort, assumptions that may undermine girls’ confidence in their capacity to learn mathematics^21,30,31,33–35^. Girls also suffer more from maths anxiety than boys, especially in scenarios involving competitive or time-limited maths tests, an effect discernible as early as second grade worldwide^36^. Furthermore, maths anxiety in female teachers decreases girls’ mathematical performance, whereas boys remain unaffected^36,37^. Parents and teachers can also be biased in the time spent challenging children of either gender in maths or reading^30,32,38^.

A limitation of former studies, however, is that many were conducted on population subsamples collected 12 to 26 years ago, which, hence, limits their ability to reveal whether the early gender gap stems from pre-existing gender differences, schooling itself, a slow accumulation of sociocultural stereotypes or a combination of these factors^21,25,33,39^.

Here, we shed light on these issues by using an exceptionally large longitudinal dataset (*n* = 2,653,082 children, each of whom completed 46 cognitive tests) comprising the complete population of French typical-aged first and second graders over four consecutive years, which includes the period of school deprivation due to the Covid-19 lockdown. Compared to works in the literature based on survey data, these population-level data allowed us to investigate several potential mediators and to provide extensive heterogeneity analyses, which, in turn, helped refine our general understanding of the possible roots and non-roots of the gender gap in maths.

## A systematic national test battery

**Fig. 1:**
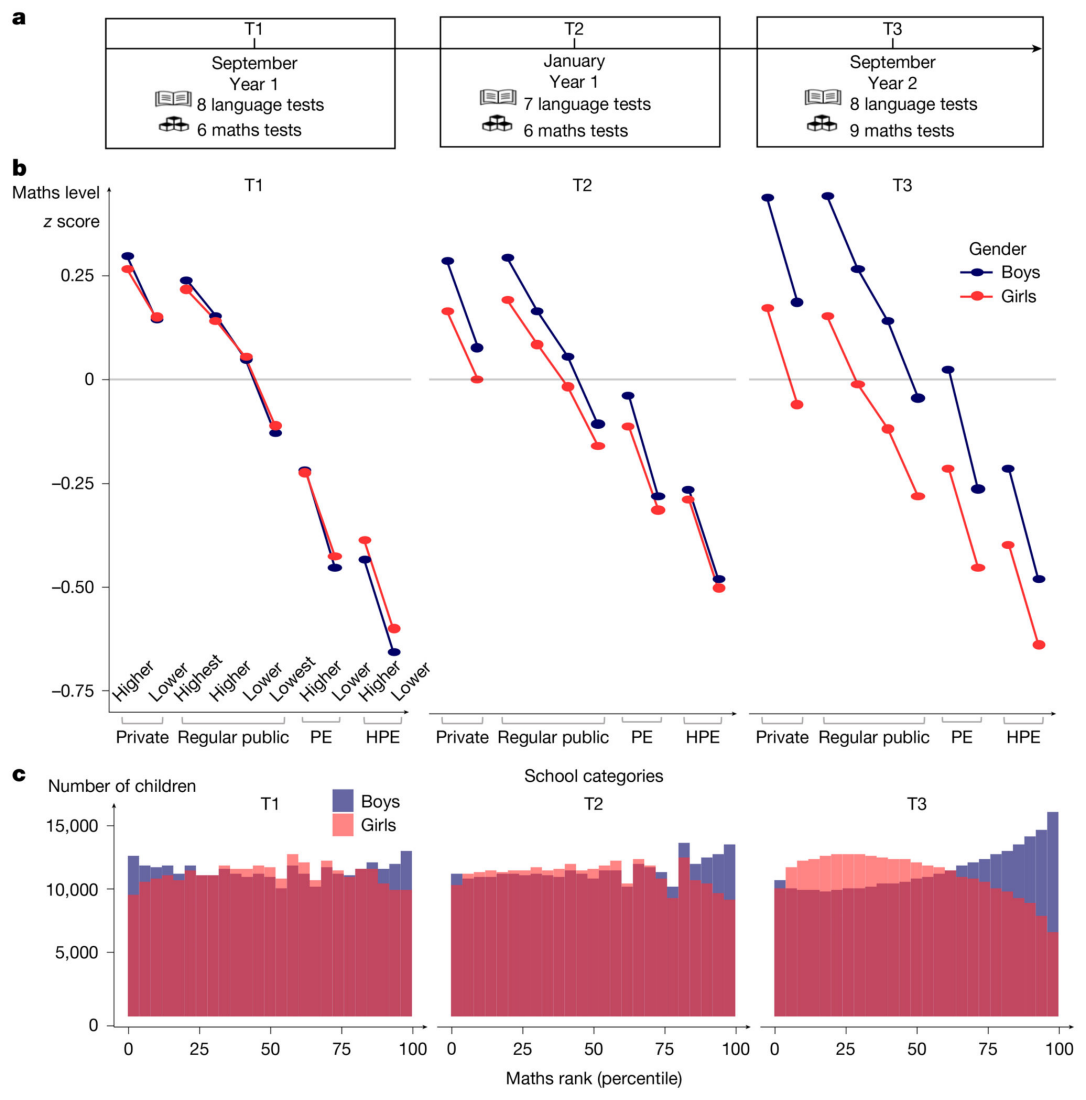
Rapid emergence of the maths gender gap in the French national evaluation programme EvalAide. **a**, EvalAide is a nationwide longitudinal assessment of language and mathematical abilities among all French first and second graders, comprising three measurement periods (T1, T2 and T3). We report EvalAide data from four consecutive years (2018, 2019, 2020 and 2021), for a total of 2,653,082 children. The data are for the 2018 cohort. See extended data figures for a full replication in subsequent years. **b**, Overall performance of boys and girls in mathematics (*z* scores), separately for each of four school categories (private schools, regular public schools, priority education public schools and higher-priority education public schools) and further split as a function of SES (median split or quarter-split for regular public schools). **c**, Distribution of national percentile ranks in maths among boys and girls, showing an initially higher density of boys for both high and low performers, which quickly shifts to a large advantage in favour of boys. HPE, higher-priority education; PE, priority education.

The countrywide French national evaluation programme EvalAide (*évaluer pour mieux aider*: assessing to better help) consists of a battery of language and maths tests that were designed by scientists and educators to provide French teachers with a detailed picture of the needs, achievements and progress of each child in their classroom, thus supporting focused pedagogical interventions and the setting of national standards (Fig. 1a). Every year since 2018, all French children have simultaneously completed tests at the beginning of first grade (T1), after 4 months of school (T2) and 12 months later at the beginning of second grade (T3). The maths tests included digit identification, counting, number comparison, number line knowledge, problem-solving, calculation and geometry, whereas the language tests covered letter knowledge, letter–sound correspondences, phonological awareness, reading aloud, vocabulary, oral comprehension and reading comprehension (Supplementary Table 4). We analysed four consecutive cohorts of 5-to-7-year-old first graders from 2018 to 2022. With such exhaustive longitudinal data, we could preclude sampling bias, and we achieved high statistical power even when analysing subgroups (for example, all children born in the same month and year as there were approximately 50,000 children per subgroup). For simplicity, in the main text we focus on the 2018 cohort, but replications of all figures and analyses are presented in the Extended Data and Supplementary Information.

**Extended Data Fig. 1:**
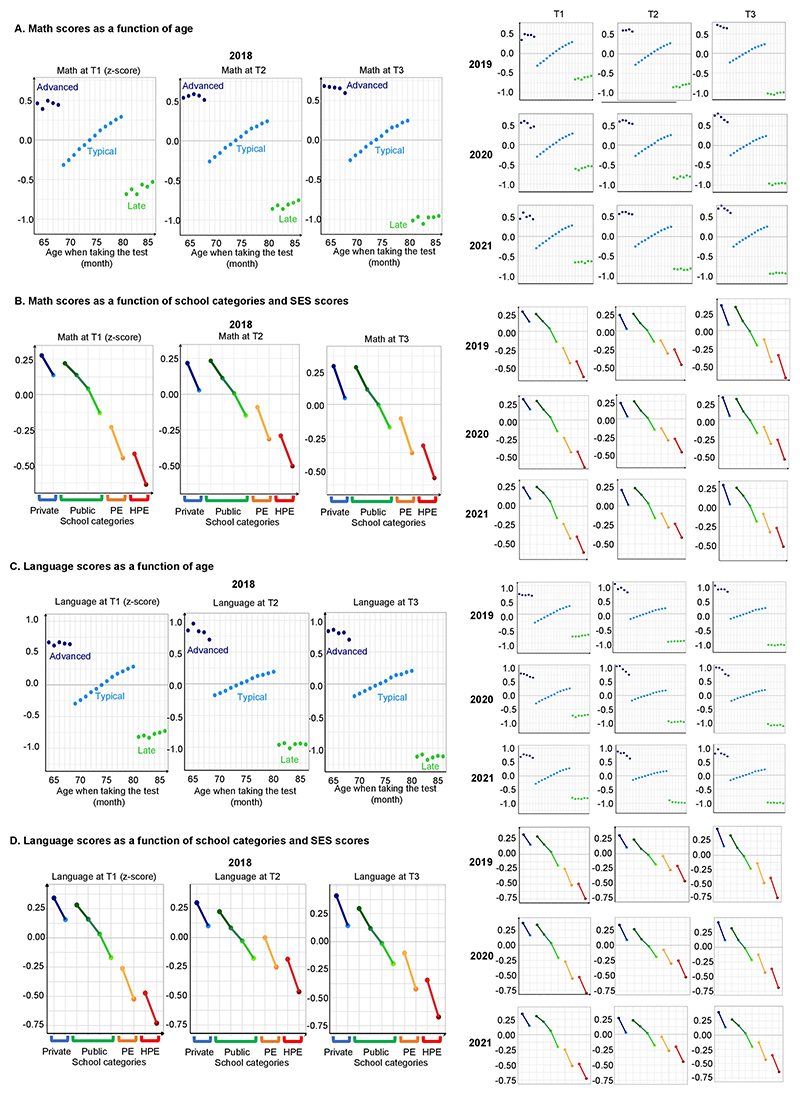
Math and language scores in the years 2018, 2019, 2020, and 2021 (n = 2,871,080 children, including advanced- and late-in-age first and second graders). **A)** Math scores as a function of age in 2018 (main figure), 2019, 2020 and 2021. Mean math levels (in z-score) are presented within the national population, in function of age in month, at T1, T2 and T3. For children within the typical age range (light blue), the yearly data was precise enough to detect a strictly monotonic effect of age in months at each period. For advanced-in-age children (dark blue) or late-in-age (green), a large and increasing learning gap is detected. Bars, indicating one standard error, are often too small to be visible. (**B**) **Math scores as a function of school categories and SES scores in 2018 (main figure), 2019, 2020 and 2021**. Mean math levels (in z-score) are presented within the national population, in function of 10 school subcategories, defined as follow: for each of four main school categories (i.e., private, regular, priority education [PE], and higher-priority education [HPE] public schools), a median or a quartile split (for regular public schools only) was implemented based on the subgroup average socio-economic status (SES). The highest SES score stands on the left and the lowest SES score on the right of the x-axis, for a total of 10 school subcategories: 2 median-split for private schools (in blue), 4 quarter-split for regular public schools (in green), and 2 median-splits for PE (in orange) and for HPE public (in red) schools. Disparities at the start of 1^st^ grade remained present at subsequent time points, apart from PE and HPE schools whose gap decreased during schooling (T2) and increased again after the summer break (T3). **C**) **Language scores as a function of age in 2018 (main figure), 2019, 2020 and 2021. D**) **Language scores as a function of school categories and SES scores in 2018 (main figure), 2019, 2020 and 2021**.

Data quality was high, and a reproducible data-management pipeline was implemented for the few missing values and outliers (approximately 1.2% of all data; no bias was induced by these methods; Supplementary Table 5). Because test difficulty increased with grade level, in accord with the school curriculum, raw scores could not be directly compared across sessions, and therefore, we report normalized and Gaussianized results (*z* scores). The resulting scores were stable, sensitive and relevant. For instance, we easily detected a large and strictly monotonic effect for each additional month of age for both maths and language performance, which reached an effect size of approximately 0.5 standard deviations (s.d.) for children born 11 months apart. We also found a large and increasing lead (+0.5 s.d. from the typical-age mean) for children who were 1 year ahead (1 year younger than the mean age) and an equally large and increasing lag (−0.75 s.d. from the typical-age mean) for those who were 1 year behind (1 year older than the mean age; Extended Data Fig. 1). As expected, the socio-economic status (SES) of a school’s population had a large impact (Extended Data Fig. 1): children in low-income school districts initially lagged behind those in other districts (−0.70 s.d. at T1), but they caught up to some extent over the course of first grade (−0.47 s.d. at T2), in part due to a nationwide policy that halved classroom sizes in these districts. These effects were stable across all years (2018, 2019, 2020 and 2021).

## Rapid emergence of a maths gender gap

**Extended Data Fig. 2:**
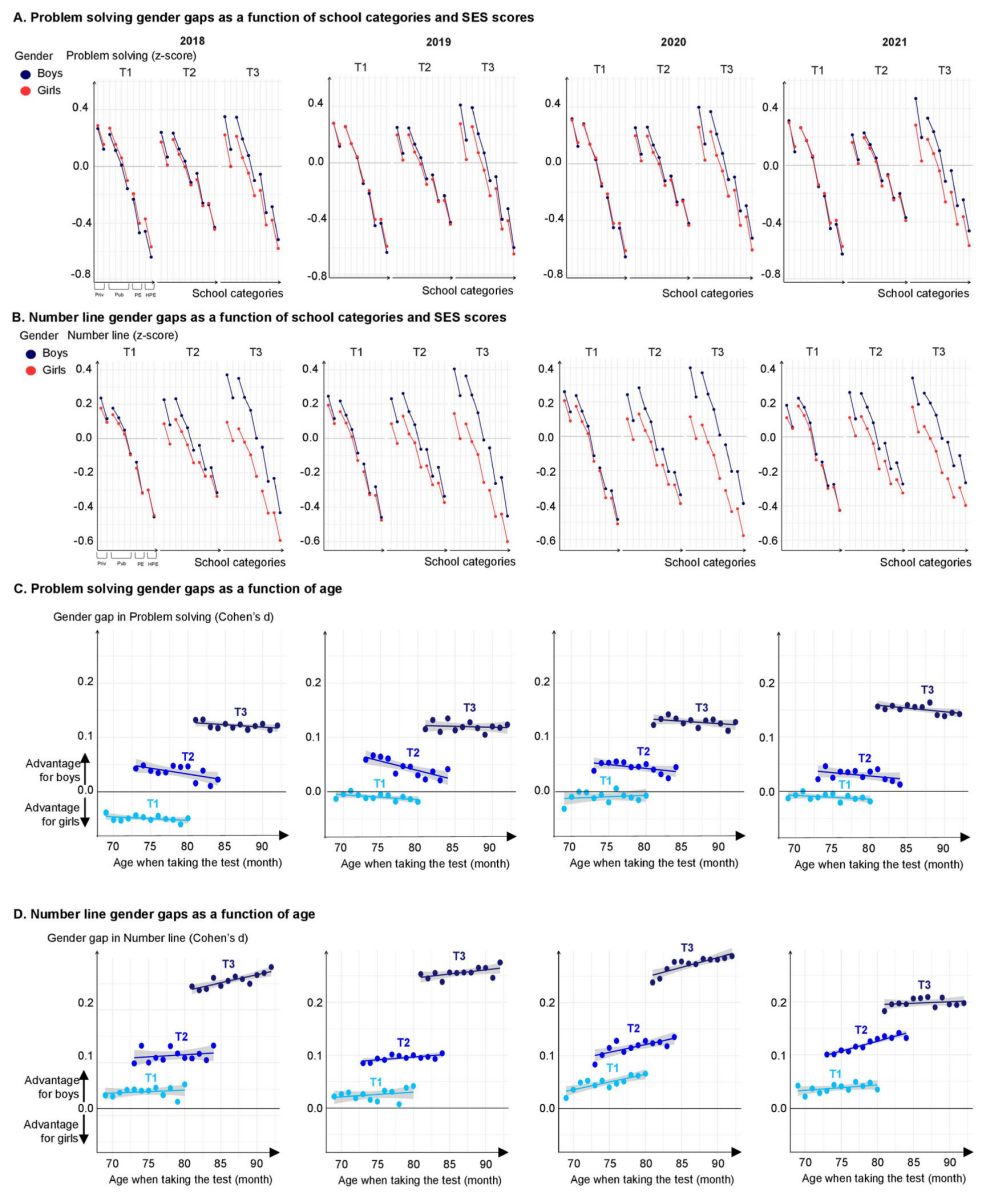
Gender gaps in problem solving and number line subtests in the 2018, 2019, 2020 and 2021 cohorts. Performance of boys (blue) and girls (red) in z-score in (**A**) problem-solving and (**B**) number-line assessments. Within each school category, the gender gap was almost null or small at school start (T1), detectable after 4 months (T2), and large after one year of schooling (T3), except for higher SES score school categories, where the gender gap was already in favor of boys. Gender gaps effects on (**C**) problem-solving and (**D**) number line, as measured by Cohen’s d in function of age. Bars, indicating one standard error, are too small to be visible (n = 2,653,082 children).

**Extended Data Fig. 3:**
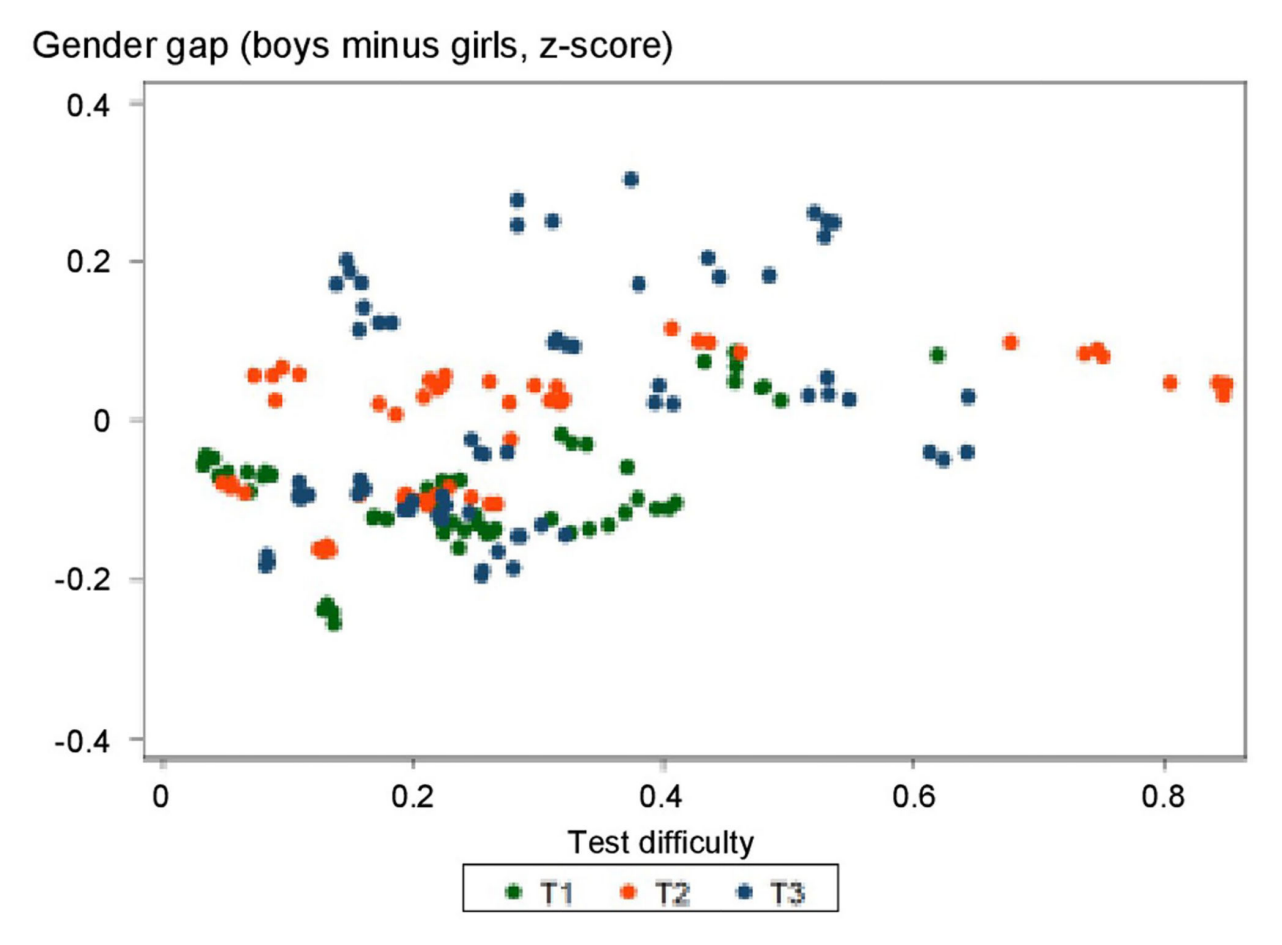
Variation in the gender gap across mathematical subtests, plotted as function of subtest difficulty and subtest period. Data from 20 subtests (i.e., calculus, addition, subtraction, writing numbers, reading numbers, comparing quantities, number line and problem solving) at T1, T2 or T3 and from the four consecutive cohorts. Math gender gaps could not be attributed solely to an increase in test difficulty as the average difference in gender gap within a given type of subtest between T1 and T3 moves from 0.194 SD without the test difficulty variable to 0.179 SD when controlling for test difficulty (see SI Table S7).

**Fig. 2:**
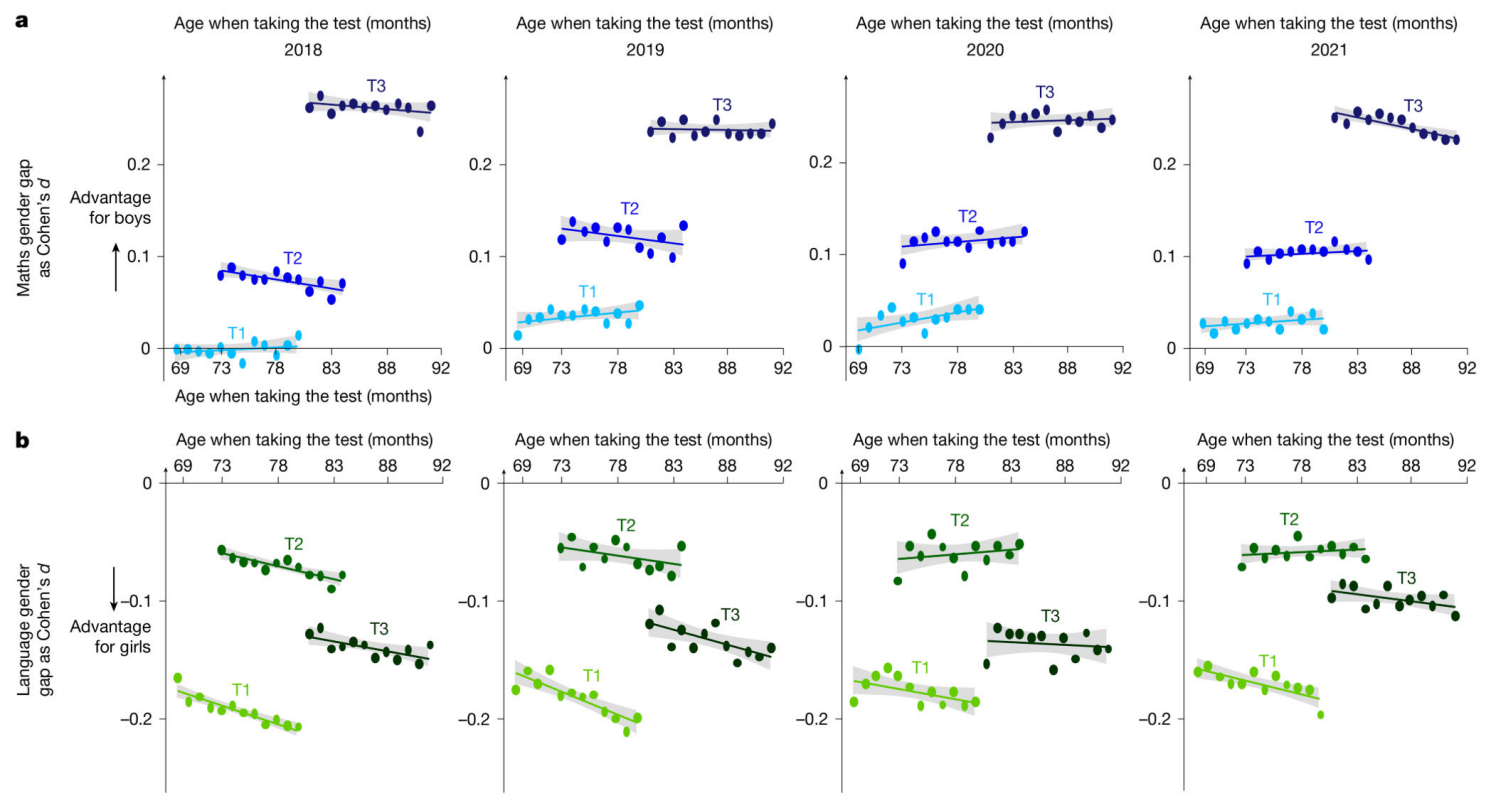
Decorrelation of age and schooling. **a**, Size of the gender gap at T1, T2 and T3 as a function of the age of the children in months when taking the test, separately for the four cohort years (Cohen’s *d*). Because a strict cutoff on birthdate determines school entry, children of the same age (*x* axis) can differ in their level of schooling (colours and regression lines). Regardless of age when taking the test, children showed minimal or no maths gender gap at T1, but there was a growing effect after 4 months (T2) and 12 months (T3) of schooling. Bars, indicating one standard error, are too small to be visible. **b**, Same format for the language gender gap, which exhibits distinct dynamics: girls are already ahead of boys at T1, an effect that widens with age. It is transiently reduced during the school year (T2) and partially returns after the summertime school break (T3).

**Fig. 3:**
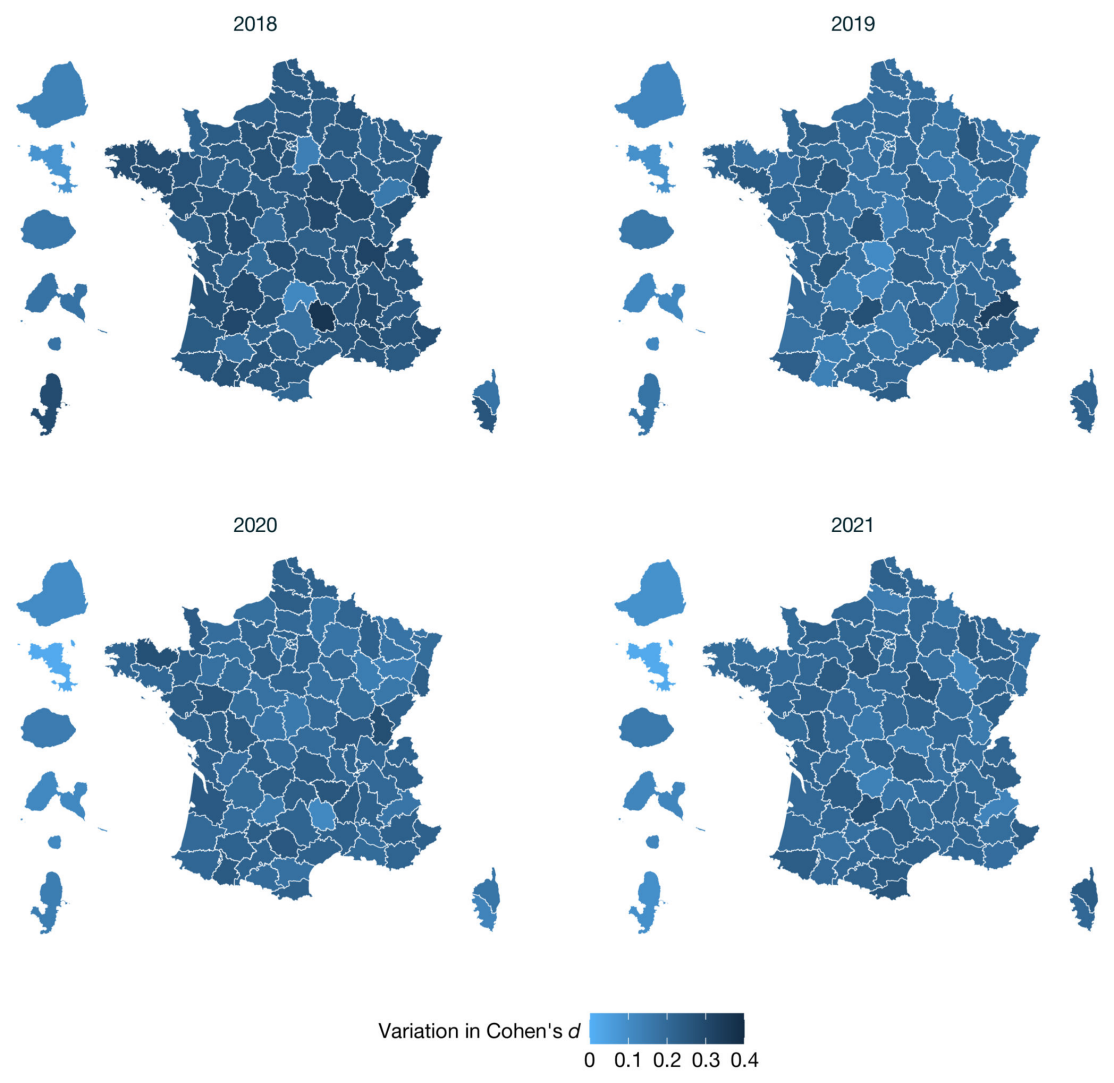
Evolution of the maths gender gap (Cohen’s *d*) between T1 and T3 in each French geographic *département* in 2018, 2019, 2020 and 2021 (*n* = 2,653,082 children). A *département* is an administrative division of the French territory. There are 101 *départements* in France. Overseas *départements* (not to scale) are placed to the left of mainland France. For each *département*, the variation of Cohen’s *d* between T1 and T3 was measured. Maps were created using OpenStreetMap with the software R v.4.3.2 2 under a creative commons licence CC BY-SA 2.0. Data on French department maps were available open source.

**Fig. 4:**
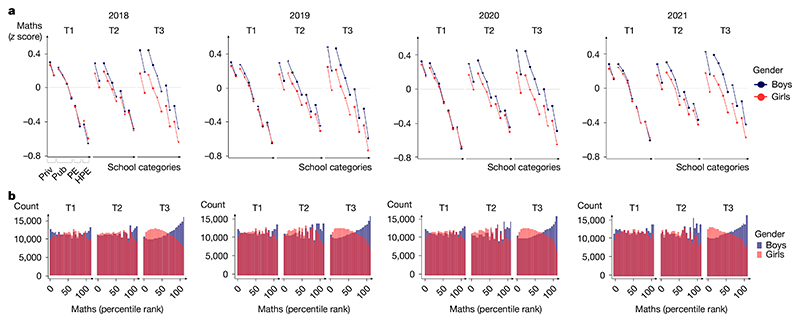
Rapid emergence of the maths gender gap in the 2018, 2019, 2020 and 2021 cohorts (*n* = 2,653,082 children). **a**, Maths gender gaps as a function of school categories and SES scores (*z* score) (same format as Fig. 1b). **b**, Distribution of percentile ranks for boys and girls (same format as Fig. 1c).

The data also revealed the rapid emergence of a maths gender gap as children progressed through school (Fig. 1b), as found in previous studies^21–24^. At school entry, the average mathematical performance of boys and girls was nearly identical in 2018 (Cohen’s *d*_T1_ = −0.0166, where the negative sign indicates a very small advantage for girls). After 4 months of schooling, a small but already highly significant gap was found favouring boys (Cohen’s *d*_T2_ = 0.0468), and its effect size quadrupled by the beginning of second grade (Cohen’s *d*_T3_ = 0.2230) (Table 1). The significance of that change across time was confirmed by a statistical growth model (Supplementary Table 6). The rapid emergence of the gender gap was replicated in every cohort across four consecutive years (Table 1), and thus, it was not due to idiosyncratic societal, economic or curricular changes in a particular year. Indeed, it was also seen when comparing first- versus second-grade data acquired simultaneously within the same weeks (for example, when comparing the T3 data point for the 2018 cohort to the T1 data point for the 2019 cohort in Fig. 2a for data acquired in September 2019). The maths gender gap was found within each region of France (Fig. 3), in schools serving communities at both high and low socio-economical levels (Extended Data Fig. 1 and Fig. 4a), and in most tests (Table 2). Although the tests were not identical at T1, T2 and T3, as they were adapted to the children’s progress through the school year, a similarly rapid emergence of the gender gap was found when we restricted the analysis to the problem-solving and number line subtests, two tests that were repeatedly probed at T1, T2 and T3 with only small variations in item type (Table 1 and Extended Data Fig. 2). Furthermore, although test difficulty was correlated with the size of the gender gap, the emergence of the maths gender gap between T1 and T2 or T3 could not be attributed solely to an increase in test difficulty. For example, the average difference in gender gap within a given type of subtest between T1 and T3 moved from 0.194 s.d. without the test difficulty variable to 0.179 s.d. when controlling for test difficulty (Supplementary Table 7 and Extended Data Fig. 3).

**Extended Data Fig. 4:**
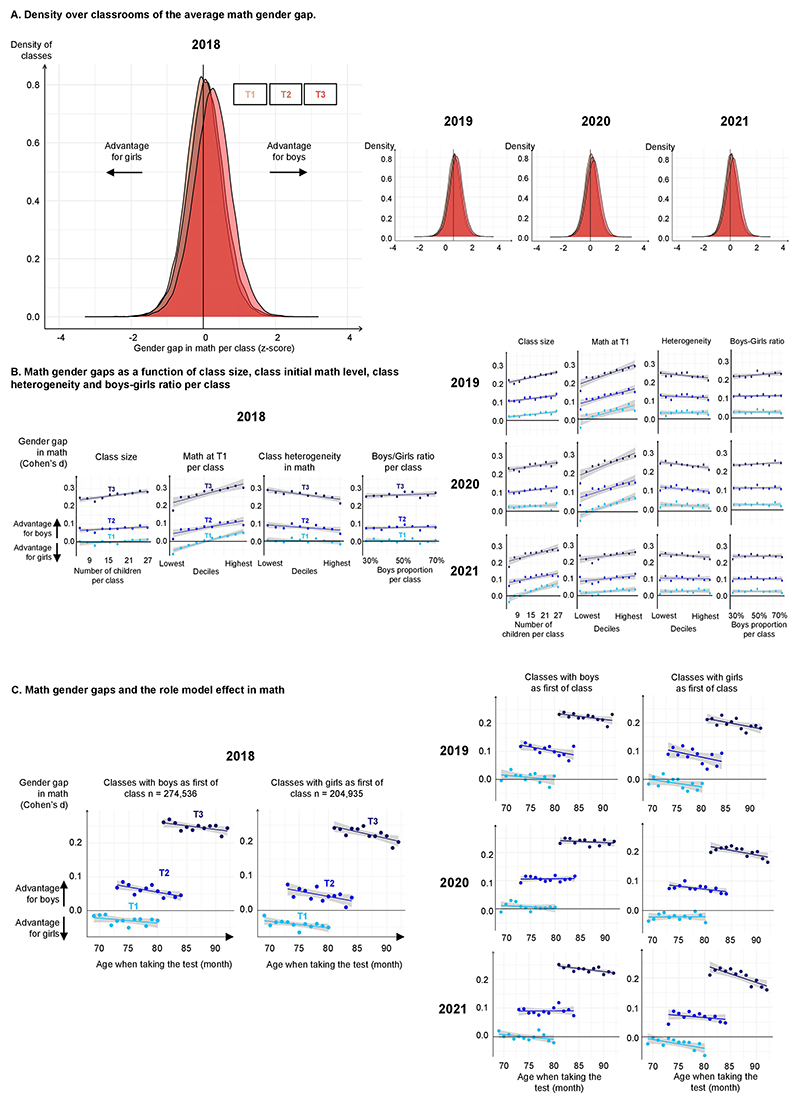
Class effects on the math gender gap. (**A**) **Density over classrooms of the average math gender gap**, expressed as the difference in average z score between boys and girls (for classes with at least 30% of boys and 30% of girls, n = 2,455,483). The distribution was centered on zero at T1, but a majority of classrooms showed a gender gap at T2 and especially at T3. Results were similar for 2018, 2019, 2020 and 2021. (**B**) **Math gender gap as a function of class size, class initial level in math, class heterogeneity of level in math, and boys-girls ratio per class (in Cohen’s d)**. A higher heterogeneity of level in math was associated with a lower gender gap in favor of boys whereas a larger class size (in 2018 and 2019) and higher-class level in math were associated with a higher gender gap in favor of boys. Boy/girl ratio per class did not have much effect on gender gaps in math. Bars, indicating one standard error, are too small to be visible. (**C**) **Math gender gap and the role model effect in math (in Cohen’s d)**. Having a girl or a boy as the first of class in math at T1 had a small effect on the gender gap, measured in Cohen’s d. For this analysis, data from the best student(s) at T1 in math were excluded. Classes with girls as first of class in math developed a slightly smaller gender gap in favor of boys.

An examination of the distribution of maths scores over children clarified how the gender gap emerged (Fig. 1c). At school onset (T1), although boys and girls had the same mean, boys were over-represented at both ends of the distribution (lowest and highest deciles), as has been previously described^5,21,22^. After 1 year of schooling, however, the distribution had shifted massively, with the top 5% of children in maths at T3 comprising more than twice as many boys as girls (2.33 boys for each girl among the top 5% at T3; Fig. 1c). Similar results were found when the gap was computed within each class as the *z* score difference in maths performance between boys and girls (Extended Data Fig. 4a).

## Universality of the maths gender gap

**Extended Data Fig. 5:**
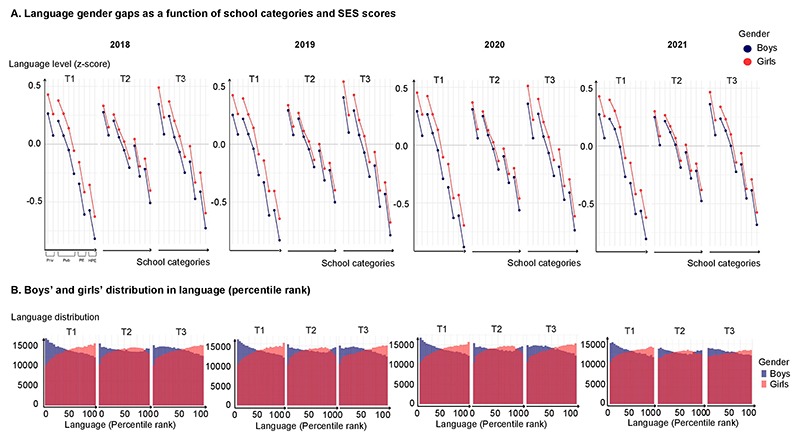
Gender gaps in language in the 2018, 2019, 2020 and 2021 cohorts (n = 2,653,082 children). Girls were already ahead of boys at T1 (in z score), an effect that widened with age and was only transiently reduced during the school year (T2), but largely restored after the school break (T3). Results were replicated in 2018, 2019, 2020 and 2021. No significant variations in language gender gap were noted when comparing Covid years to 2018 and 2021.

**Extended Data Fig. 6:**
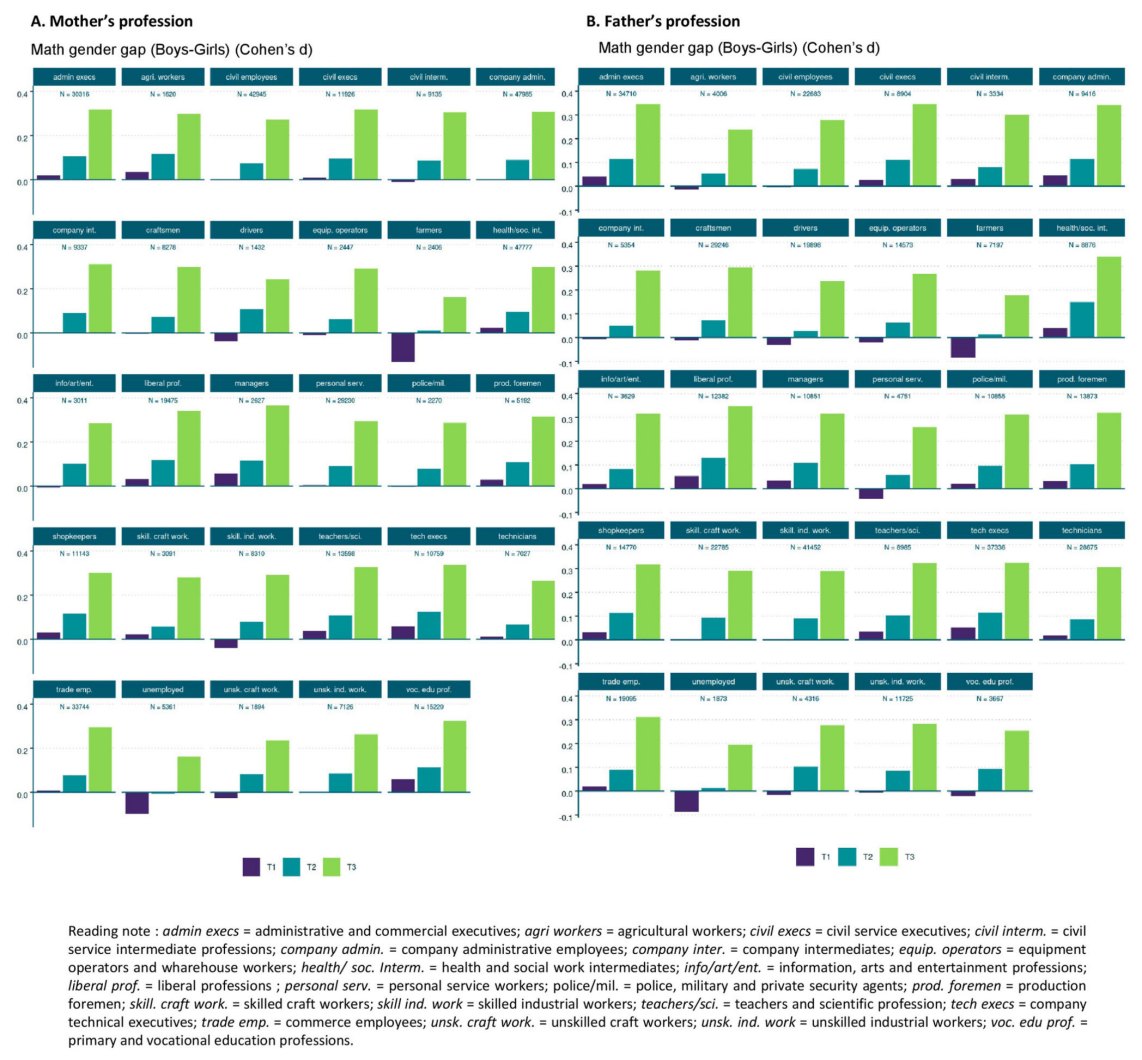
Math gender gaps as a function of the parents’ profession in the 2018–2019 cohort (n = 569,771). Math gender gaps, measured as Cohen’s d, were analyzed as a function of (**A**) the mother’s profession and (**B**) the father’s profession. Such data was only available for the 2018–2019 cohort, as they were registered upon entering 6th grade in 2023. A widening math gender gap was observed in children, regardless of their parents’ professions. Figure adapted with permission from ref. 51, HAL.

**Fig. 5:**
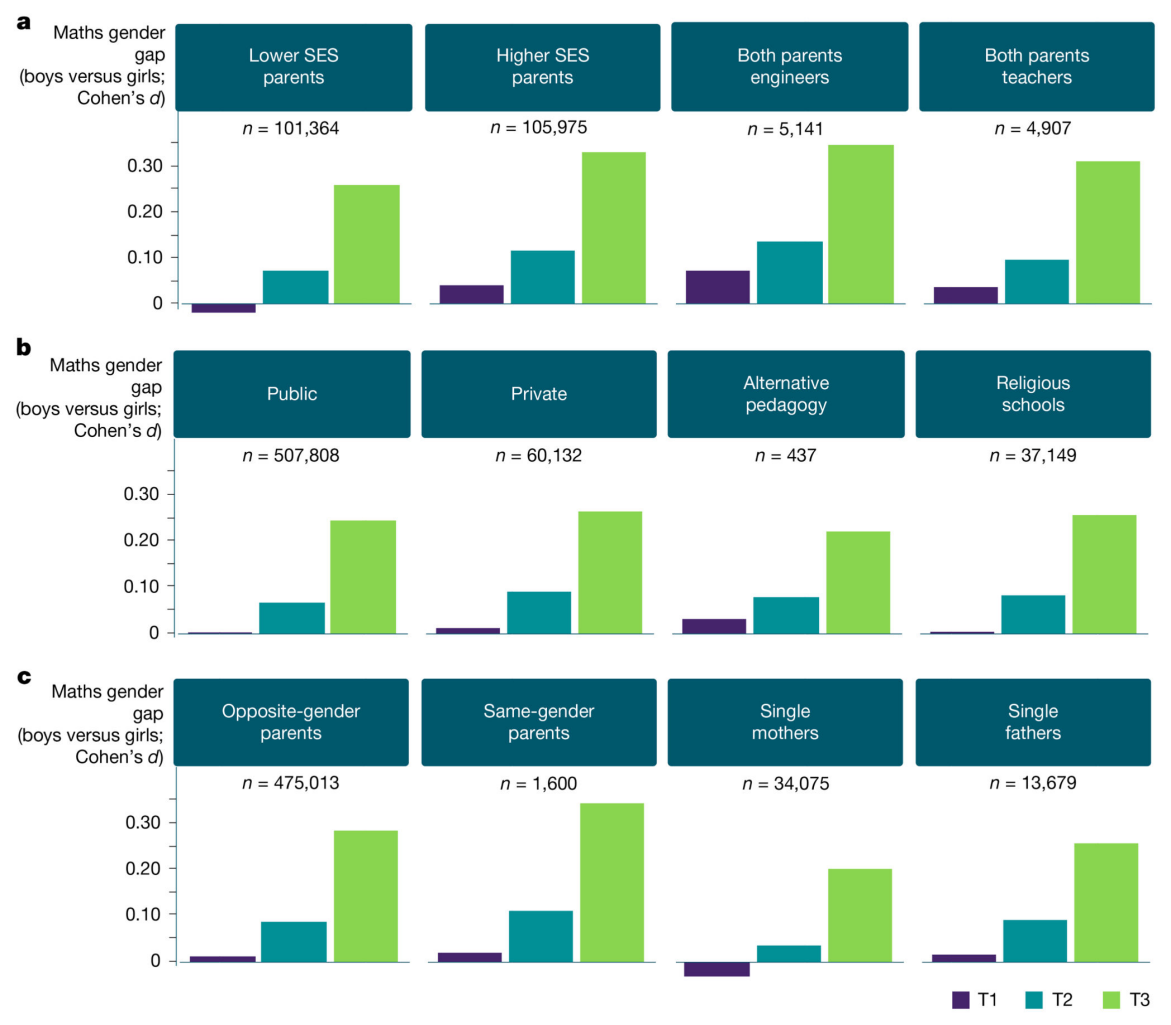
Evolution of the maths gender gap from T1 to T2 and T3 in targeted population subgroups. **a**–**c**, This analysis focuses on familial data exclusively available for the 2018/2019 cohort (*n* = 569,771). An increase in the maths gender gap (Cohen’s d) was evident across all levels of familial SES (**a**), with more pronounced effects observed among higher-income families, notably families where both parents worked in scientific professions (for example, engineers) or were teachers; in various types of schools (**b**), including those employing non-traditional teaching approaches (for example, Freinet or Montessori pedagogy) and religious schools; and in various family types (**c**), with families with same-gender parents exhibiting a larger maths gender gap compared to other family structures (opposite-gender parents, single mothers or single fathers). Figure adapted with permission from ref. 51, HAL.

A quickly growing maths gender gap favouring boys, emerging in less than 4 months, was observed in all types of schools, private or public. However, the gap was larger among higher-SES schools (Figs. 1b, 4a and 5a and Table 3), a phenomenon not found for language (Extended Data Fig. 5). In the 2018/2019 cohort, for which detailed familial information was available, the maths gender gap emerged in schools with non-standard teaching methods (for example, Freinet or Montessori pedagogy) and in religious schools (Fig. 5b). The maths gender gap also emerged at all levels of familial SES (Fig. 5a), independently of the occupation of the parents (Fig. 5a and Extended Data Fig. 6) and regardless of family composition (in families led by opposite-gender parents or same-gender parents, single mothers or single fathers; Fig. 5c). In particular, the gap at T3 was larger among higher-SES families (Cohen’s *d* = 0.32 versus 0.26 in lower-SES families) with larger effects when both parents held scientific occupations (for example, engineers, Cohen’s *d* = 0.35) or were teachers (Cohen’s *d* = 0.31) (Fig. 5a and Extended Data Fig. 6).

## The gap varies with schooling, not age

Although children’s age increased across the three longitudinal testing points, our corpus allowed us to partially disentangle the effects of age and amount of schooling. The French system requires children to enter school in the year of their sixth birthday. As a consequence, at each test time (0, 4 or 12 months following school entry), the children varied in age over a 12-month range. When plotted as a function of age alone within a given testing period, the gender gap remained nearly constant (Fig. 2). Furthermore, some children with the same age in months at the time of testing had different amounts of schooling (vertical comparisons in Fig. 2). Within every such age slice, a significant increase in the gender gap was found between T1 and T2 (0 versus 4 months of schooling) or between T2 and T3 (4 versus 12 months of schooling) (Tables 1 and 3). Also, because of the January cutoff on school entry, when children born in December of a given year take the T3 test after having been enrolled in school for a whole year, the children born in January of the following year are only taking the school-entry T1 test. Even though these children were born only a few days apart and were tested simultaneously, comparing their T1 and T3 scores again revealed a large and significant gender gap in the schooled group, thus differing significantly from the unschooled group (Supplementary Table 8). Although the test contents also varied among the three time points, all the above findings were replicated with data from the number line and problem-solving tasks, which were applied (with new items) at all three time points (Extended Data Fig. 2c,d).

Although there could be selection bias because parents may plan to have to a child in a given month, a control analysis alleviated this potential concern: regarding children’s characteristics such as SES or attending a private or public school, gender gaps were either absent or very small and showed no evidence of differing between December of a given calendar year and January of the following year (Supplementary Table 9). For each birth month, the gender gap in maths always increased strongly from T1 to T2 to T3 but showed little or no consistent difference between children born in December and those born in January the next year (Supplementary Table 9). We confirmed those insights with nine regression discontinuity designs that used the exact date of birth as the running variable (with cutoff dates of 1 January 2013, 2014 or 2015) and scores at T1, T2 or T3 as the outcome variable. Because parents cannot precisely target the date of birth, this approach, which is standard in the literature^40^, allowed us to capture the local effect of being almost 1 year older when taking a given test (and potential factors that are confounded with it, such as the age at school start or the relative age within one’s kindergarten or school cohort) while holding constant the time spent in formal schooling. We first confirmed with these formal regression discontinuity designs that girls’ and boys’ characteristics, such as SES, are balanced on each side of the cutoff dates (Supplementary Table 9). The results show that the effect of delaying school entry was large on test scores (around 0.7 s.d. at T1, 0.6 s.d. at T2 and 0.55 s.d. at T3) but was comparable for girls and boys (for details, see Supplementary Tables 10–13 and Supplementary Figs. 1 and 2). This is consistent with findings in the literature^40^ and confirms, more formally, our main result that the emergence of the gender gap in maths between T1 and T3 cannot be explained because the children are older at T3.

## Variables modulating the maths gender gap

To further determine which variables modulated the gender gap, T3 data were entered into a mixed-effect multilevel linear model with gender and its interactions with several potential modulators: age, SES, class size, T1 performance in language and maths, class heterogeneity in maths, boy-to-girl ratio and gender of the top student in the class (Table 3). The gender gap emerged at all levels of these variables, with only small modulations (Extended Data Fig. 4). Although age had a large positive effect on mean test scores, as previously noted (Extended Data Fig. 1), a negative interaction between age and gender indicated that age actually had a small protective effect on the gender gap: in every cohort, older children displayed a slightly but significantly smaller gender gap in maths at T3 (in 2018, *β*_age × gender_ = −0.0094 (0.0019), *P* < 0.0001; Table 3 and Fig. 2). Gender also interacted with the initial level in language and maths: the gender gap was larger for pupils with a higher initial maths level and a lower initial language level. At the class level, the gender gap tended to be larger at T3 in classes where the first-in-class at T1 was a boy (role model effect) and class size was larger, whereas the gender gap decreased in classes with greater heterogeneity in maths (although the latter effects were not always significant in every year; Table 3 and Extended Data Fig. 4). The gender gap was also larger in schools with a higher average SES (*β*_SES_ _× gender_ = 0.0049 (0.0020), *P* = 0.0146, Table 3).

**Extended Data Fig. 7:**
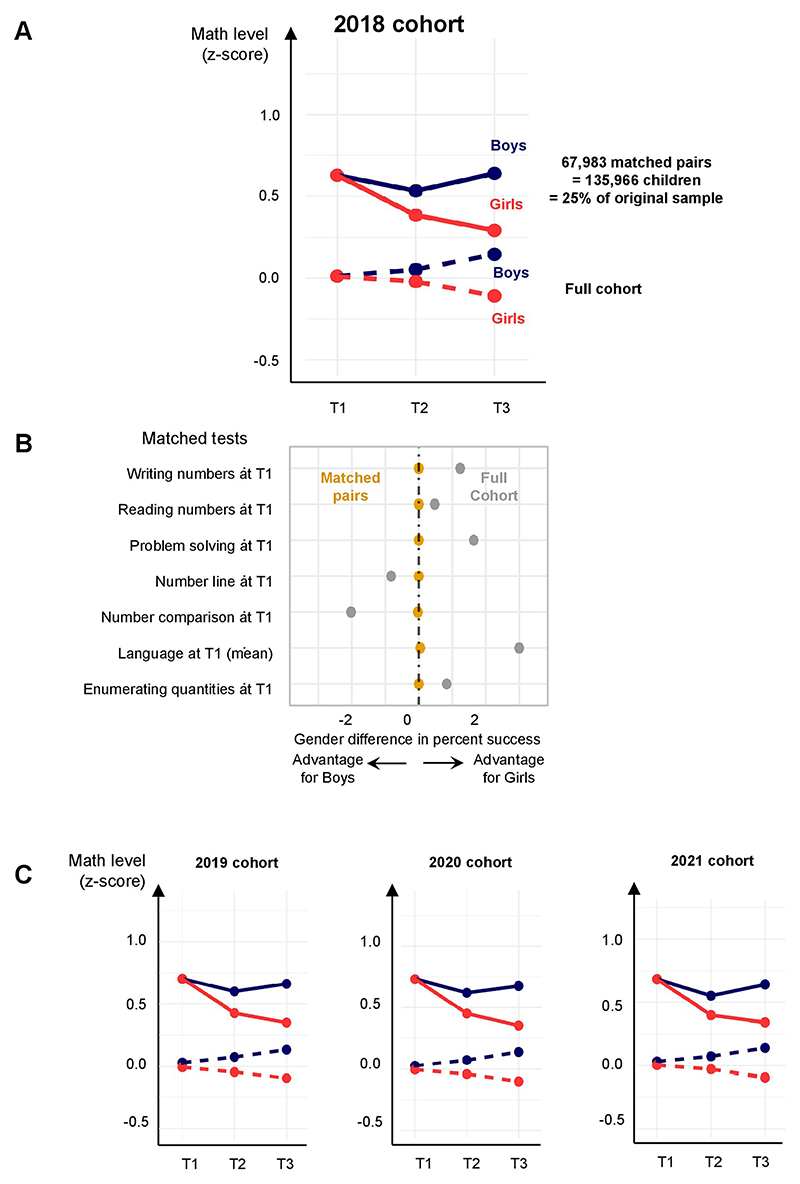
Rapid emergence of the math gender gap in a subsample of boys and girls whose performance at T1 was tightly matched. Boys and girls of typical age (69 to 80 months) were paired on their math scores at T1, SES, age, language at T1 and school category (see SI Table S14). (**A**) Results from the 2018 cohort. Even when T1 differences were eliminated by this matching procedure, a gender gap emerged at T2 and widened at T3, in line with a cumulative influence of school exposure. (**B**) Prior to matching, the gray points indicate that there were small differences between boys and girls, either positive or negative depending on the specific test, which vanished after matching (orange dots). (**C**) Results from the 2019, 2020 and 2021 cohorts, same format as panel A. Note that in the matched pairs, children are no longer representative of the national sample (dotted line). While this difference is notable, it is irrelevant to the main point of this analysis, which is to show that boys and girls can be very similar at T1, and still diverge in math at T2 and T3.

Together, these findings indicate that girls engage more readily in maths learning when they are initially advanced in maths or can identify with the first-in-class, and less so if they are initially more advanced in language and reading. However, pre-existing differences between boys and girls did not explain the maths gender gap at T3, because even when controlling for them, the main effect of gender remained unchanged (Table 3 and Supplementary Table 16, models 3 to 9). Furthermore, even when we selected pairs of boys and girls who were closely matched at T1 on every maths test, mean language performance, SES, age and school category (*n* = 135,966 children or about 25% of a cohort and no longer representative of the population), their results still diverged at T2 and T3 (Extended Data Fig. 7 and Supplementary Table 14). Six different reweighting techniques based on the same variables confirmed this divergence (Extended Data Table 1).

## Language skills follow distinct dynamics

Importantly, language performance followed strikingly different dynamics than maths (Fig. 2 and Extended Data Fig. 5). A gender gap in language, favouring girls, was already sizeable at T1 (Cohen’s *d* = −0.1935), had shrunk at T2 (Cohen’s *d* = −0.0845) and widened again at T3 (Cohen’s *d* = −0.1371) (Table 1). Controlling for differences at T1, the gender gap effect on language at T3 was approximately 10 times smaller than on maths at T3 (*β*_gender gap language__T3_ = −0.0328 (±0.0039) and *β*_gender gap maths__T3_ = 0.3453 (±0.0038); Table 3 and Extended Data Table 2). Thus, for language, an early and sustained female advantage existed (Cohen’s *d* for the gender gap in maths −0.1935), which, unlike maths, was transiently reduced at T2 (Cohen’s *d* = −0.0845) but regained strength at T3 (Cohen’s *d* = −0.1371 in 2018) (Fig. 2 and Table 1). Overall, school appeared more beneficial to boys, who progressed in both maths and language, yet the language gender gap was well established before school and, in the longer term, changed much less with schooling than the maths gender gap.

## Changes across years and the pandemic impact

Although the maths gender gap was notably stable, we briefly comment on two minor variations that were observed across the four consecutive cohorts (Fig. 2). First, between T2 and T3 of the 2019 cohort, the pandemic-induced school disruption deprived French first and second graders of at least 52 school days, which were followed closely by the usual 2.5-month summer vacation. Interestingly, the gender gap in maths grew significantly less during this period compared to other years (T3 − T2 maths gender gap * year 2018 versus 2019 = −0.0685 (0.0027), *P* < 0.0001), and recovered only partially in the 2020 cohort, as some schools closed again (T3 − T2 maths gender gap * year 2019 versus 2020 = 0.0139 (0.0026), *P* < 0.0001 and 2020 versus 2021 = 0.0083 (0.0026), *P* < 0.0001) (Fig. 2a and Extended Data Table 3). It has previously been reported that the maths gender gap decreases during the summer break^41^, and both findings may indicate that school context plays a role in the maths gender gap. No such variations were seen for the language gender gap, confirming that it is driven by other factors (Fig. 2b and Table 1).

A second variation was that, in 2019, 2020 and 2021, but not 2018, a small but significant maths gender gap favouring boys was already present at T1 (Fig. 2), only in maths (Table 1). One possible explanation is that, in May 2019, before T1 acquisition for the 2019 cohort (September 2019), the French education ministry issued a formal request to kindergarten teachers, asking them to prepare children for the coming first-grade national assessments by introducing more formal training in maths and language, thus making kindergarten more like elementary school^42^.

## Summary of empirical observations

In summary, in an exceptionally large and exhaustive dataset from all French first and second graders, we observed a rapid emergence of a gender gap in maths favouring boys in all types of schools after only 4 months of schooling in first grade, irrespective of the children’s age. Before school entry, girls and boys were well matched in their basic numerical abilities, regardless of their age, with only a small excess of boys at both extremes of the scale. After just 4 months of schooling, however, the maths gender gap emerged and deepened as maths instruction proceeded. Our findings support previous studies which, based on smaller samples, found that the maths gender gap arises as early as first or second grade^21–24^. The present analyses conducted on population-level data allowed us to go one step further and show that the maths gender gap is an early and widespread phenomenon covering every stratum of society, regardless of school type and related pedagogy, SES, parental occupation, family composition, school environment and geographical location, all basic variables rarely if ever studied simultaneously in previous work relying on smaller surveys. We were also able to test the relations between the maths gender gap and variables that are seldom integrated, a fortiori simultaneously, at the scale of an entire population, such as the average socio-economic level of schools, mean mathematical performance of the class, class heterogeneity, class gender ratio or gender of the top student in class. The maths gender gap turned out to be only slightly moderated by those variables, confirming its robustness and generality (Table 3).

## Limitations of our data and inferences

Several caveats should be kept in mind. First, the present data are descriptive in nature and, thus, cannot be used to pin down the root causes of the gender gap. Second, the existence of only three discrete measurement points (approximately 0, 4 or 12 months after school entry) prevents any detailed evaluation of the potentially continuous effect of school exposure or the effect of vacations. Third, the tests were not strictly identical at those three time points, as they aimed to track children’s progress during the school year. Fourth, our data come from a single country, France, whose specificities are discussed in the Supplementary Information. Within France, however, the present findings may generalize across age, geography, initial levels, SES and types of classes, and they accord with previous findings of a rapid emergence of gender biases in other countries^21,32,39^.

## Potential causes of the maths gender gap

Given the observational nature of our data, any attempt to infer the mechanisms producing the maths gender gap must remain hypothetical, but some explanations are, nevertheless, more consistent with our findings than others. For instance, the gap is unlikely to be a consequence of girls’ previous comparative advantage in language and reading^43^, which might have occurred if children, perhaps, under parental and teacher encouragement, invested in their own pre-existing strengths. Maths and language abilities at T1 are correlated, respectively, with increases and decreases in the maths gender gap at T3, but a gap continued to emerge even when such differences were regressed out. The lack of average gender differences at the beginning of grade 1 also gives little credibility to the idea that the emerging gender gap is related to fundamental gender differences in aptitudes. Our data do not, however, exclude more complex interactions between nature and nurture, for instance a latent advantage of boys in maths learning that comes into play only at the onset of formal education or of gender differences in competitiveness and test-induced anxiety that are exacerbated by school entry. Girls may exhibit greater maths anxiety, possibly reinforced when facing competitive tests, a behaviour that may explain why, among all maths and language exercises, the male advantage is more pronounced in more challenging, new or complex tests tapping executive functions^36,44^. This explanation is congruent with our finding that greater test difficulty enhances the gender gap. That boys tend to rely more on faster memory-retrieval strategies could also be a factor^45^, although this cognitive mechanism fails to explain the large gender gap on tests such as the number line test, which do not involve arithmetic fact retrieval in the classical sense of the term. We can only speculate as to why the gender gap emerges rapidly upon school entry rather than slowly as a function of age. Primary school may mark the moment in children’s curriculum when maths-related activities or exercises (for example, counting and subtracting) start to be more clearly identified as belonging to the maths domain, with separate school textbooks and teaching hours for maths subjects. This sudden labelling of maths-related activities as ‘maths’ (whereas language activities start earlier in preschool) might give space for gender stereotypes surrounding maths to emerge, to be internalized by children and, eventually, to affect their self-concept and performance. Primary school teacher attitudes may contribute to this dissemination, if teachers interact differently with boys and girls^20,30^, transmit their maths anxiety to girls^37^, encourage girls’ efforts at reading more than at maths^21^, or attribute the successful mathematical performance of boys to their greater intellectual power and the successful mathematical performance of girls to their greater diligence^28^.

The onset of schooling may also prompt a change in the attitudes of parents, family members, other professionals and the children themselves^2,30^. The simple belief that boys and girls have different interests and abilities can reinforce gender disparities, especially as girls show greater facility with language, as shown here and elsewhere^20,30,43^. With schooling, parents may start spending more time on their children’s formal education and, therefore, transmit gender norms, including those related to maths. This extra investment at the onset of schooling may be greater in high-SES families. This would explain why we and others have found that a larger gender gap emerges in high-SES families, schools and countries^19,46^.

## Consequences for interventions

The present findings indicate that interventions should come early in the curriculum. From a policy perspective, tackling the gender gap in mathematics at the earliest stage (kindergarten or first grade) may be most cost-effective, both because maths instruction is highly cumulative and because programmes that start early may reach girls before they lose confidence in their mathematical abilities and become resistant to counter-stereotypic information^47^.

Which factors should be targeted? Although our evidence is only correlational, we found that class-level variables, such as class size, gender ratio, heterogeneity in maths level or gender of the student at the top of class, have only a small modulating influence. Given that our data indicate that the maths gender gap starts with entry into the school system, improving teacher training should undoubtedly be one of the most powerful levers. Encouraging teachers’ gender-fair ratings and active cooperative practices^27,29,35^, such as questioning girls and boys equally often during maths and science instruction and focusing equally on the talents and efforts of children of both genders^7^, are efficient practices that should become part of teachers’ basic training. Boosting teacher training in maths to increase their confidence and interest in this topic could also be effective, especially in countries such as France where most primary school teachers are female^27^. Interventions can also convince boys and girls that maths is worth the effort by exposing children to both male and female role models with whom they can identify^48^; providing girls with ways to cope with competitive stress^44^ and maths anxiety^36,37^; emphasizing an incremental view of intelligence in efficient learning^49^; and implementing self-affirmation tasks to protect girls from stereotype threats^50^.

The present findings should enhance societal awareness of the absence of gender disparities in mathematical ability before children start school and their rapid emergence when formal teaching of mathematics begins, independently of age. Such awareness is a prerequisite to efforts, by parents as well as teachers, to encourage their children equally to build on their aptitudes for school mathematics and to pursue studies and professions relating to science, technology, engineering and mathematics^5,17,28^.

## Online content

Any methods, additional references, Nature Portfolio reporting summaries, source data, extended data, supplementary information, acknowledgements, peer review information; details of author contributions and competing interests; and statements of data and code availability are available at https://doi.org/10.1038/s41586-025-09126-4.

## Methods

### Inclusion and ethics statement

In 2018, 2019, 2020 and 2021/2022, all children in first or second grade in France were tested at school within their classroom. Once the data were anonymized, they were sent to the Department of Evaluation, Forecasting and Performance (DEPP, *Direction de l’évaluation, de la prospective et de la performance*), which is the national statistical institution of the Ministry of Education in France, and stored on approved European General Data Protection Regulation (GDPR) servers. The National Education Data Ethics Committee, composed of qualified members, ensures compliance with the legal framework regarding information given to parents and the protection and use of educational data. Parents were informed about these national assessments and the secondary use of the data for research. All children were tested, and parents can–whenever they so chose–refuse the use of their children’s data for further statistical purposes.

### Design and data acquisition

#### Purpose of the EvalAide programme

The EvalAide programme was designed by DEPP with the help of members of a dedicated subgroup of scientists, teachers, educators and inspectors from the French Scientific Council for Education. Inspired by similar programmes, such as the United Kingdom’s ‘Phonics check’, its purpose was to provide every French teacher in first or second grade with a detailed picture of the needs, achievement and progress of each child in their class, in both maths and language. At the beginning of first grade, the tests were used to detect children who were lagging behind in specific domains (for example, knowledge of Arabic numerals) or were at risk of developing developmental disorders such as dyslexia. In the middle of first grade and at the beginning of second grade, the tests monitored the children’s progress to examine if they were properly responding to pedagogical intervention, therefore allowing teachers to intensify their efforts and adapt their pedagogical strategies if progress was deemed insufficient. Parents were also informed of the test results. Teachers were encouraged to remit the results to parents during an individual parental meeting, thus fostering parent and teacher collaboration. In summary, the main goal of the programme was to help every child by identifying their specific and individual needs. Nevertheless, as a secondary goal, the data also permitted a fine-grained statistical monitoring of pedagogical performance in France.

#### Cohorts

We analysed four consecutive longitudinal French national assessment cohorts, targeting all French children entering first grade in 2018, 2019, 2020 or 2021, respectively. The total number of first-grade classes tested was 43,970 in 2018, 51,599 in 2019, 54,073 in 2020 and 54,224 in 2021. The increasing numbers of classes are the consequence of a political decision to reduce class sizes for priority education and higher-priority education schools, a decision that was progressively implemented during those 4 years. The four cohorts comprised, respectively, 610,905, 711,452, 743,734 and 804,989 children for a total of 2,871,080 children. Following French law, most children entered first grade in September of the year of their sixth birthday (see the description for age in first grade in the Supplementary Information). In France, first grade represents a shift, compared to kindergarten, associated with the beginning of a formal maths curriculum, whereas fluency, letter–sound associations, and decoding and writing letters are taught in kindergarten and pursued in first grade to follow the formal reading curriculum.

#### Data collection

Altogether, 46 tests were administered by teachers over a 12-month period. Assessments were implemented at three specific times: beginning of first grade (between the third and fourth weeks of September), hereafter called T1; middle of first grade (between the third and fourth weeks of January; T2) and beginning of second grade (between the third and fourth weeks of September; T3). Each test aimed to assess specific skills in oral language, reading, mathematics and problem-solving, as detailed in the Supplementary Information. Most tests were administered to the whole class, and children answered by circling targeted items or by writing in an individual notebook. The only exceptions were the 1-min reading aloud tests, which were administered individually. The duration of test sessions was about 35 min in language and 25 min in maths at T1, 35 min in language and 25 min in maths at T2, and up to 35 min in language and 30 min in maths at T3. In the days following testing, teachers and schools were responsible for entering every individual response into a dedicated computerized system. The data were copied and anonymized at the regional level and sent to the national level, where they were stored following European GDPR laws. The data were subject to various controls, such as deletion of duplicates, comparisons with former datasets, and a control of correct and valid values for each variable by DEPP. All personal ID numbers were checked for errors.

Pilot studies were performed by DEPP in January and May 2018 to finalize the design of the tests, 8 months before the launch of the first cohort. More than 200 schools, both public (excluding priority education and higher-priority education schools) and private, participated in these pilot studies, which included 5,500 first graders and 300 teachers, educators and inspectors, who gave feedback on the tests. Those surveys were used to select the tests that were first implemented in September 2018 for the whole population of first graders in France.

In subsequent years, feedback from teachers, scientists, educators and inspectors from the French Scientific Council for Education was gathered and used to improve the tests. A few changes were made to the tests between the four cohorts. Two tests were withdrawn from 2018 (recognizing letters among symbols at T1 and reading pseudowords at T2), seven tests were slightly changed in either their ergonomics or the number of items they included between 2018 and the other years, one test was modified in 2021 (number line, as explained in the Supplementary Information) and two tests were added in 2019, 2020 and 2021 compared to 2018 (geometry at T1 and reading comprehension of sentences at T2). When we performed an analysis within a specific cohort, we used all the 44 common tests that were shared (with minimal variants) between the four cohorts, whereas for between-cohort comparisons, we considered only the 37 identical tests.

#### Test design

Maths items comprised number reading, number writing, enumerating quantities, number comparison, problem-solving, number line, addition, subtraction, mental calculation and geometry. Language items comprised oral comprehension of words, sentences and texts, phoneme manipulation, syllable manipulation, letter–sound association, letter recognition, visuo-attentional abilities, 1-min word reading, 1-min text reading, writing words to dictation, and reading comprehension of sentences and of texts. Details of each assessment and corresponding cognitive functions are briefly described in the Supplementary Information and Supplementary Table 4.

#### Child gender

Gender was registered in a binary manner as male or female and reported by the teacher. As recommended in the literature, the term ‘gender’ is used instead of ‘sex’ throughout this manuscript as the gender for each child was declared by an external person.

### Scoring

#### Scoring: normalization and Gaussianization

For the individual tests, the results were first expressed as percentage success (percentage of correct items ranging from 0 to 100), as distributions of score for individual tests were discretized due to the small number of items and were often far from a normal distribution. Percentage success on individual tests was used in the matching selection process (‘Matching techniques’). However, as the tests evolved in nature and difficulty from T1 to T2 to T3, the scores could not be directly compared among the three periods. Therefore, after normalizing all scores by first computing the mean percentage success across all maths tests (respectively, language), we then transformed this mean into a *z* score through Gaussianization (variables were centred with a mean of 0 and a s.d. of 1). We used the function gaussianize in the LambertW package in the software R. Comparing the *z* scores across T1, T2 and T3 allowed us to monitor a child’s progress relative to others. These Gaussianized data, thereafter called ‘maths at T*x* (*z* score)’ and ‘language at T*x* (*z* score)’, were used for all multilevel regression models and sensitivity analyses. When comparing boys and girls, the effect size (which is an estimate of the size of the gender gap) was measured using Cohen’s *d* using the function cohens_d in the rstatix package in R.

#### Scoring: percentile ranks

To visualize the distribution of boys and girls, in Figs. 1c and 4b and Extended Data Fig. 5b, we also found it useful to examine the rank of each participant within their year’s population. To this aim, we transformed the mean language and maths scores into percentile ranks using the R function rank with the option ties.method set to average. This option meant that when two or more children had numerically identical mean scores, they were assigned the mean rank of their score (for example, if two children had the same top score, instead of arbitrarily ranking them as 1 and 2, they were assigned rank 1.5). Percentile ranks ranged from 0 to 100, 0 being the worst and 100 being the best rank. Using percentile ranks has two advantages: (1) Like *z* scores, we can compare maths and language tests among T1, T2 and T3 even though the tests were different from one period to the next. (2) This makes the distributions of boys and girls at the low end and high end of the distributions more visible (see distributions in Fig. 1c and Extended Data Figs. 4b and 5b).

#### Other variables

Variables at the individual level comprised child gender, which was reported by teachers, child age at T1 and child age category. Variables at the class level comprised the following: class size (number of children per class) ranging from 6 to 27 children per class, in line with the referenced STAR experiment; class mean in maths or language per gender; class mean in maths or language; heterogeneity in maths or language per class at T1; proportion of boys per class; gender of the first-in-class in maths or language at T1; and class mean in maths or language without the mean of the first-in-class. Variables at the school level comprised the type of school and SES. Details of all other variables are presented in the Supplementary Information.

#### Data-processing workflow

All analyses were implemented in the software R, using slightly different pipelines to consider the small differences between the three cohorts. All scripts can be found in the following GitHub repository: PauMdlm/Gendergaps.

### Outlier management

#### Age outliers

When aberrant birthdates were identified (for example, a child registered as born in 2018 and thus supposedly entering first grade at the age of 2), their age was replaced by a missing value (not available, NA). A total of 169, 310, 261 and 446 children had aberrant birthdates in 2018, 2019, 2020 and 2021, respectively. Ages outside of 51–98 months were replaced by NA.

#### Missing values for an entire session

A child who was absent from school on the day of the assessment was assigned either zeros or missing values in maths or language for the assessment period. When maths or language tests contained missing values or zeros for a whole session while having plausible results elsewhere, only the scores for this specific session were replaced by NA’s, and the other two test sessions were kept as valid. All students with at least one valid test session (T1, T2 or T3) were kept in our analysis. A total of 75, 101, 128 and 1,222 children were excluded because all sessions were missing in 2018, 2019, 2020 and 2021, respectively.

#### Class size

When class size contained aberrant values (more than 28 children per class), the class size was replaced by NA. This situation corresponded to 0.005% of the dataset in 2018, 2019 and 2020.

#### Missing values for gender

As our outcome was the gender gap between children, classes for which gender information was not available were removed from our analysis. A total of 60, 41, 135 and 0 children were removed in 2018, 2019, 2020 and 2021, respectively.

### Missing data imputation

Among the 2,871,080 children followed up from 2018 to 2022, 122,922, 140,580, 129,153 and 236,898 children (respectively, in 2018, 2019, 2020 and 2021), had at least one missing value on the different variables before outlier management. Supplementary Table 5 details the missing values and their proportions. Some imputation techniques, such as removing missing data or imputing by the mean, could have biased our analyses and conclusions, for instance because more data was missing from lower-SES schools. Therefore, we conducted an imputation by chained equations on all the missing values using the mice package in R.

### Statistical analyses

#### Statistical tests

Whenever quantitative variables were compared, Cohen’s *d* and Student’s *t* tests were calculated with the rstatix package in the software R v.4.3.2. When categorical variables were compared, chi-squared tests were implemented. R packages used included rstatix, FactoMineR, dplyr, tidyverse, broom, ggplot2, jtools, LambertW, cohens_d, reshape2, lmerTest, knitr, rmarkdown, MatchIt, remotes, rcpp, glmertree, BayesFactor, mice and tableone, all for R v.4.3.2. In addition, the regression discontinuity design was performed with Stata (v.18, 2023) using the package rdrobust.

#### Main analysis: multilevel multivariate mixed regression models

Multilevel multivariate mixed regression models were used to evaluate the association of gender and maths scores at T3, after controlling for several other variables (Table 3 and Supplementary Table 16). Similar regressions were performed on maths scores at T1 (Supplementary Table 17) and at T2 (Supplementary Table 18), as well as on language scores at T3 (Extended Data Table 2). As described in the section ‘Scoring: normalization and Gaussianization’, data used for multilevel modelling underwent normalization (ranging from 0 to 100), then Gaussianization (centred and reduced with a mean of 0 and a s.d. of 1). Of the independent variables, only gender remained unscaled.

In this study, the children were taught within classes, all nested within schools. Because of these different environments, data contained natural groupings, which impacted the performance of individual children. These various levels also implied that individual observations were not independently sampled.

Multilevel linear mixed models can overcome these two limitations of conventional models by accounting for nested sources of variation in the data and avoiding the assumption of independently sampled data. Mathematically, nested patterns were introduced in the intercept and in the slope at the class level. Using stepwise multilevel models allowed us to consider class effects (gender and maths at T1) as random effects.

Corresponding to the class effect, the second-level random part of the multilevel model was specified step by step, following a stepwise multilevel model (Supplementary Table 16): intercept, gender and maths at T1 variances as well as their respective covariances proved significance. Maths at T1 was introduced as a random variable as it was a strong predictor of maths at T3. Gender was also introduced as a random variable because it represented our variable of interest. We explored progressively more complex linear regression models, starting with the simplest, eventually adding individual, contextual and interaction terms, as presented in Supplementary Table 16. The decrease in deviance represented the significance of the model (deviance for model 1 was 1,564,075.7 and for model 10, it was 1,158,166.8).

Multilevel linear mixed modelling, fitted by maximum likelihood, was performed using the R package (v.4.3.2) lmerTest, which allowed us to estimate several individual and environmental parameters regarding the gender gap (Table 3).

#### Matching techniques

Matching techniques were used to test the effect of school exposure on the growing gender gap. The idea was to identify pairs of individuals who were very closely matched according to initial characteristics and differed in only one parameter (gender). We could then estimate how, as everything else was essentially identical, this variable alone led to a distinct outcome on subsequent data points (mathematical performance at T3).

Pairs were matched on school type (private and regular public versus priority education and higher-priority education), on deciles of SES score, on age in first grade (±4 months), on the six tests in maths at T1 (±5 points over 100), as well as on their mean in language at T1 (±5 points over 100). Results are shown in Supplementary Table 14 and in Extended Data Fig. 7.

The number of pairs found and their means are shown in Extended Data Fig. 7 and Supplementary Table 14. For those analyses, we used the package MatchIt in the software R (v.4.3.2). Once the matching was performed, a simple test for a difference in means between boys and girls was enough when using exact matching, but to adjust for any potential remaining imbalance, we used linear regression to estimate the effect. Results are detailed in Supplementary Table 14.

### Reporting summary

Further information on research design is available in the Nature Portfolio Reporting Summary linked to this article.

## Extended Data

**Extended Data Table 1:** Results of causal inference methods applied to the gender gap in Math between T1 and T3 (difference in z-score, not in Cohen’s d) (n = 2,653,082 children)

	2018			2019			2020			2021		
	Average gender effect in math between T3 and T1 (Z-score)	Lower Cl	Upper Cl	Average gender effect in math between T3 and T1 (Z-score)	Lower Cl	Upper Cl	Average gender effect in math between T3 and T1 (Z-score)	Lower Cl	Upper Cl	Average gender effect in math between T3 and T1 (Z-score)	Lower Cl	Upper Cl
G-computat on (OLS)	**0.3061**	0.2946	0.3175	**0.2889**	0.2788	0.2990	**0.2847**	0.2747	0.2947	**0.2872**	0.2774	0.2969
IPW (logit)	**0.3282**	0.3230	0.3334	**0.2904**	0.2856	0.2952	**0.2974**	0.2926	0.3021	**0.2895**	0.2849	0.2942
Propensity weighted regression	**0.3282**	0.3230	0.3333	**0.2904**	0.2856	0.2952	**0.2973**	0.2926	0.3020	**0.2895**	0.2849	0.2941
AIPW (OLS & logit)	**0.3280**	0.3243	0.3317	**0.2905**	0.2872	0.2939	**0.2973**	0.2940	0.3007	**0.2896**	0.2863	0.2928
Random forest	**0.3211**	0.3174	0.3248	**0.2878**	0.2844	0.2912	**0.2936**	0.2903	0.2970	**0.2882**	0.2849	0.2915
TMLE (Target Maximum Likelihood Est mat on) for causal inference	**0.3279**	0.3242	0.3316	**0.2903**	0.2870	0.2937	**0.2971**	0.2938	0.3005	**0.2893**	0.2860	0.2926

Note: CI = 95% confidence interval.

**Extended Data Table 2:** Multilevel regression model for Language at T3 among children of typical age at T1 (n = 2,653,082 children)

	Language at T3							
Variables	2018		2019		2020		2021	
N	569,771		665,632		695,449		722,230	
N group (classes)	39,573		46,671		49,010		49,701	
Fixed effects	Parameter est mates (sd)	p	Parameter est mates (sd)	p	Parameter est mates (sd)	P	Parameter est mates (sd)	p
Intercept	0.0459 (0.0026)	< 0.0001	0.0606 (0.0029)	< 0.0001	0.0454 (0.0028)	< 0.0001	0.0272 (0.0029)	< 0.0001
Language individual level at T1	0.5759 (0.0015)	< 0.0001	0.5764 (0.0018)	< 0.0001	0.5684 (0.0018)	< 0.0001	0.5175 (0.0019)	< 0.0001
Math individual level atTl	0.1622 (0.0013)	< 0.0001	0.1451 (0.0017)	< 0.0001	0.1559 (0.0017)	< 0.0001	0.1672 (0.0018)	< 0.0001
Gender (Boys)	-0.0328 (0.0039)	< 0.0001	-0.0337 (0.0036)	< 0.0001	-0.0386 (0.0035)	< 0.0001	-0.0168 (0.0037)	< 0.0001
SES score at T1	0.0667 (0.0020)	< 0.0001	0.1137 (0.0020)	< 0.0001	0.0709 (0.0020)	< 0.0001	0.0716 (0.0019)	< 0.0001
Age at T1 (month)	-0.0043 (0.0003)	< 0.0001	-0.0045 (0.0004)	< 0.0001	-0.0014 (0.0003)	0.0001	-0.0002 (0.0004)	0.4927
Class size	-0.0029 (0.0020)	0.1464	-0.0047 (0.0019)	0.0157	-0.0076 (0.0019)	0.0001	-0.0014 (0.0019)	0.4583
First of class is a boy in language at T1	-0.0006 (0.0019)	0.7532	0.0004 (0.0018)	0.8440	-0.0001 (0.0018)	0.9503	-0.0006 (0.0018)	0.7293
Boys – Girls rat o per class at T1 Heterogeneity of level in language atTl Gender * Language atTl	0.0011 (0.0018)	0.5429	-0.0027 (0.0018)	0.1390	-0.0053 (0.0018)	0.0029	0.0024 (0.0018)	0.1665
	-0.0234 (0.0018)	< 0.0001	-0.0125 (0.0017)	< 0.0001	-0.0120 (0.0017)	< 0.0001	-0.0104 (0.0017)	< 0.0001
	-0.0070 (0.0025)	0.0059	-0.0030 (0.0024)	0.2011	-0.0054 (0.0024)	0.0229	-0.0076 (0.0024)	0.0019
Gender * MathatTl	0.0021 (0.0025)	0.3926	-0.0015 (0.0023)	0.5075	0.0048 (0.0023)	0.0402	0.0026 (0.0024)	0.2798
Gender * SES score	-0.0061 (0.0021)	0.0031	-0.0102 (0.0019)	< 0.0001	-0.0121 (0.0019)	< 0.0001	-0.0109 (0.0019)	< 0.0001
Gender * Age at T1	0.0006 (0.0005)	0.2764	0.0001 (0.0005)	0.9137	0.0001 (0.0005)	0.8688	0.0001 (0.0005)	0.7818
Gender * Class size	0.0046 (0.0020)	0.0221	0.0032 (0.0018)	0.0779	0.0033 (0.0018)	0.0767	0.0051 (0.0019)	0.0059
Gender * First of class is a boy in Language atTl	0.0107 (0.0019)	< 0.0001	0.0003 (0.0017)	0.8509	0.0047 (0.0017)	0.0061	0.0059 (0.0018)	0.0010
Gender * Boys-Girls rat o per class	-0.0041 (0.0020)	0.0392	0.0022 (0.0018)	0.2195	-0.0031 (0.0018)	0.0811	-0.0062 (0.0019)	0.0009
Gender * Heterogeneity of level in languageatTl	0.0051 (0.0018)	0.0058	0.0033 (0.0017)	0.0505	-0.0008 (0.0017)	0.6403	0.0034 (0.0017)	0.0492
Random ef ects								
Between-class variance (Level 2)								
Intercept variance	0.0990		0.0803		0.0803		0.0749	
Gender variance	0.0060		0.0044		0.0057		0.0061	
Language at T1 variance Correlaton Intercept | Gender	0.0083		0.0055		0.0085		0.0136	
	-0.11		-0.25		-0.22		-0.18	
Correlaton Intercept | Language at T1	0.24		0.18		0.08		-0.08	
Correlat on Gender | Language at T1	0.09		0.18		0.21		0.02	
Within-class variance (Level 1)	0.4268		0.4229		0.4242		0.4775	
Deviance (-2 log L)	1196878.7		1381396.8		1449135.7		1587511.5	

Note: The formula implemented was as follow: Language at T3 ~ Age at T1 + Gender + Math level at T1 + language level at T1 + First of class being a boy in language + SES score at T1 + Class size + Boys-Girls ratio per class + Heterogeneity of level in language in the class + 8 interactions between each variable and Gender + (1 + Gender + Language level at T1 | class).

**Extended Data Table 3:** Different regression models comparing the size of the math gender gap between T2 and T3 in function of the year, gender and their interaction, between one cohort and the consecutive one (n = 2,653,082 children)

	T3-T2 Gender gap in math					
	Comparing 2018 and 2019		Comparing 2019 and 2020		Comparing 2020 and 2021	
	Parameter est mates (sd)	p	Parameter est mates (sd)	p	Parameter est mates (sd)	p
***Intercept***	0.0024 (0.0010)	0.0176	0.0040 (0.0009)	***<0.0001***	0.0033 (0.0009)	0.0004
Year (relat ve to previous)	0.0016 (0.0014)	NS (0.2532)	-0.0007 (0.0013)	NS (0.5916)	-0.0018 (0.0013)	NS (0.1665)
Main ef ect of gender	0.1803 (0.0020)	<0.0001	0.1118 (0.0018)	<0.0001	0.1256 (0.0018)	<0.0001
Gender*Year	**-0.0685 (0.0027)**	**<0.0001**	**0.0139 (0.0026)**	**<0.0001**	**0.0083 (0.0026)**	**0.0012**

## Supplementary Material

The online version contains supplementary material available at https://doi.org/10.1038/s41586-025-09126-4.

Supplementary Information

Supplementary Materials

## Figures and Tables

**Table 1:** Cohen’s *d* effect size for gender gaps in 2018, 2019, 2020 and 2021 among children of typical age at T1 (*n*_Total_ = 2,653,082 children)

Domain	Year	T1	T2	T3
Maths	2018	−0.0166	0.0468	0.2230
	2019	0.0127	0.0895	0.1938
	2020	0.0082	0.0832	0.1974
	2021	0.0066	0.0698	0.2037
Problem-solving	2018	−0.0546	0.0296	0.1040
	2019	−0.0212	0.0362	0.1030
	2020	−0.0224	0.0362	0.1096
	2021	−0.0239	0.0189	0.1364
Number line	2018	0.0271	0.0915	0.2588
	2019	0.0453	0.1085	0.2595
	2020	0.0487	0.1102	0.2731
	2021	0.0364	0.1105	0.1729
Language	2018	−0.1935	-0.0845	−0.1371
	2019	−0.1818	-0.0768	−0.1283
	2020	−0.1770	-0.0756	−0.1319
	2021	−0.1720	-0.0707	−0.0985

All results were statistically significant.

**Table 2:** Cohen’s *d* for gender for each test and for all four cohorts (2018, 2019, 2020 and 2021) among children of typical age at T1 (*n*_Total_ = 2,653,082 children)

	Effect size (Cohen’s *d)*			
Year	2018	2019	2020	2021
*n*	569,771	665,632	695,449	722,230
Oral comprehension of words at T1	−0.0795	−0.0730	−0.0698	−0.0746
Oral comprehension of sentences at T1	−0.2567	−0.2410	−0.2448	−0.2373
Oral comprehension of texts at T1	−0.1327	−0.1330	−0.1386	−0.1292
Phoneme manipulation at T1	−0.1005	−0.1047	−0.1061	−0.0948
Syllable manipulation at T1	−0.1562	−0.1364	−0.1337	−0.1307
Letter-sound association at T1	−0.1386	−0.1225	−0.1124	−0.1074
Recognizing letters at T1	−0.1373	−0.1319	−0.1279	−0.1263
Comparing letters at T1	−0.1144	−0.1162	−0.1212	−0.1322
Oral comprehension of sentences at T2	−0.1614	−0.1536	−0.1606	−0.1555
Reading words at T2 (time limited to 1min)	**0.0850**	**0.0958**	**0.0909**	**0.0735**
Reading texts at T2 (time limited to 1min)	**0.0328**	**0.0491**	**0.0508**	**0.0384**
Writing syllables at T2	−0.1004	−0.0937	−0.0897	−0.0768
Writing words at T2	−0.0999	−0.0979	−0.0905	−0.0993
Phoneme manipulation at T2	−0.0781	−0.0862	−0.0917	−0.0806
Letter-sound association at T2	−0.0797	−0.0683	−0.0737	−0.0462
Oral comprehension ofwords at T3	−0.0896	−0.0881	−0.0905	−0.0877
Oral comprehension of sentences at T3	−0.1802	−0.1786	−0.1764	−0.1821
Writing syllables at T3	−0.1062	−0.1002	−0.1064	−0.0910
Writing words at T3	−0.1413	−0.1410	−0.1415	−0.1022
Reading comprehension of sentences at T3	−0.1205	−0.1111	−0.1152	−0.0825
Reading comprehension of texts at T3	−0.1938	−0.1845	−0.1864	−0.1542
Reading words at T3 (time limited to 1min)	**0.0376**	**0.0318**	**0.0367**	**0.0609**
Reading texts at T3 (time limited to 1min)	−0.0473	−0.0372	−0.0362	**0.0210**
Writing numbers at T1	−0.0838	−0.0669	−0.0586	−0.0636
Reading numbers at T1	−0.0496	−0.0465	−0.0412	−0.0342
Problem-solving at T1	−0.0546	−0.0212	−0.0224	−0.0239
Enumerating quantities at T1	−0.0588	−0.0645	−0.0623	−0.0556
Comparing numbers at T1 (time limited to1 min)	**0.0849**	**0.0907**	**0.0729**	**0.0713**
Number line at T1	**0.0271**	**0.0453**	**0.0487**	**0.0364**
Comparing numbers at T2 (time limited to 1 min)	**0.0373**	**0.0662**	**0.0566**	**0.0516**
Number line at T2	**0.0915**	**0.1085**	**0.1102**	**0.1105**
Addition at T2	**0.0180**	**0.0554**	**0.0437**	**0.0204**
Subtraction at T2	−0.0185	**0.0545**	**0.0527**	**0.0424**
Writing numbers at T2	**0.0751**	**0.0836**	**0.0750**	**0.0595**
Problem-solving at T2	**0.0296**	**0.0362**	**0.0362**	**0.0189**
Geometry at T3	−0.0348	−0.0377	−0.0340	−0.0283
Number line at T3	**0.2588**	**0.2595**	**0.2731**	**0.1729**
Addition at T3	**0.3157**	**0.2643**	**0.2615**	**0.2407**
Subtraction at T3	**0.2385**	**0.1895**	**0.1882**	**0.1775**
Mental calculation at T3	−0.0808	−0.0784	−0.0867	−0.0742
Writing numbers at T3	**0.1382**	**0.1385**	**0.1302**	**0.2140**
Reading numbers at T3	**0.2053**	**0.1901**	**0.1913**	**0.2141**
Associating numbers and quantities at T3	**0.0491**	**0.0254**	**0.0280**	-
Problem-solving at T3	**0.1040**	**0.1030**	**0.1096**	**0.1364**
**Composite variables**				
Language level at T1	−0.1935	−0.1818	−0.1770	−0.1720
Language level at T2	−0.0845	−0.0768	−0.0756	−0.0707
Language level at T3	−0.1371	−0.1283	−0.1319	−0.0985
Maths level at T1	−0.0166	**0.0127**	**0.0082**	**0.0066**
Maths level at T2	**0.0468**	**0.0895**	**0.0832**	**0.0698**
Maths level at T3	**0.2230**	**0.1938**	**0.1974**	**0.2037**

For each subtest, significant Cohen’s *d* results for boys (versus girls) are highlighted in bold. All results are statistically significant.

**Table 3:** Multilevel mixed regression analysis of fixed and random T1 factors associated with children’s maths scores at T3 for typical-aged children (*n*_Total_ = 2,653,082 children)

Variables	Individual maths level at T3							
Year	2018		2019		2020		2021	
*N* individuals	569,771		665,632		695,449		722,230	
N groups (classes)	39,573		46,671		49,010		49,701	
**Fixed effects**	**Estimate**	***P***	**Estimate**	***P***	**Estimate**	***P***	**Estimate**	***P***
Intercept	*0.0107(0.0019)*	<0.0001	*-0.1408 (0.0028)*	*<0.0001*	*−0.1693 (0.0028)*	*<0.0001*	*−0.1739 (0.0028)*	*<0.0001*
Language individual level at T1	0.4078 (0.0013)	<0.0001	0.4164 (0.0017)	<0.0001	0.3946 (0.0017)	<0.0001	0.3861 (0.0017)	<0.0001
Maths individual level at T1	0.3810 (0.0013)	<0.0001	0.3306 (0.0017)	<0.0001	0.3506 (0.0018)	<0.0001	0.3738 (0.0017)	<0.0001
Gender (boys)	0.3285 (0.0018)	<0.0001	0.2975 (0.0035)	<0.0001	0.3099 (0.0035)	<0.0001	0.3025 (0.0034)	<0.0001
SES score at T1	0.0277 (0.0020)	<0.0001	0.0646 (0.0019)	<0.0001	0.0055 (0.0019)	0.0047	0.0001 (0.0019)	NS (0.9547)
Age at T1 (month)	0.0062 (0.0009)	<0.0001	0.0008 (0.0003)	0.0139	0.0049 (0.0003)	<0.0001	0.0062 (0.0003)	<0.0001
Heterogeneity of level in maths at T1	−0.0305 (0.0018)	<0.0001	−0.0187 (0.0017)	<0.0001	−0.0259 (0.0017)	<0.0001	−0.0242 (0.0017)	<0.0001
Ratio of boys to girls per class	0.0005 (0.0018)	NS (0.7742)	−0.0024 (0.0018)	NS (0.1677)	−0.0057 (0.0018)	0.0012	0.0001 (0.0018)	NS (0.9480)
First-in-class in maths is a boy at T1	0.0063 (0.0019)	0.0008	0.0068 (0.0018)	0.0002	0.0043 (0.0018)	0.0172	0.0052 (0.0018)	0.0036
Class size	0.0095 (0.0020)	<0.0001	0.0065 (0.0019)	0.0007	0.0069 (0.0019)	0.0003	0.0115 (0.0019)	<0.0001
Gender × language individual level at T1	−0.0065 (0.0024)	0.0075	−0.0138 (0.0023)	<0.0001	−0.0206 (0.0023)	<0.0001	−0.0310 (0.0023)	<0.0001
Gender × maths individual level at T1	0.0644 (0.0024)	<0.0001	0.0655 (0.0023)	<0.0001	0.0707 (0.0023)	<0.0001	0.0552 (0.0022)	<0.0001
Gender × SES score at T1	0.0049 (0.0020)	0.0146	−0.0014 (0.0019)	NS (0.4486)	0.0060 (0.0019)	0.0016	0.0052 (0.0018)	0.0039
Gender × age at T1	−0.0094 (0.0019)	<0.0001	−0.0013 (0.0005)	0.0071	−0.0023 (0.0005)	<0.0001	−0.0026 (0.0005)	<0.0001
Gender × heterogeneity of level at T1	−0.0048 (0.0018)	0.0081	−0.0040 (0.0017)	0.0160	−0.0013 (0.0017)	NS (0.4236)	−0.0049 (0.0016)	0.0029
Gender × ratio of boys to girls per class	−0.0010 (0.0020)	NS (0.6124)	0.0037 (0.0018)	0.0363	−0.0005 (0.0018)	NS (0.7770)	−0.0045 (0.0018)	0.0110
Gender × First-in-class in maths is a boy at T1	0.0064 (0.0019)	0.0006	0.0030 (0.0017)	NS (0.0799)	0.0066 (0.0017)	0.0001	0.0062 (0.0017)	0.0002
Gender × class size	0.0043 (0.0020)	0.0275	0.0047 (0.0018)	0.0095	0.0010 (0.0018)	NS (0.5958)	0.0030 (0.0017)	NS (0.0821)
**Random effects**								
Between-class variance (level 2)								
Intercept between-class variance	0.1003		0.0798		0.0839		0.0845	
Gendervariance	0.0091		0.0049		0.0086		0.0071	
Maths at T1 variance	0.0046		0.0033		0.0042		0.0032	
Correlation intercept | gender	0.13		0.02		−0.06		−0.22	
Correlation intercept | maths at T1	0.32		0.32		0.22		0.36	
Correlation gender | maths at T1	−0.31		−0.22		−0.23		−0.51	
Within-class variance (level 1)	0.3982		0.3986		0.4082		0.4195	
Deviance (−2 log *L)*	1,158,166.8		1,344,146.4		1,423,858.3		1,493,119.3	

All variables were normalized and Gaussianized. Therefore, each beta reflects an effect size. The lmerTests used for these multilevel mixed regression analyses were two-sided. Significant interactions between factors and gender gap are indicated in bold. NS, not significant.

## Data Availability

The original data were collected on a national scale and centralized by the DEPP team at the Ministry of Education in France. A formal agreement was established between our research laboratory and DEPP to enable our local use of and access to the data. We used the software R v.4.3.2, which can be found at the following link: https://cran.r-project.org/bin/windows/base/. Given that the data adhered to the GDPR European law on data protection, extraction of data from the DEPP structure was not permitted. For confidentiality, the raw data are not shared in public but are made accessible through a data security convention established with DEPP in France. We shared a simulated dataset to gain an initial understanding of how the data were organized and how the data management was structured.

## Code availability

For reproducibility, the code and models that were used to generate the results, text, figures and tables both in the main text and in the Supplementary Information are publicly available on the GitHub repository https://github.com/PauMdlm/Gendergaps.
