# Supplementary material for: Rapid emergence of a maths gender gap in first grade
Source: Nature. Author manuscript; Available in PMC 2025 Dec 11. (PMC7618463; doi:10.1038/s41586-025-09126-4)
Supplement: Supplementary Information [file EMS210438-supplement-Supplementary_Information.pdf]

nature portfolio

https://doi.org/10.1038/s41586-025-09126-4

## Supplementary information

# Rapid emergence of a maths gender gap in first grade

In the format provided by the authors and unedited

# Supplementary information

**Manuscript title**: **Rapid Emergence of a Math Gender Gap in First Grade**


### Authors

MARTINOT, P.[1,2*], COLNET B.[3], BREDA T.[4], SULTAN J.[5], TOUITOU L.[5], HUGUET P.[6], SPELKE E.[7,8] DEHAENE-LAMBERTZ G.[2], BRESSOUX P.[9], DEHAENE S.[2,10]*

### Affiliations

[1] University Paris Cité, 45 rue des Saints-Pères, 75006 Paris

[2] Cognitive Neuroimaging Unit, CEA, INSERM, Université Paris-Saclay, NeuroSpin center, 91191 Gif/Yvette, France

[3] Soda project-team, Premedical project-team, INRIA Paris-Saclay, 1 Rue Honoré d'Estienne d'Orves, 91120 Palaiseau, France

[4] PSE - Paris School of Economics, 48 boulevard Jourdan 75014 Paris, France

[5] Institute for Public Policies IPP, 48 boulevard Jourdan, 75014 Paris, France

[6] University of Clermont Auvergne and CNRS, LAPSCO, F-63000 Clermont-Ferrand, France

[7] Harvard University, Department of Psychology, Cambridge, MA, 02138, USA

[8] The Center for Brains, Minds and Machines, Cambridge, MA 02139, USA

[9] University Grenoble Alpes, LaRAC, 38000, Grenoble, France

[10] Collège de France, Université Paris-Sciences-Lettres (PSL), 11 Place Marcelin Berthelot, 75005 Paris, France

* Corresponding authors: pauline.martinot.dlm@gmail.com and stanislas.dehaene@cea.fr

**Supplementary Information Guide.**

This Supplementary Information document provides additional methodological details, robustness checks, and results for the analyses reported in the main manuscript. It includes 1) **Supplementary Methods** with comprehensive details on test designs and additional variables used; 2) **Statistical Analyses and Results** with a step-by-step presentation of the modeling strategies, including multilevel models and sensitivity checks; 3) **Supplementary Tables** (S6–S21) with details of data management and supplementary model analyses (descriptive information, Bayes Factors, growth models, regression analysis) ; 4) **Supplementary Figures** (S1-S2) with checks for the regression discontinuity design; 5) **Supplementary Discussion** including an additional contextual analysis of the findings, particularly regarding the education system in France. Each supplementary component is precisely referenced in the main article (e.g., “see SI Table S9”).

## Table of contents


**Authors** ........ **1**

**Affiliations** ........ **1**

**Supplementary Information Guide.** ........ **2**

**Supplementary Methods** ........ **4**
- **Test design** ........ 4
- **Math abilities** ........ 4
- **Language abilities** ........ 5
- **Additional variables** ........ 7

**Statistical analyses and supplementary results.** ........ **11**
- **Sensitivity analysis** ........ 11
- **Gender gap effect size in Cohen's d** ........ 11
- **Gender gap measures for each specific subtest in math and language, comparing boys and girls** ........ 11
- **Main analysis: Multilevel multivariate mixed regression models at T3** ........ 12
- **Additional results of modelling math at T1 using a multi-level regression model** ........ 13
- **Additional results of modelling math at T2 using a multi-level regression model** ........ 13
- **Additional results of modelling language at T3 using a multi-level regression model** ........ 13
- **Growth models** ........ 14
- **Bayes Factor for testing null hypothesis** ........ 14
- **Gender gap for individual math subtests and relation to test periods and test difficulty (Table S7)** ........ 15
- **Additional analyses and figures at the class level (Extended Data Figure 4)** ........ 16
- **Matching and reweighting (Extended Data Table 1, Table S14, Extended Data Figure 7)** ........ 17
- **Comparing gender gaps' magnitudes between 2018, 2019, 2020 and 2021 (Extended Data Table 3)** ........ 18
- **Comparison of children with similar age but one-year difference in schooling exposure (Table S8)** ........ 18
- **Regression Discontinuity Design (RDD)** ........ 20
- **Supplementary methods bibliography** ........ 22

**Supplementary Tables** ........ **23**
- **Supplementary Table S4.** Summary of cognitive tests evaluated at each period. ........ 23
- **Supplementary Table S5.** Overview of missing values in the four cohorts and their data management. ........ 24
- **Supplementary Table S6.** Modeling the data using a growth framework, with time nested in students, nested in classes in 2018. ........ 25
- **Supplementary Table S7.** Relationship between Math subtest level, gender gaps, test periods and test difficulty (math subtests only) ........ 26
- **Supplementary Table S8.** Comparison of the magnitude of gender gaps for children born one-month apart. ........ 27
- **Supplementary Table S9.** Description of children born few months apart but with 1 school year of difference, between 2018 and 2019, 2019 and 2020 and 2020 and 2021. ........ 28
- **Supplementary Table S10.** First stage LATE estimates: effect of day of birth around cutoff on age at math test ........ 29
- **Supplementary Table S11.** Effect (LATE) of day of birth around cutoff on test scores ........ 30
- **Supplementary Table S12:** Effect (LATE) of day of birth around cutoff on test scores with donut-hole ........ 31
- **Supplementary Table S13.** Effect (LATE) of day of birth around cutoff on average school SES and the share of boys in class ........ 32
- **Supplementary Table S14.** Design of the matching experiments presented on Extended Data Figure 7 (n = 2,653,082 children). ........ 34
- **Supplementary Table S15.** Sensitivity analysis of the imputed population in 2018 (n = 586,949). ........ 35
- **Supplementary Table S16.** Progressive multilevel modelling of the mean Math score at T3 among children of typical age in first grade, 2018 cohort (n = 569,771). ........ 36
- **Supplementary Table S17.** Multilevel regression model for Math at T1 among children of typical age at T1. ........ 38
- **Supplementary Table S18.** Multilevel regression model for Math at T2 among children of typical age at T1. ........ 39
- **Supplementary Table S19.** Bayes Factors for the null hypothesis of Math at T1 and of Math at T3. ........ 40

**Supplementary Figures** ........ **41**

**Supplementary Discussion** ........ **43**
- **Supplementary discussion on the possible specificities of France** ........ 43

## Supplementary Methods

### Test design

All tests were synthesized and presented in the supplementary information (SI) Table S4.

### Math abilities

**Number reading (T1, T3).** In this test, the teacher stated a number orally, and the student had to choose and circle the corresponding Arabic number among 6 possibilities. 10 different numbers were assessed (0-10 at T1, and up to 99 at T3).

**Number writing (T1, T2, T3).** The teacher stated a number orally and the student had to write it down. The numbers went from 0 up to 10 at T1, 31 at T2, and up to 100 at T3. Children were exposed to 11, 10 and 10 read-aloud-numbers, respectively at T1, T2, T3.

**Enumerating a concrete set (T1, T3).** Children viewed a collection of eggs in a basket and had to select the corresponding Arabic numeral on a number line with 10 cells marked from 1 to 10.

**Number comparison (T1, T2).** Students had to cross out the larger of two Arabic numerals, presented side by side. The assessment at T1, adapted from the *Belgian Symp test*[51], included 60 pairs of numbers between 0 and 9, half of the pairs being distant by one unit, the other by 3 to 4 units. There was a time limit of 1 minute, after which the test was stopped. This assessment was replicated at T2 to assess student's progress, with 40 items, and with a time limit of 1 minute.

**Problem solving (T1, T2, T3).** Problem solving simultaneously involved language comprehension and arithmetic skills. In this task, students heard an oral arithmetic problem read by the teacher (at T1, T2, T3), and also had the possibility to read the corresponding written sentences (at T3) – for instance "Lucie had one marble, and now she has seven. How many marbles did she win?". The child had to find the correct answer among 6 choices. 5 items were shown at T2, and 6 at T1 and T3. The numbers involved respected the range of numbers introduced in the national curriculum: numbers below ten at T1 and T2, and 2-digit numbers at T3. All the statements were read by the teacher, and children had one minute and thirty seconds to respond to each of them.

**Number line (T1, T2, T3).** On each trial, the child saw an ungraduated horizontal line marked at both ends with some reference numbers (e.g., 0 at left and 10 at right). One location was marked with a vertical bar and a diamond shape. The children had to figure out which number corresponded to this bar, and to select it among 6 possible choices that were proposed, in randomized order, below the line. An example appears at right:

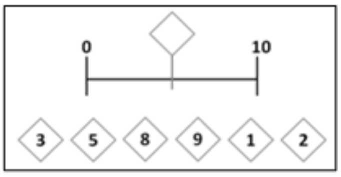


At T1, all 6 target lines ranged from 0 to 10. At T2 (10 items) and T3 (15 items), the endpoint labels could vary and could include 2-digit numbers,

and therefore exposed students to problems of different levels of difficulty. For example, one item involved finding the middle of two close numbers (12 and 14), another the middle of two numbers that were further apart (2 and 6), and a third required finding how to proceed when the segment was not in the middle (e.g., 17 when the line goes from 10 to 20). All tests were identical in 2018, 2019, 2020 and 2021, but only at T3 and only in year 2021, the test was changed from "placing a number on a line", towards "placing a number on a line".

**Addition (T2, T3).** Addition problems were presented in written form in Arabic numbers to the children (e.g., "20 + 30 = "). They had to select the correct answer among 6 choices. 7 problems were presented at T2 and T3 in 2018, and respectively 10 and 8 problems at T2 and T3, in 2019, 2020 and 2021.

**Subtraction (T2, T3).** Subtraction problems were similarly presented. There were 7 (at T2) and 8 (at T3) problems in 2018, and respectively 10 and 7 problems at T2 and T3 for years 2019, 2020 and 2021.

**Mental calculation (T3).** Students were asked to perform arithmetic calculations without the support of a written medium. 10 elementary spoken arithmetic problems were presented to the child (e.g., "ten minus two"), who had to select the correct answer among 6 choices.

**Geometry (T1, T3).** The geometry tests were only introduced at T3 in 2018, and at both T1 and T3 in 2019, 2020 and 2021. The tests were adapted from a prior intruder test[52]. In each of 16 boards (plus a practice one), children had to identify the intruder item among 6 possible choices, i.e. the shape that deviated from the others in a certain geometric property. The different boards evaluated the concepts of straight line, parallelism, mirror image, right angle, distance, circle, alignment, and spacing.

**Language abilities**

**Oral comprehension of words (T1, T3).** In a word-to-picture matching task, children had to circle, among 4 pictures, the one that matched the word read aloud by the teacher. Two of the distractor images had either a pronunciation close to that of the target word, or a semantic relationship with the target word (i.e., having the same function or category). The test consisted of 15 words.

**Oral comprehension of sentences (T1, T2, T3).** This test was adapted from the *Test for Reception of Grammar (TROG)* in English. Children had to circle, among 4 pictures, the one that matched the sentence read aloud by the teacher. Different kinds of syntactical structures were used, containing for instance spatial prepositions, active or passive sentences. A total of 15, 14 and 16 sentences were tested at T1, T2 and T3 respectively.

**Oral comprehension of texts (T1).** To assess the comprehension of spoken small texts, 18 different short stories at T1 (in 2018) and 11 different stories at T1 (in 2019,2020 and 2021) were read aloud by the teacher and were of increasing length and complexity. For every story, children had to circle the corresponding image among 4 items.

**Phoneme manipulation (T1, T2).** Children underwent two types of tests at T1: the first one was a series of 8 spoken words. For each word, children had to select, among 4 items, another word beginning with the same phoneme as the spoken word. The second series was of 7 spoken words. For each word, children had to select, among 4 items, another word that would end with the same phoneme as the spoken word. At T2, two series of 6 words each were presented to children, with the goal of identifying another word, among 4 items, that would begin and that would end (6 words each respectively) with the same phoneme than the spoken word.

**Syllable manipulation (T1, T2, T3).** Children underwent two tests at T1: the first one was a series of 10 spoken words. Children had to identify, among 4 items, another word beginning with the same syllable than the spoken word. The second was a series of 5 spoken words with the aim of identifying, among 4 items, another word which end would be the same syllable than the spoken word. At T2 and T3, children were also asked to write to dictation 10 or 12 simple and complex spoken syllables (respectively at T2 and T3) (e.g., mu, ti, na, lur, sar, ol, moi, che, tra, pli).

**Letter-sound association (T1, T2).** Children had to isolate the initial phoneme of a dictated word phoneme and had to circle its corresponding first letter, among 5 possible choices. The assessment consisted of 10 items.

**Letter recognition (T1).** Children had to circle, among 18 items, the 3 instances of a spoken letter which was read aloud by the teacher. The targets varied in font and case. The assessment consisted in a series of 7 such items.

**Visuo-attentional abilities (T1)**. To assess letters relative position to each other, children had to identify and circle the similar duo of letters, between 24 couples of letters, in a limited time. The assessment stopped after a duration of 2 minutes.

**One-minute word reading (T2, T3).** To evaluate word reading fluency, children were asked to read aloud as many words as they could, within one minute. The items in each list were presented in increasing order of difficulty. A maximum of 30 words were presented at T2, and 60 words at T3.

**One-minute text reading (T2, T3).** To evaluate text reading fluency, children were asked to read aloud, in less than 1 minute, as many words as they could within a text of 29 words (T2) or 102 words (T3).

**Writing words to dictation (T2, T3).** To assess writing abilities, children were asked to write 8 dictated simple and regular words at T2, and 12 regular words at T3.

**Reading comprehension of sentences (T2, T3).** Children had to circle, among 4 pictures, the one corresponding to the sentence they read. 8 sentences were presented at T2 in 2019,2020 and 2021, and 10 sentences of increasing length and complexity at T3 for all cohorts.

**Reading comprehension of texts (T3).** The test consisted of 8 short written texts of increasing length and complexity. For each text, children had to answer a question asked by the teacher, and had to circle, among 4 pictures, the one corresponding to the text they had read.

**Additional variables**

*Variables at the individual level*

**Child gender.** Gender was registered in a binary manner as male or female and reported by the teacher (see *Methods*). In regression analyses, boys were attributed a value of 0.5 and girls a value of -0.5.

**Child age at T1 (months).** The birth month and year at T1 were recorded by the teacher. As most children entered in first grade in September of the year of their sixth birthday, the "typical age in first grade" was defined as being between 69 months in September (= 6*12–3) and 80 months in September (= 6*12 + 8), both included. A few children, presenting with higher cognitive abilities at school, were allowed to drop a year and therefore were considered "one year ahead", with a younger age ranging between 57 and 68 months included. These higher abilities in language and math explained why these advanced children, though presenting with a lower age, performed better than older children (see SI **Figure S1**). Conversely, children with learning difficulties at school may repeat $1^{st}$ grade (though this is quite rare), or children may enter school late for other social reasons such as immigration; those children therefore were a year older than their peers in the class, with an age between 81 months and 92 months included. The variable "Age at T2" corresponded to "Age at T1" plus 4 months, and "Age at T3" corresponded to "Age at T1" plus 12 months.

**Child age category**. Using the child age at T1, we defined a 3-level categorical variable by subdividing the children into 3 groups based on their age: all children aged 57-68 months were categorized as "advanced"; all children aged between 69 and 80 months included were categorized as "typical age"; and all children aged 81-92 months were categorized as "late".

**Child family composition and child parental occupation.** Both variables were used in both Figure 5 and Extended Data Figure 6 analysis. Detailed information on individual children's socioeconomic background was obtained for the first cohort (2018-2019) by merging its data with a complementary data source (*Constats de rentrée en sixième*) completed by families when their children entered middle school (i.e., children entered $6^{th}$ grade in 2023), and therefore retropolated to grades 1 and 2. Two pieces of information were available: the relation of each legal representative (up to three) to the child, and their occupation and respective socio-professional category coded in 29 categories). Using the relation of each legal representative to the child, we categorized the family types as follow: families led by opposite-gender parents, same-gender parents, single mothers or single fathers, based on the child's legal representative declaration, and used it to describe the gender gap per family types as shown in Figure 5. Using the parental occupation and socio-professional category, we used the

individual socioeconomical status (SES) (*Indice de Position Sociale*[1]) of each legal representatives (details explained underneath) to build the family SES index and crossed it with each socio-progressional category (using the Ministry of Education cross-tabulations[2]) for the analyses in both Figure 5 and Extended Data Figure 6.

*Variables at the class level*

All the following class-level variables included the measures on typical-in-age children and were implemented on classes' sizes ranging between 6 to 27 children per class (see supplementary information (SI) Table S5). Note that the class-level variables' results in math presented in Extended Data Figure 4 could only be meaningfully computed by excluding mono-gender classes and classes that were too small to analyze the impact of gender proportions. Thus, analyses involving these variables were restricted to classes with at least 30% of boys and 30% of girls and were showed in Extended Data Figure 4.

**Class size.** Class size was defined as the number of children per class at T1. Some classes were declared with fewer than 5 children per class. This is a rare but possible situation, found mainly in rural areas where the paucity of children forces schools to gather all children belonging to primary school into a single class, called a "multi-level class", with an age range from 6 to 11 years old. Unfortunately, our database did not include information about whether a class was multi-level or not. As we were interested in the gender gap, which could only be meaningfully computed within a given class if that class comprised a sufficient number of children of either gender, when analyzing class-level variables, we only selected classes ranging from 6 to 27 children per class, in line with the referenced STAR experiment[53].

**Mean of class in math or language per gender.** From gaussianized variables, mean of boys and mean of girls in math were calculated for every selected class, then subtracted to obtain the gender gap per class at T1, T2 and T3, presented in the Extended Data Figure 4A.

**Mean of class in math or language.** From gaussianized variables, class mean in math and language per class were both calculated for every selected class. When used for the Extended Data Figure 4B, class mean in math was transformed into deciles, the lowest level being on the left of the x-axis and the highest level being on the right of the x-axis.

---

[1] The IPS (*Indice de Position Sociale*) is a continuous index built by the Department of Statistics at the Ministry of Education to describe children's socioeconomical background and described underneath.

[2] Original data, downloaded on : https://www.education.gouv.fr/media/158757/download, last update on April 23rd, 2025. Open data use license : Licence Ouverte / Open Licence version 2.0

**Heterogeneity in math or language per class at T1**. The initial heterogeneity of the class was computed as the standard deviation of the children's normalized and gaussianized math or language scores per class at T1. Only when used for the Extended Data Figure 4B, class heterogeneity in math was transformed into deciles, the lowest level being on the left of the x-axis and the highest level being on the right of the x-axis.

**Proportion of boys per class.** For every selected class, the proportion of boys was built as the number of boys divided by the total number of children and, ranged from 0 to 1.

**Gender of the first of class in math or language at T1**. Separately for language and for math, we identified the first of class and registered his or her gender. Boys were attributed a value of 0.5 and girls a value of -0.5. When several children were tied at the top of the class, their genders were averaged. For instance, if 2 boys and 1 girl were tied at the top of the class in math, the value for this variable was 0.1667, a positive score implying that a majority of boys were first of class in math in this class. The variable was numerical and ranged between -0.5 and 0.5. Then, we categorized the results into three groups: (1) Boys were first of class when the score was > 0, (2) girls were first of class when the score was < 0, (3) There was an equal number of boys and girls at the top of the class, i.e the score was equal to 0.

**Mean of class in math or language without the mean of first of class.** To visualize the gender gap effect in math of the class role model in math (i.e., a girl or a boy) in function of age, we firstly identified if the first of class in math was (1) a boy, (2) a girl or (3) mixed, then we excluded all firsts of class and computed the Cohen's d of the gender gap in math for the rest of the class, as presented in the Extended Data Figure 4C. To be coherent with all the other figures and models in this study, we focused the results of gender gaps presented on Extended Data Figure 4C only on typical-age children and belonging to classes with at least 30% of boys and 30% of girls as explained above. Cohen's d in math and language were both calculated from the gaussianized data for every selected class and among data where all math means of first of class were taken off.

*Variables at the school level*

**Type of School.** This variable was defined by the DEPP and the ministry of education using a combination of school status (private or public) and of four additional characteristics: the proportion of disadvantaged socio-professional categories in the geographic area surrounding the school; the proportion of students benefitting from social aid and scholarships in the living area surrounding the school; the proportion of children living in a sensitive urban area within the school; and the proportion of children attending the school who repeated a school year before their sixth grade. Thus, public schools were categorized in three tiers: Regular public schools, priority education (PE) public schools

and higher priority education (HPE) public schools. Private schools were considered as a fourth category, for a total of 4 categories. Note that being categorized as PE or HPE meant that the school was entitled to special educational benefits. In both categories, starting in 2018, more teachers were assigned such that class size could be reduced. The goal was to halve class size in those school districts. More precisely, in 2017, 2200 HPE classes were halved; in 2018, 3200 first-grade-PE classes and 1500 second-grade-HPE classes were halved; in 2019, 3900 second-grade-PE and -HPE classes were halved; and finally, both in 2020 and in 2021, all kindergarten, first and second-grade classes in PE and HPE had been halved. Outside the priority education system, a maximum of 24 children per class was mandatory for all regular public schools and kindergartens.

**School socioeconomic status (SES)**. This score, available for all cohorts at the school level, reflected the socioeconomic environment surrounding children. Computed by DEPP, it is based on a combination of the following data: parents' diploma level, material conditions level, family composition, cultural capital, cultural ambition, parental implication levels and cultural practices. All these data were registered in 6th grade only. Thus, a retrospective projection of every child's socioeconomical characteristics was implemented, by post-hoc attribution of the SES score to the primary school that a given 6th grader had attended. Finally, the school SES score was computed as the mean of all SES scores of children who attended the same primary school [54]. Ultimately, SES was a numerical variable, defined by the DEPP, going from ~ 50 to ~ 150 and representing school socioeconomic status, 50 being the lowest, and 150 the most advantageous.

**School pedagogy and private religious school information**. Both variables were created at the school level respectively using school names and type (only private schools can offer religious instruction in France), looking for explicit mentions of alternative pedagogies ("Montessori", "Freinet", etc.) and looking for specific religious-schooling usual words ("Saint" for Catholic schools) in the "Administrative information on the characteristics of schools" (APAE) data. Over all four cohorts, 8 % of children attended a private religious school, of which most children attended Catholic schools (i.e., a total of 230,482 children at the beginning of Grade 1), then Jewish schools (3,416 children) and very few Muslim schools (121 children). Regarding schools with alternative pedagogies, less than 1 % of children were classified as attending such schools. In detail, at the beginning of Grade 1, 2,312 children attended schools that we could ascertain as using the Freinet pedagogy, 775 children in schools using the Montessori pedagogy, and 83 children in a school using the Steiner-Waldorf pedagogy.

## Statistical analyses and supplementary results.

### Sensitivity analysis

All the analysis presented in this paper were performed on imputed data set, which therefore presented no missing data. To assess whether this decision impacted on our conclusions, we performed a sensitivity analysis by examining the average differences between the imputed dataset and the non-imputed one, using percent success variables, as gaussianization was not possible on the population with missing values (see SI Table S15). This analysis confirmed that no systematic differences existed between participants with missing data and those with complete data, especially regarding the results on gender gaps. More specifically, the final imputed population was compared to the non-imputed population which kept its missing values in the data frame. The final imputed population was also compared to the non-imputed population after removal of all children with some missing values. There were no significant differences between imputed population and non-imputed population with missing values, indicating a safe use of the imputed data in our analyses. There were, on the other hand, significant differences with the population that exhibited missing values – thus, analyzing only the latter would have exposed to a high risk of bias and non-representativeness.

### Gender gap effect size in Cohen's d

**Table 1** showed that gender gaps effects, measured in Cohen's d, were remarkably stable for math, language, problem-solving and number line both for T1, T2 and T3 and, in 2018, 2019, 2020 and 2021 and found for all socio-economical categories and ages. The results confirmed the rapid emergence of a gender gap favoring boys for number line and problem-solving assessments (positive values). In addition, from T1 to T2, gender gaps in math widened in favor of boys, with a similar magnitude in 2018 and in 2021 (i.e., difference T2-T1 $_{2018\text{-}2021}$ = 0.0002), whereas an almost similar but larger of ~ +0.01 Cohen's d difference magnitude in both 2019 and 2020. From T2 to T3, the gender gap in math in favor of boys was the largest in 2018, then dropped in 2019 (difference T3-T2 $_{2019\text{-}2018}$ ~ -0.07) and rose up again in 2020 and in 2021.

### Gender gap measures for each specific subtest in math and language, comparing boys and girls

To explore if the gender gaps identified in math and language were consistent in all subtests, we present the Cohen's d effects for each subtest as a function of gender, and for each cohort (2018, 2019, 2020 and 2021) (see **Table 2**). The results indicated that only a few subtests exhibited a reversal of the

gender gap effect. We can only offer here a few hypotheses about the origins of those reversals. Mental calculation was slightly superior in girls, perhaps because of its greater dependency on verbal automatisms [55]. Geometry was also slightly superior in girls, perhaps because it involved selecting among drawings and thus could be interpreted as a visual test that offered a break from all the other symbolic arithmetic tests [56]. Conversely, in speeded reading, boys tended to outperform girls, perhaps because they reacted better to the attentional challenge posed by speeded tests or because they reacted to what may look like a performance and competitive test when girls might exhibit more anxiety with time-limited exercises. Those reversed differences, however, were always small relative to the main gender gap effect reported in the main text.

**Main analysis: Multilevel multivariate mixed regression models at T3**

Multilevel multivariate mixed regression models were used to evaluate the association of gender and math scores at T3, after controlling for a large number of other variables (see **Table 3** and supplementary information (SI) **Table S16**). Similar regressions were performed on math scores at T1 (SI **Table S17**) and T2 (SI **Table S18**), as well as on language scores at T3 (**Extended Data Table 2**).

Language and math' individual levels at T1 (**Table 3**), as well as gender, presented with the highest predictive coefficients for math level at T3. SES score's coefficient was more than 10 times smaller than the three previous variables and, compared to the other years, SES score predictive coefficient was more important in 2019 (i.e., year with less school exposure due to Covid-19). Age had a very small positive influence on math at T3. First of class being a boy was associated with a small but higher math level at T3. The boys-girls ratio per class did not have any significant association with math at T3. A wider class heterogeneity of level in math at T1 was associated with a lower level in math at T3. In addition, we focused on interactions with gender to analyze which factors were associated with a gender gap raise or a diminution in math at T3: Significant gender-related effects are highlighted in bold. Higher math level at T1, SES score and first of class being a boy, were associated with a gender gap raise in favor of boys, whereas a higher language level, age and heterogeneity of level in class were associated with a smaller gender gap at T3. The model significance was estimated with the decrease of the model's deviance, showed in SI **Table S16**.

Furthermore, across all cohorts, we consistently observed a negative correlation between the random effects of gender and the random effect of initial math levels within each class. For instance, the coefficient for 2018 was -0.31, as detailed in **Table 3**. This finding suggested that math scores at T3 were less dependent on the initial math scores at T1 in classes with a strong gender effect. In simpler

terms, in classrooms where a noticeable bias favored boys over girls, with girls often achieving lower scores, the performance levels of both boys and girls at T3 showed a reduced dependency on their initial scores at T1.

The results obtained from our multilevel models were consistent and reproducible across multiple years, spanning 2019, 2020, and 2021, for math performance at T1, T2, and T3 (refer to SI **Table S17**, SI **Table S18** and **Table 3** respectively). However, it is important to note different associations when assessing 'Language at T3' as the outcome: results significantly differed from those in math proficiency for all cohorts (as shown in **Extended Data Table 2**).

**Additional results of modelling math at T1 using a multi-level regression model**

Most predictors for math at T1 were of the same significance, magnitude, and direction in 2018, 2019, 2020 and 2021 (see SI **Table S17**). The results indicated a massive effect of expected predictors such as age and SES score. More crucially for the aims of this paper, the gender gap in math at T1 was non-significant in 2018 ($\beta = -0.0044$ ($\pm$ 0.0050), NS), whereas it was small and in favor of boys in 2019, 2020 and 2021, in line with the introduction of the ministerial instruction of May 2019 to expose kindergarten children to formal math exercises as explained in the main text. The highly significant Gender * SES score interaction indicated, however, that children in higher SES classes were already affected by a gender effect. The negative effect of class size, at the beginning of the year, was somewhat surprising but might reflect a genuine early influence of class size on test results (see SI **Table S17**).

**Additional results of modelling math at T2 using a multi-level regression model**

As SI **Table S18** showed, for math at T2 the main effects related to gender were smaller, but very similar to those at T3: there was already a large and significant advantage for boys, which was larger for children with a higher level in math at T1 and in higher SES classes, and smaller for children with a higher level in language at T1. Contrary to T3, however, having a boy as first of class in math at T1 was not yet influential; in fact, it was slightly but significantly negatively correlated with the gender gap in favor of boys at T2 (only in 2018 and in 2021 but not in 2019 nor in 2020).

**Additional results of modelling language at T3 using a multi-level regression model**

In language, a negative effect of gender indicated that, everything else being equal, including language and math performance at T1, girls showed better performance than boys at T3 (see **Extended Data**

**Table 2**). The gender effect coefficient in language, however, was 10 times smaller than the gender effect on math (comparison between **Extended Data Table 2** and **Table 3**). Furthermore, variables such as initial level in language or in math, boys-girls ratio per class, or age did not contribute to significantly modulate the change in the gender gap in language from T1 to T3. The role model effect of having a boy as first of class in language did have a small influence in favor of boys in 3 out of 4 cohorts. A larger class size and a higher SES score also favored boys.

**Growth models**

As time was nested within students, themselves nested within classes, we implemented three-level growth models capturing the test scores in math as z-scores, where time was added as 0, 4 or 12 months, respectively corresponding to T1, T2 and T3. Regarding the Model 1 (see SI **Table S6**), it is quite understandable that the variable time had no effect (Estimate 0.0000, NS (0.952)), as the mean score in math, once expressed as a z-score of the entire population, did not evolve much with time. However, the interaction between time and gender was highly significant in this model (Estimate = 0.0198, $p < 0.0001$), indicating that the gender gap widened with time in school.

We also implemented a more complete multilevel model, Model 2 (see SI **Table S6**) including all variables assessed in the main regression model of **Table 3** (except for Language at T1, as all girls and boys were not at an equal level in language). In addition, we assessed interactions between time and gender. Even with the inclusion of the later variables, we noted similar results regarding the interaction of time and gender in math compared to the previous model.

All growth models' results confirmed our previous results and converged towards a significant interaction between time and the gender effect ($p < 0.0001$): As time progressed, a difference between boys and girls emerged (i.e., the time × gender interaction was positive and significant, indicating a growth of gender gaps with time. This interaction was modulated by several triple interactions: both the proportion of boys per class, and a higher socioeconomical status, increased the interaction between time and the gender gap.

**Bayes Factor for testing null hypothesis**

To test any of the null effects we found were strong and interpretable (vs. lack of evidence), we performed Bayesian statistics on our data (there were very few places in our manuscript where we reported null effects). The Bayes Factor was computerized from the function *generalTestBF*() allowing to include our full model with both continuous and categorial variables as well as their interaction, in

the package *BayesFactor* in the R software. To obtain the Bayes Factor of each variable involved in the general model, omit one specific factor at a time, we used the option "whichModels = top" that tested all models that could be created by removing or leaving in a main effect or interaction term from the full model. Then, as the denominator contained the specific factor, we applied the "1/result" to get the final Bayes Factor corresponding to each main effect or interaction term of the model. One important null effect that we report is the absence of a math gender gap at school entry in the first cohort. SI **Table S19** reports the Bayes factors for each comparison of a Beta weight with zero mentioned in SI **Table S17** (Multilevel model of Math at T1 with a null effect for gender). Both models converged towards the same conclusions. The Bayes Factor associated with the absence of a math gender gap at T1 is 0.004, i.e. "extreme evidence" for the null hypothesis.

In addition, we have attempted to add Bayes Factors (BF) for our **Table 3**, which reports a multilevel model of Math at T3. However, we faced difficulties to find a function that could compute the Bayes Factors for our huge and multilevel model, comprising two random-effect factors (i.e., Math at T1 and Gender). Instead, in **Table S19**, we report our use of the BayesFactor package in R software, using a linear regression model that included all fixed-effect variables and their interactions as seen in **Table 3**, but no random effects. For most effects and interactions, the results are highly convergent: a significant finding is always accompanied by a huge BF, indicating "extreme evidence for H1"; and conversely, non-significant findings are accompanied by a very low BF, indicating "extreme evidence for H0". There were a few exceptions however (though not directly relevant to our conclusions), for instance the main effect of "class size" has a modest but significant impact in **Table 3** (beta = 0.0095), while its BF=0.24 indicates moderate evidence for the null hypothesis. We believe that this small discrepancy is due to the absence of the two random effects in the Bayes Factor model that we were running. In summary, all Bayes Factor results for the most important null effects in our study were consistent with our conclusions.

**Gender gap for individual math subtests and relation to test periods and test difficulty (Table S7)**

In Table S7, we provide subtest-level analyses of the gender gap. For each math subtest (see Table S4 for the list) and each cohort, we computed the standardized gender gap at a given subtest, and correlated it with subtest difficulty (measured as one minus the average score at the subtest divided by the max possible score). The corresponding plot appears in Extended Data Figure 3. The raw correlation between the two is large (rho=0.41). Note that the subtests varied across T1, T2 and T3 in order to track children's progress. It is therefore important to decorrelate the two variables of test timing and test difficulty. As shown in Table S7, when controlling for the timing of the test (T1 vs T2 vs T3), there is still a large association between subtest difficulty and the gender gap. This association is robust to controlling for cohort dummies and the type of subtest (the different rows on the top panel

of Table S7) or cohort dummies and fixed effects for each specific subtest (i.e. one effect for each type of subtest and timing of the subtest). In the latter case, the relationship between subtest difficulty and the gender gap is solely identified from variations across cohorts in the difficulty of a given subtest at a given point in time.

Crucially, we also check whether the differences in the gender gap between T1, T2 and T3 could be explained only by the increase in test difficulty. This is barely the case: for example, the average difference in the gender gap within a given type of subtest between T1 and T3 moves from 0.194SD without the test difficulty variable to 0.179SD when controlling for test difficulty. Hence, the emergence of the gender gap between T1 and T2 or T3 cannot be attributed to an increase in test difficulty only.

**Additional analyses and figures at the class level (Extended Data Figure 4)**

As this study measured the gender gap in various tests at school, and as it included both individual and class-level variables, we had the opportunity to test a number of class-level effects on the gender gap in math using Cohen's d (see Extended Data Figure 4).

A subtlety of this analysis concerns the age of the children. To be most representative of the true content of each classroom, we first defined all class-level variables while including all children, regardless of their age (i.e., including advanced-, typical-, and late-in-age children). However, like in the rest of the text, we examined the effects of those variables on children of typical age in first grade (i.e., 69- to 80-year-old children), and therefore did not include the extreme-aged children (i.e., advance-in-age or late-in-age children) (see Extended Data Figure 4). Therefore, our results were representative of the class-variables effects on gender gaps among children of typical age in first grade. The results were, however, virtually identical when we did not follow this strict procedure.

**Extended Data Figure 4A** shows the distribution, over classrooms, of the within-class average math gender gap, expressed as the difference in z score between boys and girls. Note that for this plot, in order to calculate a class-level measure of the gender gap, we had to restrict the analysis to classes with enough children of either gender. We therefore restricted our analysis to classes at least 30% of boys and 30% of girls per class. Selections were indicated as "Step 5" in the SI Table S5 and were applied for data management in 2018, 2019, 2020 and 2021. As can be seen in Extended Data Figure 4A, the distribution was centered on zero at T1, but many classrooms showed a bias (i.e., a shift in favor of boys) at T2 and a more pronounced bias in favor of boys at T3. Results were similar for 2018, 2019, 2020 and 2021.

**Extended Data Figure 4B** plots the effect, on the class-level gender gap, of several variables: (1) class size, (2) class initial level in Math, (3) heterogeneity of level in math in the class, (4) boys-girls ratio per class. Effect sizes are shown as Cohen's d. It can be seen that class size and initial level tended to increase the gender gap, while class heterogeneity decreased it, and the proportion of boys had very little or no effect. Those effects were rather stable in 2018, 2019, 2020 and 2021 (see **Table 3** for significance).

Finally, in **Extended Data Figure 4C**, we plotted the impact of the role model in the class in math on the gender gap in the class. For this latter, the graph was divided into two: the graph on the left represents classes where a boy was first of class in math at T1, while the graph on the right represents classes where a girl was first of class in math at T1. In both graphs, we recomputed the Cohen's d measuring the gender gap in math after removal of the first of class, to visualize the impact the role model had on the rest of the class's gender gap in math. There is a subtle point here: removal of the data from the first-of-class at T1 was necessary in order to avoid some degree of circularity in the analysis, (since this data was used for sorting the classes), but this procedure had a subtle effect of biasing the data in favor of girls. This is because this procedure implied removing more boys than girls, as even at T1, boys are more frequent at either end of the distribution (see **Figure 1C**). As a result of removing more "top-boys" than "top-girls", the remaining data became biased in the other direction, and a small advantage in favor of girls can be seen at T1 in Extended Data Figure 4C. However, as in all previous plots, even with this artefactual bias, the data quickly reverses in favor of boys at T2 and T3. Furthermore, the figure shows that having a girl or a boy as the first of class in math at T1 had a small but significant impact on the gender gap in math at T3 (see **Table 3**): in classes with a boy as a role model in math, boys' advantage in math grew slightly more from T1 to T3.

**Matching and reweighting (Extended Data Table 1, Table S14, Extended Data Figure 7)**

The matching analysis, by nature, focused on a subset of matched pairs, thus running the risk of being unrepresentative of the cohort (in particular, both scenarios led to an over-representation of children in the top results). To further confirm the results, we also implemented six additional reweighting techniques (see **Extended Data Table 1**): Average weighting using G-computation, propensity weighted regression, inverse propensity weighting (IPW), doubly-robust estimation (AIPW) with various nuisance components estimation techniques such as Ordinary Least Square (OLS), logistic regression (logit), random forest approaches and target maximum likelihood estimation (TMLE)). While reweighting the two groups makes them more comparable, it is obviously impossible to randomize the attribution of gender, and those analyses therefore remain correlational, not causal

(although the term "causal inference methods" is often used, inappropriately, in the literature). What they do afford is a careful ponderation and attempted cancellation of the contributions of any other potential nuisance variables. Furthermore, compared to matching, the latter techniques present the advantage that no data is dropped. Finally, within the several nuisance components estimation techniques, the random-forests approaches cancel the parametric assumptions inherent to logistic regression or OLS, and are supposed to be more reliable, at least in large samples (which is the case in the present study).

The same covariates than those used for matching (see SI **Table S14**) were used for adjustment, only the statistical methodology changed. Intuitively, those approaches allowed all models to decide how to best capture any initial imbalance in the so-called nuisance functions, either by weighing the data differentially, or by relying on an outcome model to infer the average effect of being a girl or a boy, all other characteristics remaining equal. The first four methods used parametric nuisance functions, while random forest and TMLE relied on a non-parametric approach.
Results are presented in **Extended Data Table 1.** No matter which causal inference techniques was implemented, all results tended towards same conclusions: a large emerging gender gap (i.e., 0.28 to 0.30 in z-score) favoring boys in math at T3.

**Comparing gender gaps' magnitudes between 2018, 2019, 2020 and 2021 (Extended Data Table 3)**

To test for variations in the gender gap in math between T2 and T3 across the different cohort years, we implemented three models, each comparing one year with the next year. The T3-T2 difference in z score was modeled as a function of gender, year and their interaction. The results indicated that the T3-T2 gender gap was large in 2018, dropped in 2019 ($\beta_{gender*year}$ = -0.0685 (0.0027) ***) and rose up again in 2020 and 2021 (see **Extended Data Table 3**).

**Comparison of children with similar age but one-year difference in schooling exposure (Table S8)**

Because of the institutional cutoff on school entry, children born in December of a given year had been enrolled in school for a whole year when they took the T3 test, while, at the same moment, children born in January of the following year were only taking the school-entry T1 test. Even though these children were born only one month apart and were tested simultaneously, they differed in amount of schooling by a full year. Comparing their T1 and T3 scores again revealed a large and significant gender gap in the schooled group, thus differing significantly from the unschooled group. These comparisons

are reported in **Table S8**, first for the overall math test scores at T1 and T3, then restricted to the problem solving and number line tests.

**Comparison of children born a few months apart, yet schooled on different years and therefore tested at different ages (Table S9)**

As a control, **Table S9** shows the characteristics of children born a few months apart (from October of a given year to March of the next year) (see **Table S9**). This table shows, for each birth month and year, the number of children, and the proportion of girls. There were no discontinuities in these parameters and, most importantly, little or no gender gap effect when comparing children born in December of a given year and those born in January of the following year. In each group of children born on a given month and year, we evaluated the impact of gender with a linear or logistic regression. When the dependent variable was SES or type of school (Private vs. Public schools; Public and private vs. PE and HEP schools), no gender gap was observed. When the dependent variable was math scores at T1, T2 or T3, in each group we again observed that the gender gap increased strongly from T1 to T2 and T3, but the gap itself showed little or no consistent difference between children born in December and those born in January the next year (lines marked “delta” in Table S8). As discussed in the main text, the only difference of some size was between the cohorts 2018 and 2019 (line marked “Delta Dec 12 – Jan 13), most likely because in May 2019, prior to T1 acquisition for the 2019 cohort (September 2019), the French education Ministry issued a formal request to kindergarten teachers, asking them to prepare children for the coming first-grade national assessments by introducing more formal training in math and language.

In summary, the math gender gap emerges in a systematic manner with schooling: it exhibits a monotonic growth from T1 to T2 and T3, yet shows very little influence of birth month and year.

**Regression Discontinuity Design (RDD)**

Prior to RDD, each cohort was merged with the next cohort to obtain continuity in birth dates around January 1st. The **SI Figure S1** showed the distribution of birth dates for children born around January 1st and taking math tests, for all cohorts from 2018 to 2021. The distribution of birth dates revealed some very local proportion fluctuations. There are fewer births during weekends, and, to some extent, on January 1st. This is probably because some births (e.g. caesarean) are programmed a few days in advance on weekdays or outside holidays.

The estimation of RDD models relies on equations of the type:

$$S_{iTj} = P(D_i) + \beta 1_{D_i > 1st\ Jan\ 2013} + Q(D_i) 1_{D_i > 1st\ Jan\ 2013} + \epsilon_{iTj}$$

where $S_{iTj}$ is the test score (normalized as a z-score) of children *i* at test $T_j$, ;$D_i$ is the date of birth of children *i* (provided as an integer number and normalized to 0 at a certain date) ; $P(D_i)$ and $Q(D_i)$ are polynomials in $D_i$ ; $1_{D_i > 1st\ Jan\ 2013}$ is an indicator variable equal to 1 if children *i* was born after the 1st of January 2013 ; and $\epsilon_{iTj}$ is an error term. $\beta$ is the coefficient of interest: it captures the effect on test score of being born just after January 1st, 2013, versus just before. We estimated variants of this equation with first-order polynomials on local bandwidths around the cut-off date using the *rdrobust* package in STATA 18 software. We used a standard triangular kernel attributing greater weight to the observations closer to the cut-off date. We provide as a robustness check estimates obtained after excluding the children that were very close to the cut-off (born between December 29th and January 3rd). This "donut-hole approach" ensures that RDD estimates do not capture the very local (and last-minute) targeting of births that may be anticipated outside weekdays and holidays.

**SI Figure S2** provided graphical evidence evaluating for discontinuities as a function of age at T1 on the T1 test scores at the first available cut-off (1st January 2013) for girls and boys separately. This plot showed a broad 2-year window on birth date. The top panels of **SI Figure S2-A** showed age at test, making it clear that (i) obviously, the later they are born, the younger children are when taking T1, but (ii) this is no longer true when they cross the 1st January threshold, as they become one year older at T1. This effect of the date of birth on age at T1 was very similar for girls and boys.

A formal RDD regression confirmed that both girls and boys born on January 1st 2013 are on average a year older when they reach test time T1 (364.7 days for boys versus 364.8 days for girls, see **Table S10**) than those born on December 31st. Results were similar for T2 and T3 (**Table S10**). The bottom graphs of **SI Figure S2-B** then showed T1 scores in the same format, indicating that (i) the later they are born, the worse children perform at T1, (ii) this is not true when they cross the January 1st 2013 threshold as the performance increase by about 0.7 SD. Crucially, the effect of the date of birth on performance at T1 appeared comparable for girls and boys. The comparison of the effect of crossing the threshold on

age and on test score made it possible to conclude that the local average treatment effect (for children born around January 1$^{st}$, 2013) of being one year older when taking a test is around 0.7 SD. This effect also captured the related effects of starting school later (for T2 and T3 in particular) and of being the oldest in one's cohort.

As a formal test of this conclusion, and to offer complete comparisons of the effect of age on girls' and boys' performance on the three test points, we provided in **Table S11** all RDD estimates of being born around January 1$^{st}$ of 2013, 2014 or 2015 on test scores at T1, T2 or T3, for girls and boys separately, for girls and boys separately, as well as formal tests of the difference in estimates between girls and boys. While the age effect on test scores was very large (around 0.7 SD at T1, 0.6 SD at T2 and 0.55 SD at T3), the difference in this effect between girls and boys remained limited (always smaller than 0.10 SD) and was not always statistically significant at conventional levels, showing that age (and the related factors detailed above) cannot entirely drive the emergence of the gender gap. **Table S12** provided similar results with the donut-hole approach and confirmed this conclusion.

We provided several tests to back up our conclusions. First, we performed formal balancing checks using the RDD approach for one school-level variable (average school SES) and one class-level variable (share of boys in class in Grade 1). These tests, provided in **Table S13,** indicate that being enrolled in primary school one year later due to the institutional cutoff on January 1st had limited impact on these characteristics (only a few estimates are significant at conventional levels, which is expected given the number of tests provided). Second, we performed tests of manipulation in the running variable at each cutoff based on the local polynomial density estimators proposed in Cattaneo, Jansson and Ma (2020)[57]. The p-values from these tests are provided in last column of **Table S13** and reveal some sign of manipulation with the 2013 cutoff but not with the 2014 and 2015 cutoffs. This implies that we may want to consider the results for 2013 cautiously and focus on other cutoffs at which we actually found a much smaller difference between girls and boys (always smaller than 0.05 SD in Table S10). Third, we replicated Tables S14 and S15 using a fixed, smaller bandwidth of 45 days on each side of each cutoff. Results (available upon request) are very similar.

**Supplementary methods bibliography**


51. Brankaer, C., Ghesquière, P. & De Smedt, B. Symbolic magnitude processing in elementary school children: A group administered paper-and-pencil measure (SYMP Test). *Behav. Res. Methods* 49, 1361–1373 (2017).

52. Dehaene, S., Izard, V., Pica, P. & Spelke, E. Core knowledge of geometry in an Amazonian indigene group. *Science* 311, 381–384 (2006).

53. Angrist, J. & Lavy, V. Using Maimonides' Rule To Estimate The Effect Of Class Size On Scholastic Achievement. *Q. J. Econ.* 114, 533–575 (1999).

54. Rocher, T. Construction d'un indice de position sociale des élèves. *Educ. Form.* 5–27 (2016).

55. Dehaene, S. & Cohen, L. Cerebral pathways for calculation: double dissociation between rote verbal and quantitative knowledge of arithmetic. *Cortex J. Devoted Study Nerv. Syst. Behav.* 33, 219–250 (1997).

56. Huguet, P., Brunot, S. & Monteil, J. M. Geometry versus drawing: Changing the meaning of the task as a means to change performance. *Soc. Psychol. Educ. Int. J.* 4, 219–234 (2001).

57. Cattaneo, M. D., Jansson, M., & Ma, X. (2020). Simple local polynomial density estimators. Journal of the American Statistical Association, 115(531), 1449-1455.

## Supplementary Tables

**Supplementary Table S4.** Summary of cognitive tests evaluated at each period.

| Domain | Skill area | Cognitive skills evaluated | Time of evaluation | | |
|---|---|---|---|---|---|
| ***Math skills*** | | | | | |
| | Number reading | Converting an Arabic numeral into a spoken number word | T1 | | T3 |
| | Number writing | Converting a spoken number word into an Arabic numeral | T1 | T2 | T3 |
| | Enumerating | Counting an organized or disorganized collection and identifying the Arabic numeral corresponding to that quantity. | T1 | | T3 |
| | Number comparison | Selecting the larger of two Arabic numerals | T1 | T2 | |
| | Problem solving | Understanding an orally presented arithmetic problem, choosing the correct operation, and finding the exact result. | T1 | T2 | T3 |
| | Number line | Finding the number corresponding to a given position on the number line. | T1 | T2 | T3 |
| | Addition and subtraction | Solving simple written addition and subtraction problems. | | T2 | T3 |
| | Mental calculation | Mentally calculating additions of two spoken numbers. | | | T3 |
| | Geometry | Recognizing and using concepts of alignment, right angle, length, and symmetry. | (T1) | | T3 |
| ***Language skills*** | | | | | |
| | Oral comprehension of words | Understanding a word read aloud by the teacher and finding the corresponding picture. | T1 | | T3 |
| | Oral comprehension of sentences | Understanding a sentence read aloud by the teacher and finding the corresponding picture. | T1 | T2 | T3 |
| | Oral comprehension of texts | Understanding a text read aloud by the teacher and finding the corresponding picture. | T1 | | |
| | Phoneme manipulation (or phoneme awareness) | Identifying the word that begins or ends with the same phoneme as a target word | T1 | T2 | |
| | Syllable manipulation | Identifying words that begin or end with the same syllable as a target word. At T2 and T3, writing a spoken syllable to dictation (mu, ti, na, lur, sar, ol, moi, che,…). | T1 | T2 | T3 |
| | Letter-sound association | Identifying the initial phoneme of a spoken monosyllabic word and associating it with the corresponding letter. | T1 | T2 | |
| | Letter recognition | Recognizing the different writings of a letter read aloud by the teacher. | T1 | | |
| | Visuo-attentional abilities | Comparing two consonant strings, in a limited time (2 minutes). | T1 | | |
| | One-minute word reading | Reading correctly as many words as possible among a list of words, in a limited time (1 minute). | | T2 | T3 |
| | One-minute text reading | Reading correctly as many words as possible in a text, in a limited time (1 minute). | | T2 | T3 |
| | Writing words to dictation | Writing the correct spelling of a spoken word (silent letters at the end of the word accepted). | | T2 | T3 |
| | Reading comprehension of sentences | Understanding a sentence and circling the corresponding picture. | | (T2) | T3 |
| | Reading comprehension of texts | Understanding a short text and answering questions read by the teacher. | | | T3 |

Note: A period indicated between parentheses meant that the assessment was added after 2018.

**Supplementary Table S5.** Overview of missing values in the four cohorts and their data management.

| Cohorts | 2018 | 2019 | 2020 | 2021 |
|---|---|---|---|---|
| **Initial number of children in the database**<br>(n = 2 871 080) | 610,905 | 711,452 | 743,734 | 804,989 |
| **Overall proportion of missing values (%)** | 1.27 | 1.18 | 1.06 | 4.30 |
| **Overall proportion of children with at least 1 missing value (%)** | 20.12<br>N = 122,922 | 19.76<br>N = 140,590 | 17.37<br>N = 129,157 | 25.58<br>N = 205,992 |
| **Step 1: Number of remaining children after removal of those who were absent from all 3 sessions (T1, T2, T3)**<br>(n = 2 869 554) | 610,830<br>N classes : 43,970<br>N classes > 27 : 157<br>N classes < 6 : 2676<br>N schools : 27,043 | 711,351<br>N classes : 51,599<br>N classes > 27 = 106<br>N classes < 6 = 2880<br>N schools : 30,578 | 743,606<br>N classes : 54,073<br>N classes > 27 = 93<br>N classes < 6 = 2920<br>N schools : 31,515 | 803,767<br>N classes : 54,224<br>N classes > 27 = 264<br>N classes < 6 = 2341<br>N schools : 31,772 |
| **Number of children as a function of age (in months)** | Young (51-68) : 3,719<br>Typical (69-80) : 592,779<br>Late (81-98) : 14,219 | Young (51-68) : 4,426<br>Typical (69-80) : 689,833<br>Late (81-98) : 16,848 | Young (51-68) : 4,213<br>Typical (69-80) : 720,670<br>Late (81-98) : 18,534 | Young (51-68) : 5,240<br>Typical (69-80) : 773,423<br>Late (81-98) : 25,880 |
| **Step 2: Number of remaining children after removing classes where gender was missing and imputation of missing data**<br>(n = 2 869 344) | 610,785<br>N classes with gender missing: 3<br>N students : 45 | 711,316<br>N classes with gender missing: 3<br>N students : 35 | 743,476<br>N classes with gender missing: 7<br>N students : 56 | 803,767<br>N classes with gender missing: 0<br>N students : 0 |
| **Gender** | Boys : 310,644<br>Girls : 300,141 | Boys : 361,745<br>Girls : 349,571 | Boys : 376,760<br>Girls : 366,716 | Boys : 410,700<br>Girls : 393,067 |
| **Step 3: Selecting children in classes containing 6 to 27 children per class (removing extreme size classes)**<br>(n = 2 739 815)<br><br>At this stage we gaussianized the test scores and computed the following class-level variables:<br>- boys' proportion per class<br>- gender of first of class<br>- Heterogeneity of level in math and language per class | 586,949<br><br>N classes : 39,573 | 686,138<br><br>N classes : 46,671 | 717,326<br><br>N classes : 49,010 | 749,402<br><br>N classes : 49,703 |
| **Step 4: Selecting children of typical age (69 to 80 months) at T1**<br>(n = 2 653 082)<br>Subject-level regression models of the gender gap were performed on these data | 569,771 | 665,632 | 695,449 | 722,230 |
| **Step 5: Selecting classes with at least 30% of boys and 30% of girls**<br>(n = 2 455 483)<br>Class-level evaluations of the gender gap were performed on these data. | 526,556<br><br>N = 43,215 children belonging to classes with an excess of boys or an excess of girls | 614,264<br><br>N = 51,368 children belonging to classes with an excess of boys or an excess of girls | 642,870<br><br>N = 52,579 children belonging to classes with an excess of boys or an excess of girls | 671,793<br><br>N = 50,437 children belonging to classes with an excess of boys or an excess of girls |

Note that the number of children in the database increased as test acceptance improved between 2018 and 2021.

**Supplementary Table S6.** Modeling the data using a growth framework, with time nested in students, nested in classes in 2018.

| | Model 1: Math | | Model 2: Math | |
|---|---|---|---|---|
| **Fixed effects** | **Estimate (Std. Error)** | **Pr(>\|t\|)** | **Estimate (Std. Error)** | **Pr(>\|t\|)** |
| *Intercept* | 0.0177 (0.0024) | *< 0.0001* | *-0.1427 (0.0013)* | *< 0.0001* |
| Time | 0.0000 (0.0002) | NS (0.952) | 0.0029 (0.0011) | 0.0075 |
| Age at T1 | 0.1986 (0.0012) | < 0.0001 | 0.1961 (0.0012) | < 0.0001 |
| Gender | -0.0015 (0.0023) | NS (0.502) | -0.1280 (0.0014) | < 0.0001 |
| First of class is a boy in Math at T1 | - | - | 0.0210 (0.0045) | < 0.0001 |
| SES | - | - | 0.1412 (0.0001) | < 0.0001 |
| Class size | - | - | -0.0777 (0.0023) | < 0.0001 |
| Boy proportion in class | - | - | -0.0110 (0.0021) | < 0.0001 |
| Heterogeneity of level in math at T1 | - | - | 0.0029 (0.0021) | NS (0.1556) |
| Gender * Age | -0.0002 (0.0023) | NS (0.707) | -0.0002 (0.0025) | NS (0.9295) |
| **Time * Gender** | **0.0198 (0.0002)** | **< 0.0001** | **0.0148 (0.0011)** | **< 0.0001** |
| Time * Age | -0.0027 (0.0000) | < 0.0001 | -0.0026 (0.0001) | < 0.0001 |
| Time * First Boy | - | - | 0.0010 (0.0003) | 0.0050 |
| Time * SES | - | - | -0.0000 (0.0000) | 0.0141 |
| Time * Class size | - | - | 0.0021 (0.0002) | < 0.0001 |
| Time * Boy Proportion | | | 0.0003 (0.0002) | NS (0.0570) |
| Time * Heterogeneity | - | - | -0.0048 (0.0002) | < 0.0001 |
| Gender * First Boy | - | - | 0.3734 (0.0047) | < 0.0001 |
| Gender * SES | - | - | 0.0010 (0.0001) | < 0.0001 |
| Gender * Class size | - | - | -0.0113 (0.0025) | < 0.0001 |
| Gender * Boy proportion | - | - | -0.0433 (0.0024) | < 0.0001 |
| Gender * Heterogeneity | - | - | -0.0071 (0.0022) | 0.0016 |
| Time * Gender * Age | - | - | -0.0002 (0.0002) | NS (0.2207) |
| Time * Gender * First Boy | - | - | -0.0108 (0.0004) | < 0.0001 |
| Time * Gender * SES | - | - | 0.0000 (0.0000) | < 0.0001 |
| Time * Gender * Class size | - | - | 0.0004 (0.0002) | 0.0198 |
| Time * Gender * Boy proportion | | | 0.0014 (0.0002) | < 0.0001 |
| Time * Gender * Heterogeneity | - | - | 0.0000 (0.0002) | NS (0.7504) |
| **Random effects** | | | | |
| Student level – Intercept | 0.4644 | | 0.4580 | |
| Student level – Time | 0.0000 | | 0.0000 | |
| Class level – Intercept | 0.1824 | | 0.1278 | |
| Class level – Gender | 0.0096 | | 0.0008 | |
| Class level – Time | 0.0010 | | 0.0009 | |
| Class level – Intercept * Gender | **0.33** | | **0.53** | |
| Class level – Intercept * Time | **-0.51** | | **-0.63** | |
| Class level – Time * Gender | **0.13** | | **0.26** | |
| Residual | 0.3057 | | 0.3054 | |

Note: The formula implemented were as follow: ***Model 1**: math ~ time + Age at T1 + Gender + Gender*time + Gender*Age + (1 + time | Student level) + (1 + time + Gender | Class level);* ***Model 2**: math ~ time + Age at T1 + Gender + [Class variables] + [interaction with Time] + [interaction with Gender] + [interaction with Time and Gender]+ (1 + time | Student level) + (1 + time + Gender | Class level)*

**Supplementary Table S7.** Relationship between Math subtest level, gender gaps, test periods and test difficulty (math subtests only)

| | **Regression models<br>Dependent variable: gender gap (boys minus girls) at subtest (standardized)** | | | | | |
|---|---|---|---|---|---|---|
| | (1) | (2) | (3) | (4) | (5) | (6) |
| **Subtest difficulty (standard error)** | | 0.0621 *** (0.0120) | 0.0540 *** (0.00989) | | 0.0739 *** (0.0096) | 0.0540 *** (0.0106) |
| **T1** | Ref | | Ref | Ref | Ref | |
| **T2** | 0.0647** (0.0251) | | 0.0633 ***(0.0213) | 0.0449*** (0.0117) | 0.0795 *** (0.0097) | |
| **T3** | 0.1410 *** (0.0235) | | 0.1270 *** (0.0201) | 0.1940 *** (0.0117) | 0.1790 *** (0.0088) | |
| ***Controls entered in the model :*** | | | | | | |
| **Cohort dummies** | Yes | Yes | Yes | Yes | Yes | Yes |
| **Subtest dummies** | No | No | No | Yes | Yes | No |
| **Subtest X (test period) dummies** | No | No | No | No | No | Yes |
| **Observations** | 80 | 80 | 80 | 80 | 80 | 80 |
| **R-squared** | 0.332 | 0.262 | 0.525 | 0.899 | 0.948 | 0.986 |

Note: For math subtests only, general linear models were used to evaluate how the gender gap for each subtest (e.g. subtracting, adding, problem solving, etc.) varies with subtest difficulty and test period. The 80 data points include the 4 consecutive cohorts (starting Grade 1 in 2018, 2019, 2020 or 2021) and the 20 tests pooled across the three-testing period (T1, T2 or T3). Six linear models are considered (columns), and the corresponding regression slopes and their standard errors are reported. Indicator variables for cohorts are systematically included. Column 5 controls further for subtest types of fixed effects (one effect for each type of test, no matter the test period) and column 6 for subtest X test period fixed effects (one effect per type of test and test period). Standard errors in parentheses. *** p<0.01, ** p<0.05, * p<0.1

**Supplementary Table S8.** Comparison of the magnitude of gender gaps for children born one-month apart. i.e. December of year *n* (entering grade 2 and taking T3 tests), versus January of year *n*+1 (entering grade 1 and taking T1 tests). These children are very similar in age, and are tested at the same time, yet differ by a full year of schooling.

| **Difference between schooled and unschooled children born 1 month apart** | **Math** | **Problem solving** | **Number line** |
|---|---|---|---|
| **Born December 2012 (T3 tests) vs. Born January 2013 (T1 tests)** | 0.2039 (0.0121) *** | 0.1480 (0.0123) *** | 0.1859 (0.0123) *** |
| **Born December 2013 (T3 tests) vs. Born January 2014 (T1 tests)** | 0.1826 (0.0114) *** | 0.1199 (0.0116) *** | 0.1711 (0.0116) *** |
| **Born December 2014 (T3 tests) vs. Born January 2015 (T1 tests)** | 0.1975 (0.0113) *** | 0.1389 (0.0115) *** | 0.1967 (0.0115) *** |

**Note** : t tests comparing the cells in Bold in Table S9 ; NS = Non significant ; * $p < 0.05$ ; ** $p < 0.01$ ; *** $p < 0.001$.

**Supplementary Table S9.** Description of children born few months apart but with 1 school year of difference, between 2018 and 2019, 2019 and 2020 and 2020 and 2021.

| ***Description*** | | | | | | | | ***Gender gaps (effect size, SE, and significance) on*** | | | | | |
|---|---|---|---|---|---|---|---|---|---|---|---|---|---|
| **Born in** | **Cohort** | N | N Girls | % girls | Age at T1 | Age at T2 | Age at T3 | **SES score** | **Private vs. Public school** | **Pub/private vs. REP/REP+ schools** | **Math score at T1** | **Math score at T2** | **Math score at T3** |
| | | | | | | | | | | | | | |
| October 2012 | 2018 | 50123 | 24973 | 49,8 | 71 | 75 | 83 | -0,0122 (0,0089) NS | -0.0344 (0.0295) NS | 0.0785 (0.0243) ** | -0,0021 (0,0087) NS | 0,0788 (0,0088) *** | 0,2466 (0,0086) *** |
| November 2012 | 2018 | 46499 | 22984 | 49,4 | 70 | 74 | 82 | 0,0178 (0,0092) NS | 0.0284 (0.0308) NS | -0.0125 (0.0251) NS | -0,0010 (0,0090) NS | 0,0867 (0,0091) *** | 0,2647 (0,0089) *** |
| **December 2012** | 2018 | 46903 | 23486 | 50,1 | 69 | 73 | 81 | 0,0097 (0,0092) NS | -0.0150 (0.0303) NS | -0.0088 (0.0250) NS | -0,0013 (0,0089) NS | 0,0774 (0,0090) *** | **0,2489 (0,0086) ***** |
| **January 2013** | 2019 | 54779 | 26965 | 49,2 | 80 | 84 | 92 | 0,0004 (0,0085) NS | -0.0077 (0.0271) NS | -0.0271 (0.0224) NS | **0,0451 (0,0084) ***** | 0,1285 (0,0083) *** | 0,2382 (0,0083) *** |
| February 2013 | 2019 | 49589 | 24565 | 49,5 | 79 | 83 | 91 | 0,0010 (0,0090) NS | -0.0451 (0.0280) NS | 0.0326 (0.0238) NS | 0,0254 (0,0088) ** | 0,0960 (0,0088) *** | 0,2270 (0,0088) *** |
| March 2013 | 2019 | 53795 | 26511 | 49,3 | 78 | 82 | 90 | 0,0125 (0,0087) NS | -0.0431 (0.0271) NS | -0.0182 (0.0228) NS | 0,0366 (0,0085) *** | 0,1183 (0,0085) *** | 0,2288 (0,0085) *** |
| **Delta (Dec 12 - Jan 13)** | | 7876 | 3479 | 0,9 | 11 | 11 | 11 | 0.0094 (0.0126) NS | -0.0073 (0.0406) NS | 0.0183 (0.0335) NS | -0.0463 (0.0122) *** | -0.0510 (0.0122) *** | 0.0107 (0.0121) NS |
| | | | | | | | | | | | | | |
| October 2013 | 2019 | 57743 | 28591 | 49,5 | 71 | 75 | 83 | 0.0027 (0.0083) NS | -0.0156 (0.0263) NS | -0.0054 (0.0222) NS | 0.0315 (0.0081) *** | 0.1229 (0.0081) *** | 0.2199 (0.0080) *** |
| November 2013 | 2019 | 54475 | 26929 | 49,4 | 70 | 74 | 82 | 0.0179 (0.0086) * | -0.0528 (0.0273) NS | -0.0151 (0.0228) NS | 0.0288 (0.0083) *** | 0.1339 (0.0083) *** | 0.2363 (0.0082) *** |
| **December 2013** | 2019 | 56155 | 28071 | 50 | 69 | 73 | 81 | 0.0033 (0.0084) NS | -0.0151 (0.0270) NS | 0.0036 (0.0222) NS | 0.0126 (0.0081) NS | 0.1137 (0.0081) *** | **0.2236 (0.0080) ***** |
| **January 2014** | 2020 | 58381 | 28832 | 49,4 | 80 | 84 | 92 | 0.0142 (0.0083) NS | -0.0445 (0.0256) NS | -0.0176 (0.0216) NS | **0.0410 (0.0081) ***** | 0.1239 (0.0081) *** | 0.2412 (0.0081) *** |
| February 2014 | 2020 | 53207 | 26425 | 49,7 | 79 | 83 | 91 | -0.0024 (0.0086) NS | -0.0322 (0.0268) NS | -0.0102 (0.0229à NS | 0.0404 (0.0085) *** | 0.1111 (0.0085) *** | 0.2328 (0.0084) *** |
| March 2014 | 2020 | 57635 | 28335 | 49,2 | 78 | 82 | 90 | -0.0046 (0.0084) NS | 0.0047 (0.0259) NS | 0.0169 (0.0220) NS | 0.0400 (0.0082) *** | 0.1125 (0.0082) *** | 0.2467 (0.0082) *** |
| **Delta (Dec 13 - Jan 14)** | | 2226 | 761 | 0,6 | 11 | 11 | 11 | -0.0109 (0.0118) NS | 0.0294 (0.0372) NS | 0.0212 (0.0310) NS | -0.0284 (0.0115) * | -0.0102 (0.0115) NS | -0.0176 (0.0114) NS |
| | | | | | | | | | | | | | |
| October 2014 | 2020 | 60253 | 30071 | 49,9 | 71 | 75 | 83 | -0.0019 (0.0081) NS | -0.0208 (0.0254) NS | 0.0003 (0.0217) NS | 0.0338 (0.0079) *** | 0.1173 (0.0079) *** | 0.2434 (0.0079) *** |
| November 2014 | 2020 | 55970 | 27870 | 49,8 | 70 | 74 | 82 | -0.0059 (0.0084) NS | -0.0179 (0.0265) NS | -0.0152 (0.0224) NS | 0.0213 (0.0082) ** | 0.1113 (0.0082) *** | 0.2333 (0.0081) *** |
| **December 2014** | 2020 | 57347 | 28574 | 49,8 | 69 | 73 | 81 | 0.0132 (0.0084) NS | -0.0520 (0.0265) * | 0.0067 (0.0219) NS | -0.0015 (0.0080) NS | 0.0885 (0.0080) *** | **0.2167 (0.0080) ***** |
| **January 2015** | 2021 | 60053 | 29291 | 48,8 | 80 | 84 | 92 | 0.0001 (0.0081) NS | 0.0362 (0.0257) NS | 0.0235 (0.0213) NS | **0.0192 (0.0080) *** | 0.0938 (0.0080) *** | 0.2210 (0.0080) *** |
| February 2015 | 2021 | 54160 | 26768 | 49,4 | 79 | 83 | 91 | 0.0027 (0.0086) NS | -0.0200 (0.0267) NS | -0.0092 (0.0224) NS | 0.0372 (0.0085) *** | 0.1029 (0.0085) *** | 0.2213 (0.0084) *** |
| March 2015 | 2021 | 57892 | 28480 | 49,2 | 78 | 82 | 90 | -0.0003 (0.0084) NS | 0.0258 (0.0257) NS | -0.0230 (0.0218) NS | 0.0306 (0.0082) *** | 0.1051 (0.0082) *** | 0.2248 (0.0081) *** |
| **Delta (Dec 14 - Jan 15)** | | 2706 | 717 | 1 | 11 | 11 | 11 | 0.0131 (0.0117) NS | -0.0883 (0.0369) * | -0.0168 (0.0305) NS | -0.0207 (0.0114) NS | -0.0053 (0.0113) NS | -0.0043 (0.0113) NS |

Note : NS = Non significant ; * p < 0.05 ; ** p < 0.01 ; *** p < 0.001.

The columns marked "Gender gaps" used linear or logistic regressions to measure the size of the gender gap effect in the corresponding variables. The formulas were as follows (from left to right) : *lm(SES score ~ Gender) ; logit(Categories (Private; Public) ~ Gender) ; logit(Categories (Private and Public ; PE and HPE) ~ Gender) ;* and *lm(Math at Tx ~ Gender)*. Cells in **Bold** are the critical points compared in Table S7.

**Supplementary Table S10.** First stage LATE estimates: effect of day of birth around cutoff on age at math test

| Test | Sample | Conventional Estimate (SE) | Bias corrected estimate (SE) | N obs | N obs in band. | Band. Size |
|---|---|---|---|---|---|---|
| **T1** | Boys | 364.724 (0.162) *** | 364.673 (0.190) *** | 624311 | 174877 | 204 |
| | Girls | 364.799 (0.194) *** | 364.762 (0.237) *** | 607757 | 160660 | 192 |
| **T2** | Boys | 364.724 (0.162) *** | 364.673 (0.190) *** | 624311 | 174877 | 204 |
| | Girls | 364.799 (0.194) *** | 364.762 (0.237) *** | 607757 | 160660 | 192 |
| **T3** | Boys | 365.724 (0.162) *** | 365.673 (0.190) *** | 624311 | 174877 | 204 |
| | Girls | 365.799 (0.194) *** | 365.761 (0.236) *** | 607757 | 160660 | 192 |

Note: The table provides the Local Average Treatment Effect (LATE) of being born after January 1st, 2013, on the age at which children take the T1, T2 or T3 math tests, using a regression discontinuity design (RDD). The LATE estimates the impact of birthdate on the test-taking age for children at the age cutoff, analyzed separately for girls and boys. There is one row for each test and gender. RDD conventional estimator, its standard error, the bias-corrected estimator and its associated robust standard error are shown. For each estimate and its associated standard error, we recomputed p-values and used the standard convention: *** $p < 0.01$, ** $p < 0.05$, * $p < 0.1$. To get RDD estimates, separate polynomials are fitted on each side of the threshold. A triangular kernel is used. The polynomial order is 1, and the optimal bandwidths are derived under the MSERD procedure separately for each estimation. There are no control variables. The table also provides the initial number of observations in the sample, the number of observations in the estimation bandwidth and the size in days of the optimal bandwidths used for the estimation of the regression function.

**Supplementary Table S11.** Effect (LATE) of day of birth around cutoff on test scores

| Year | Test | Sample/ Estimate | Conventional estimate (SE) | Bias corrected Estimate (SE) | N obs | N obs in bandwidth | Bandwidth size |
|---|---|---|---|---|---|---|---|
| 2013 | T1 | Boys | 0.699 (0.009) *** | 0.698 (0.011) *** | 624311 | 237469 | 138 |
| | T1 | Girls | 0.624 (0.012) *** | 0.618 (0.014) *** | 607757 | 115405 | 69 |
| | T1 | Girls - Boys | -0.075 (0.015) *** | -0.079 (0.018) *** | | | |
| | T2 | Boys | 0.629 (0.010) *** | 0.630 (0.012) *** | 624311 | 209723 | 123 |
| | T2 | Girls | 0.537 (0.013) *** | 0.531 (0.015) *** | 607757 | 98862 | 60 |
| | T2 | Girls - Boys | -0.092 (0.016) *** | -0.099 (0.019) *** | | | |
| | T3 | Boys | 0.541 (0.010) *** | 0.538 (0.012) *** | 624311 | 211366 | 123 |
| | T3 | Girls | 0.533 (0.011) *** | 0.529 (0.013) *** | 607757 | 140564 | 85 |
| | T3 | Girls - Boys | -0.008 (0.015) | -0.009 (0.017) | | | |
| 2014 | T1 | Boys | 0.677 (0.010) *** | 0.675 (0.012) *** | 687979 | 187819 | 98 |
| | T1 | Girls | 0.632 (0.010) *** | 0.628 (0.011) *** | 672107 | 185797 | 99 |
| | T1 | Girls - Boys | -0.045 (0.014) *** | -0.047 (0.016) *** | | | |
| | T2 | Boys | 0.621 (0.011) *** | 0.624 (0.013) *** | 687979 | 149740 | 79 |
| | T2 | Girls | 0.585 (0.011) *** | 0.582 (0.012) *** | 672107 | 155798 | 83 |
| | T2 | Girls - Boys | -0.036 (0.016) ** | -0.042 (0.018) ** | | | |
| | T3 | Boys | 0.545 (0.011) *** | 0.544 (0.013) *** | 687979 | 178420 | 94 |
| | T3 | Girls | 0.520 (0.010) *** | 0.520 (0.012) *** | 672107 | 163145 | 88 |
| | T3 | Girls - Boys | -0.025 (0.015) | -0.024 (0.018) | | | |
| 2015 | T1 | Boys | 0.669 (0.011) *** | 0.667 (0.014) *** | 682852 | 149613 | 80 |
| | T1 | Girls | 0.634 (0.010) *** | 0.631 (0.011) *** | 668553 | 182437 | 99 |
| | T1 | Girls - Boys | -0.036 (0.015) ** | -0.037 (0.018) ** | | | |
| | T2 | Boys | 0.598 (0.011) *** | 0.597 (0.013) *** | 682852 | 159222 | 85 |
| | T2 | Girls | 0.570 (0.011) *** | 0.568 (0.013) *** | 668553 | 156330 | 85 |
| | T2 | Girls - Boys | -0.027 (0.015) | -0.029 (0.018) | | | |
| | T3 | Boys | 0.576 (0.010) *** | 0.576 (0.013) *** | 682852 | 179452 | 97 |
| | T3 | Girls | 0.559 (0.010) *** | 0.558 (0.012) *** | 668553 | 174666 | 95 |
| | T3 | Girls - Boys | -0.017 (0.014) | -0.017 (0.017) | | | |

Note: The table provides the Local Average Treatment Effect (LATE) of being born after January 1$^{st}$ of 2013, 2014 or 2015 (versus just before) on test scores when taking T1, T2 or T3 math test by RDD, separately for girls and boys. There are three rows per year and test: the first provides the estimated LATE for boys, the second the estimated LATE for girls, and the third the difference between these estimates. The RDD conventional estimator, its standard error, the bias-corrected estimator and its associated robust standard error are shown. The difference in estimates between boys and girls is provided for both estimators. For each estimate and its associated standard error, we recomputed p-values and used the standard convention: *** $p < 0.01$, ** $p < 0.05$, * $p < 0.1$. To get RDD estimates, separate polynomials are fitted on each side of the threshold. A triangular kernel is used. The polynomial order is 1, and the optimal bandwidths are derived under the MSERD procedure separately for each estimation. There are no control variables. The table also provides the initial number of observations in the sample, the number of observations in the whole estimation bandwidth, and the number of days on each side of the cutoff in the optimal bandwidths used for the estimation of the regression function.

**Supplementary Table S12:** Effect (LATE) of day of birth around cutoff on test scores with donut-hole

| Year | Test | Sample/ Estimate | Conventional estimate (SE) | Bias corrected Estimate (SE) | N obs | N obs in bandwidth | Bandwidth size |
|---|---|---|---|---|---|---|---|
| 2013 | T1 | Boys | 0.704 (0.010) *** | 0.703 (0.012) *** | 619191 | 228927 | 137 |
| | T1 | Girls | 0.633 (0.012) *** | 0.627 (0.014) *** | 602863 | 130610 | 81 |
| | T1 | Girls - Boys | -0.071 (0.016) *** | -0.076 (0.018) *** | | | |
| | T2 | Boys | 0.634 (0.010) *** | 0.636 (0.012) *** | 619191 | 213339 | 128 |
| | T2 | Girls | 0.555 (0.014) *** | 0.549 (0.016) *** | 602863 | 112318 | 70 |
| | T2 | Girls - Boys | -0.079 (0.017) *** | -0.087 (0.020) *** | | | |
| | T3 | Boys | 0.550 (0.010) *** | 0.550 (0.012) *** | 619191 | 221762 | 132 |
| | T3 | Girls | 0.540 (0.011) *** | 0.537 (0.013) *** | 602863 | 149178 | 93 |
| | T3 | Girls - Boys | -0.010 (0.015) | -0.013 (0.018) | | | |
| 2014 | T1 | Boys | 0.684 (0.010) *** | 0.684 (0.013) *** | 682221 | 201355 | 109 |
| | T1 | Girls | 0.634 (0.010) *** | 0.630 (0.012) *** | 666650 | 172366 | 96 |
| | T1 | Girls - Boys | -0.051 (0.015) *** | -0.054 (0.018) *** | | | |
| | T2 | Boys | 0.639 (0.014) *** | 0.645 (0.016) *** | 682221 | 120813 | 67 |
| | T2 | Girls | 0.591 (0.011) *** | 0.589 (0.013) *** | 666650 | 167134 | 92 |
| | T2 | Girls - Boys | -0.048 (0.018) *** | -0.056 (0.021) *** | | | |
| | T3 | Boys | 0.553 (0.012) *** | 0.553 (0.014) *** | 682221 | 170641 | 93 |
| | T3 | Girls | 0.527 (0.011) *** | 0.528 (0.013) *** | 666650 | 159316 | 89 |
| | T3 | Girls - Boys | -0.026 (0.016) | -0.026 (0.019) | | | |
| 2015 | T1 | Boys | 0.677 (0.012) *** | 0.676 (0.015) *** | 677592 | 147941 | 83 |
| | T1 | Girls | 0.634 (0.011) *** | 0.630 (0.012) *** | 663500 | 171392 | 97 |
| | T1 | Girls - Boys | -0.043 (0.016) *** | -0.046 (0.019) ** | | | |
| | T2 | Boys | 0.605 (0.012) *** | 0.606 (0.015) *** | 677592 | 153962 | 86 |
| | T2 | Girls | 0.577 (0.010) *** | 0.576 (0.012) *** | 663500 | 180878 | 101 |
| | T2 | Girls - Boys | -0.028 (0.016) | -0.031 (0.019) | | | |
| | T3 | Boys | 0.583 (0.011) *** | 0.584 (0.014) *** | 677592 | 170635 | 95 |
| | T3 | Girls | 0.563 (0.010) *** | 0.564 (0.013) *** | 663500 | 173457 | 97 |
| | T3 | Girls - Boys | -0.019 (0.015) | -0.021 (0.019) | | | |

Notes: The table provides the Local Average Treatment Effect (LATE) of being born just after January 1$^{st}$ of 2013, 2014 or 2015 (versus just before) on test scores when taking T1, T2 or T3 math test by RDD, separately for girls and boys. We use the so-called donut-hole approach: children born in a window of 3 days of each side of the cutoff data are excluded. There are three rows per year and test: the first provides the estimated LATE for boys, the second the estimated LATE for girls, and the third the difference between these estimates. Both the RDD conventional estimator, its standard error, the bias-corrected estimator and its associated robust standard error are shown. The difference in estimates between boys and girls is provided for both estimators. For each estimate and its associated standard error, we recomputed p-values and used the standard convention: *** $p < 0.01$, ** $p < 0.05$, * $p < 0.1$. To get RDD estimates, separate polynomials are fitted on each side of the threshold. A triangular kernel is used. The polynomial order is 1, and the optimal bandwidths are derived under the MSERD procedure separately for each estimation. There are no control variables. The table also provides the initial number of observations in the sample, the whole number of observations in the estimation bandwidth and the number of days on each side of the cutoff in the optimal bandwidths used for the estimation of the regression function.

**Supplementary Table S13.** Effect (LATE) of day of birth around cutoff on average school SES and the share of boys in class

| Covariate | Year | Sample/ estimate | Donut-hole approach | N obs | Conventional estimate (SE) | Bias corrected estimate (SE) | N obs | N obs in Bandwidth | Bandwidth size | p-value from manipulation test |
|---|---|---|---|---|---|---|---|---|---|---|
| SES school | 2013 | Boys | No | 624311 | -0.644 (0.191) *** | -0.655 (0.229) *** | 171442 | 171442 | 101 | 0.000 |
| | 2013 | Girls | No | 607757 | -0.439 (0.222) | -0.413 (0.267) ** | 128827 | 128827 | 77 | 0.000 |
| | | Girls - Boys | | | 0.205 (0.293) | 0.243 (0.352) | | | | |
| | 2014 | Boys | No | 687979 | 0.033 (0.213) | 0.102 (0.248) | 140099 | 140099 | 74 | 0.252 |
| | 2014 | Girls | No | 672107 | -0.374 (0.208) | -0.349 (0.251) | 144712 | 144712 | 77 | 0.469 |
| | | Girls - Boys | | | -0.408 (0.298) | -0.450 (0.353) | | | | |
| | 2015 | Boys | No | 682852 | -0.176 (0.189) | -0.190 (0.227) | 175895 | 175895 | 95 | 0.434 |
| | 2015 | Girls | No | 668553 | 0.132 (0.225) | 0.178 (0.268) | 124982 | 124982 | 68 | 0.213 |
| | | Girls - Boys | | | 0.308 (0.293) | 0.368 (0.351) | | | | |
| | 2013 | Boys | Yes | 619191 | -0.538 (0.185) ** | -0.565 (0.224) *** | 199580 | 199580 | 119 | 0.000 |
| | 2013 | Girls | Yes | 602863 | -0.222 (0.253) | -0.133 (0.299) | 112318 | 112318 | 71 | 0.000 |
| | | Girls - Boys | | | 0.316 (0.314) | 0.432 (0.374) | | | | |
| | 2014 | Boys | Yes | 682221 | 0.198 (0.242) | 0.293 (0.282) | 126562 | 126562 | 69 | 0.655 |
| | 2014 | Girls | Yes | 666650 | -0.357 (0.232) | -0.328 (0.285) | 133467 | 133467 | 75 | 0.816 |
| | | Girls - Boys | | | -0.555 (0.336) | -0.620 (0.401) | | | | |
| | 2015 | Boys | Yes | 677592 | -0.051 (0.195) | -0.034 (0.238) | 182152 | 182152 | 100 | 0.163 |
| | 2015 | Girls | Yes | 663500 | 0.185 (0.259) | 0.251 (0.313) | 111065 | 111065 | 63 | 0.961 |
| | | Girls - Boys | | | 0.236 (0.324) | 0.285 (0.393) | | | | |
| Share boys in class | 2013 | Boys | No | 624311 | 0.001 (0.001) | 0.001 (0.001) | 237469 | 237469 | 139 | 0.000 |
| | 2013 | Girls | No | 607757 | 0.001 (0.001) | 0.001 (0.001) | 202600 | 202600 | 122 | 0.000 |
| | | Girls - Boys | | | -0.001 (0.001) | -0.001 (0.001) | | | | |
| | 2014 | Boys | No | 687979 | -0.001 (0.001) | -0.001 (0.001) | 187819 | 187819 | 98 | 0.252 |
| | 2014 | Girls | No | 672107 | -0.002 (0.001) | -0.002 (0.001) ** | 252700 | 252700 | 136 | 0.469 |
| | | Girls - Boys | | | -0.001 (0.001) | -0.001 (0.001) | | | | |
| | 2015 | Boys | No | 682852 | -0.001 (0.001) | -0.001 (0.001) | 235209 | 235209 | 126 | 0.434 |
| | 2015 | Girls | No | 668553 | -0.001 (0.001) | -0.001 (0.001) | 182437 | 182437 | 99 | 0.213 |
| | | Girls - Boys | | | -0.000 (0.001) | -0.000 (0.001) | | | | |
| | 2013 | Boys | Yes | 619191 | 0.001 (0.001) | 0.001 (0.001) | 225414 | 225414 | 134 | 0.000 |
| | 2013 | Girls | Yes | 602863 | 0.001 (0.001) | 0.001 (0.001) | 169386 | 169386 | 105 | 0.000 |
| | | Girls - Boys | | | -0.001 (0.001) | -0.001 (0.002) | | | | |
| | 2014 | Boys | Yes | 682221 | -0.000 (0.001) | -0.000 (0.001) | 175964 | 175964 | 96 | 0.655 |
| | 2014 | Girls | Yes | 666650 | -0.002 (0.001) | -0.002 (0.001) ** | 239339 | 239339 | 132 | 0.816 |
| | | Girls - Boys | | | -0.002 (0.001) | -0.002 (0.002) | | | | |
| | 2015 | Boys | Yes | 677592 | -0.001 (0.001) | -0.001 (0.001) | 210949 | 210949 | 116 | 0.163 |
| | 2015 | Girls | Yes | 663500 | -0.001 (0.001) | -0.001 (0.001) | 169613 | 169613 | 96 | 0.961 |
| | | Girls - Boys | | | -0.000 (0.001) | -0.000 (0.002) | | | | |

Notes: The table provides the Local Average Treatment Effect (LATE) of being born just after January 1st of 2013, 2014 or 2015 (versus just before) on students' average school SES and share of boys in their class in Grade 1 by RDD, separately for girls and boys. We use either the standard approach or the so-called donut-hole approach. In the later case, children born in a window of 3 days of each side of the cutoff data are excluded. There are three

rows per variable and year: the first provides the estimated LATE for boys, the second the estimated LATE for girls, and the third the difference between these estimates. Both the RDD conventional estimator, its standard error, the bias-corrected estimator and its associated robust standard error are shown. The difference in estimates between boys and girls is provided for both estimators. For each estimate and its associated standard error, we recomputed p-values and used the standard convention: *** $p < 0.01$, ** $p < 0.05$, * $p < 0.1$. To get RDD estimates, separate polynomials are fitted on each side of the threshold. A triangular kernel is used. The polynomial order is 1, and the optimal bandwidths are derived under the MSERD procedure separately for each estimation. There are no control variables. The table also provides the initial number of observations in the sample, the whole number of observations in the estimation bandwidth, the size in days of number of days on each side of the cutoff in the optimal bandwidths used for the estimation of the regression function, and the p-value from a test of manipulation of the running variable at the cutoff. The test is based on the local polynomial density estimators proposed in Cattaneo, Jansson and Ma (2020). Results are obtained using the rddensity command in Stata using bias correction (results without bias correction are qualitatively similar).

**Supplementary Table S14.** Design of the matching experiments presented on Extended Data Figure 7 (n = 2,653,082 children).

| | **Matching at T1 only** | **Matching at T1 and T2** |
|---|---|---|
| School category (private, regular public, PE, HPE) | Exact | Exact |
| SES score, 50-150 | Same decile | Same decile |
| Age at T1, 69-80 (months) | +/- 4 months | +/- 4 months |
| 6 tests in math at T1, 0-100 | +/- 5 points | +/- 5 points |
| Language at T1, mean, 0-100 | +/- 5 points | +/- 5 points |
| Math at T2, mean, 0-100 | - | +/- 5 points |
| Language at T2, mean, 0-100 | - | +/- 5 points |
| Number of matched pairs in 2018 | 67,983 pairs | 9,142 pairs |
| Estimate of the gender effect at T3, Percent of success (SE) in 2018 | 5.156 (0.059) *** | 4.296 (0.128) *** |
| Number of matched pairs in 2019 | 94,279 pairs | 17,338 pairs |
| Estimate of the gender effect at T3, Percent of success (SE) in 2019 | 4.456 (0.049) *** | 3.257 (0.089) *** |
| Number of matched pairs in 2020 | 96,777 pairs | 19,448 pairs |
| Estimate of the gender effect at T3, Percent of success (SE) in 2020 | 4,209 (0.046) *** | 3.195 (0.079) *** |
| Number of matched pairs in 2021 | 106,878 pairs | 21,350 pairs |
| Estimate of the gender effect at T3, Percent of success (SE) in 2021 | 4.263 (0.046) *** | 3.099 (0.077) *** |

**Supplementary Table S15.** Sensitivity analysis of the imputed population in 2018 (n = 586,949).

| | Imputed population | Model 1 : Imputed vs. Original population (Non-imputed population) | p | Model 2: Imputed vs. Original population (Non-imputed population) from which missing values were removed | p |
|---|---|---|---|---|---|
| **N** | 586,949 | 586,949 | - | 465,934 | - |
| **Age at T1, mean (SD)** | 74.64 (3.84) | 74.64 (3.84) | 0.993 | 74.60 (3.79) | < 0.0001 |
| **Class size, mean (SD)** | 17.21 (5.85) | 17.21 (5.85) | 1.000 | 15.54 (5.46) | < 0.0001 |
| **Gender – Boys, n (%)** | 298,642 (50.9) | 298,642 (50.9) | 1.000 | 236,448 (50.7) | < 0.0001 |
| **Gender - Girls, n (%)** | 288,307 (49.1) | 288,307 (49.1) | - | 229,486 (49.3) | - |
| **SES score, mean (SD)** | 102.36 (17.79) | 102.35 (18.21) | 0.749 | 102.99 (18.21) | < 0.0001 |
| **Number of children in private schools (%)** | 63,304 (10.8) | 63,304 (11.4) | 1.000 | 56,664 (12.2) | < 0.0001 |
| **Number of children in regular public schools (%)** | 426,649 (72.7) | 426,649 (72.7) | - | 331,764 (71.2) | - |
| **Number of children in priority education PE (%)** | 58,416 (10.0) | 58,416 (10.0) | - | 47,467 (10.2) | - |
| **Number of children in high-priority education HPE (%)** | 38,580 (6.6) | 38,580 (6.6) | - | 30,039 (6.4) | - |
| **Math at T1, mean (SE)** | 72.77 (13.47) | 72.81 (13.56) | 0.067 | 73.29 (13.17) | < 0.0001 |
| **Math at T2, mean (SE)** | 76.43 (17.80) | 76.15 (18.25) | < 0.0001 | 77.73 (17.16) | < 0.0001 |
| **Math at T3, mean (SE)** | 68.75 (18.12) | 68.73 (18.17) | 0.548 | 69.79 (17.63) | < 0.0001 |
| **Problem solving at T1, mean (SE)** | 63.34 (30.12) | 63.47 (30.09) | 0.014 | 64.27 (29.81) | < 0.0001 |
| **Problem solving at T2, mean (SE)** | 68.60 (28.18) | 68.63 (28.21) | 0.535 | 70.92 (27.10) | < 0.0001 |
| **Problem solving at T3, mean (SE)** | 67.93 (27.27) | 67.95 (27.26) | 0.661 | 69.48 (26.57) | < 0.0001 |
| **Number line at T1, mean (SE)** | 51.01 (30.61) | 51.13 (30.61) | 0.026 | 51.81 (30.52) | < 0.0001 |
| **Number line at T2, mean (SE)** | 54.17 (24.74) | 54.20 (24.77) | 0.495 | 55.89 (24.21) | < 0.0001 |
| **Number line at T3, mean (SE)** | 47.11 (24.21) | 47.19 (24.21) | 0.062 | 47.94 (24.14) | < 0.0001 |
| **Language at T1, mean (SE)** | 72.69 (15.69) | 72.65 (15.80) | 0.124 | 73.51 (15.29) | < 0.0001 |
| **Language at T2, mean (SE)** | 64.62 (13.91) | 64.44 (14.61) | < 0.0001 | 60.18 (10.89) | < 0.0001 |
| **Language at T3, mean (SE)** | 71.29 (16.08) | 71.28 (16.36) | 0.777 | 61.82 (11.52) | < 0.0001 |

Note: Model 1 compared the scores of (1) the imputed population, corresponding to step 3 in the data management process; versus (2) the original population (non-imputed population) that underwent only exclusion of age outliers, gender outliers and selection on class size. Model 2 compared the scores of (1) the imputed population versus (2) the original non-imputed population from which missing values were removed. Units were in percent success for each variable, as gaussianization process was not applicable to the population with missing data in the model 1.

**Supplementary Table S16.** Progressive multilevel modelling of the mean Math score at T3 among children of typical age in first grade, 2018 cohort (n = 569,771).

| **Models** | **Model 1** Math T3 ~ 1 + (1 \| ID class) | | **Model 2** Math T3 ~ Math T1 + (1 + Math T1 \| ID class) | | **Model 3** Math T3 ~ Gender + (1 + Gender \| ID class) | | **Model 4** Math T3 ~ Math T1*Gender + (1 \| ID class) | | **Model 5** Math T3 ~ Math T1*Gender + (1 + Math T1 \| ID class) | | **Model 6** Math T3 ~ Math T1*Gender + (1 + Gender \| ID class) | |
|---|---|---|---|---|---|---|---|---|---|---|---|---|
| Fixed effects | Parameter estimate (Sd) | p | Parameter estimate (Sd) | p | Parameter estimate (Sd) | p | Parameter estimate (Sd) | p | Parameter estimate (Sd) | p | Parameter estimate (Sd) | p |
| Intercept | 0.0114 (0.0024) | < 0.0001 *** | 0.0107 (0.0019) | < 0.0001 *** | 0.0098 (0.0024) | < 0.0001 *** | 0.0041 (0.0019) | 0.0301 * | 0.0092 (0.0019) | < 0.0001 *** | 0.0041 (0.0019) | < 0.0001 *** |
| Math level at T1 | - | - | 0.6528 (0.0012) | < 0.0001 *** | - | - | 0.6424 (0.0010) | < 0.0001 *** | 0.6506 (0.0011) | < 0.0001 *** | 0.6423 (0.0010) | < *0.0001* *** |
| Gender (Boys) | - | - | - | - | 0.2542 (0.0025) | < 0.0001 *** | 0.2533 (0.0019) | < 0.0001 *** | 0.2530 (0.0019) | < 0.0001 *** | 0.2528 (0.0019) | < *0.0001* *** |
| Age at T1 (month) | - | - | - | - | - | - | - | - | - | - | - | - |
| SES score | - | - | - | - | - | - | - | - | - | - | - | - |
| First of class is a boy in math at T1 | - | - | - | - | - | - | - | - | - | - | - | - |
| Boys-Girls ratio per class | - | - | - | - | - | - | - | - | - | - | - | - |
| Class size | - | - | - | - | - | - | - | - | - | - | - | - |
| Heterogeneity of level in math at T1 | - | - | - | - | - | - | - | - | - | - | - | - |
| Gender * Age at T1 | - | - | - | - | - | - | - | - | - | - | - | - |
| Gender * Math level at T1 | - | - | - | - | - | - | 0.0527 (0.0019) | < 0.0001 *** | 0.0522 (0.0019) | < 0.0001 *** | 0.0544 (0.0010) | < 0.0001 *** |
| Gender * Language level at T1 | - | - | - | - | - | - | - | - | - | - | - | - |
| Gender * Heterogeneity of level at T1 | - | - | - | - | - | - | - | - | - | - | - | - |
| Gender * Class size | - | - | - | - | - | - | - | - | - | - | - | - |
| Gender * Boys-Girls ratio per class | - | - | - | - | - | - | - | - | - | - | - | - |
| Gender * First of class is a boy in math at T1 | - | - | - | - | - | - | - | - | - | - | - | - |
| Gender * SPI | - | - | - | - | - | - | - | - | - | - | - | - |
| Random effects | | | | | | | | | | | | |
| Between-class variance | | | | | | | | | | | | |
| Intercept variance | 0.1553 | | 0.1011 | | 0.1561 | | 0.1052 | | 0.1012 | | 0.1052 | |
| Gender variance | - | | - | | 0.0164 | | - | | - | | 0.0107 | |
| Math at T1 variance | - | | 0.0079 | | - | | - | | 0.0080 | | - | |
| Correlation Intercept Gender | - | | - | | 0.39 | | - | | 0.39 | | 0.14 | |
| Correlation Intercept Math T1 | - | | 0.43 | | - | | - | | - | | - | |
| Correlation Gender \| T1 Math | - | | - | | - | | - | | - | | - | |
| Within-class variance | 0.8351 | | 0.4910 | | 0.8143 | | 0.4799 | | 0.4740 | | 0.4772 | |
| Deviance (-2 log L) | 1564075.7 | | 1270071.2 | | 1552911.3 | | 1253150.3 | | 1251340.8 | | 1252985.6 | |

| Models | Model 7<br>Math T3 ~ Math T1*Gender + (1 + Math T1 + Gender \| ID class) | | Model 8<br>Math T3 ~ individual variables + (1 + Math T1 + Gender \| ID class) | | Model 9<br>Math T3 ~ individual variables + collective variables + (1 + Math T1 + Gender \| ID class) | | Model 10<br>Math T3 ~ individual variables + collective variables + interactions + (1 + Math T1 + Gender \| ID class) | |
|---|---|---|---|---|---|---|---|---|
| Fixed effects | Parameter estimate (Sd) | p | Parameter estimate (Sd) | p | Parameter estimate (Sd) | p | Parameter estimate (Sd) | p |
| Intercept | 0.0091 (0.0019) | < 0.0001 *** | 0.0106 (0.0018) | < 0.0001 *** | 0.0182 (0.0019) | < 0.0001 *** | 0.0107 (0.0019) | < 0.0001 *** |
| Math level at T1 | 0.6505 (0.0011) | < 0.0001 *** | 0.6455 (0.0012) | < 0.0001 *** | 0.6358 (0.0012) | < 0.0001 *** | 0.3810 (0.0013) | < 0.0001 *** |
| Gender (Boys) | 0.2523 (0.0019) | < 0.0001 *** | 0.2553 (0.0019) | < 0.0001 *** | 0.2532 (0.0020) | < 0.0001 *** | 0.3285 (0.0018) | < 0.0001 *** |
| Language level at T1 | - | - | - | - | - | - | 0.4078 (0.0013) | < 0.0001 *** |
| Age at T1 (month) | - | - | 0.0343 (0.0001) | < 0.0001 *** | 0.0361 (0.0002) | < 0.0001 *** | 0.0062 (0.0009) | < 0.0001 *** |
| SES score | - | - | - | - | 0.0883 (0.0019) | < 0.0001 *** | 0.0277 (0.0020) | < 0.0001 *** |
| First of class is a boy in math at T1 | - | - | - | - | 0.0099 (0.0018) | < 0.0001 *** | 0.0063 (0.0019) | 0.0008 ** |
| Boys-Girls ratio per class | - | - | - | - | -0.0023 (0.0017) | NS (0.192) | 0.0005 (0.0018) | NS (0.7742) |
| Class size | - | - | - | - | 0.0001 (0.0019) | NS (0.940) | 0.0095 (0.0020) | < 0.0001 *** |
| Heterogeneity of level in math at T1 | - | - | - | - | -0.0522 (0.0017) | < 0.0001 *** | -0.0305 (0.0018) | < 0.0001 *** |
| Gender * Age at T1 | - | - | - | - | - | - | -0.0094 (0.0019) | < 0.0001 *** |
| Gender * Math level at T1 | 0.0536 (0.0019) | < 0.0001 *** | - | - | - | - | 0.0644 (0.0024) | < 0.0001 *** |
| Gender * Language level at T1 | | | | | | | -0.0065 (0.0024) | 0.0075 ** |
| Gender * Heterogeneity of level at T1 | - | - | - | - | - | - | -0.0048 (0.0018) | 0.0081 ** |
| Gender * Class size | - | - | - | - | - | - | 0.0043 (0.0020) | 0.0275 * |
| Gender * Boys-Girls ratio per class | - | - | - | - | - | - | -0.0010 (0.0020) | NS (0.6124) |
| Gender * First of class is a boy in math at T1 | - | - | - | - | - | - | 0.0064 (0.0019) | 0.0006 ** |
| Gender * SES score | - | - | - | - | - | - | 0.0049 (0.0020) | 0.0146 * |
| Random effects | | | | | | | | |
| Between-class variance | | | | | | | | |
| Intercept variance | 0.1012 | | 0.1008 | | 0.0878 | | 0.1003 | |
| Gender variance | 0.0109 | | 0.0106 | | 0.0107 | | 0.0091 | |
| Math at T1 variance | 0.0079 | | 0.0079 | | 0.0080 | | 0.0046 | |
| Correlation Intercept \| Gender | 0.15 | | 0.11 | | 0.06 | | 0.13 | |
| Correlation Intercept \| Math T1 | 0.40 | | 0.39 | | 0.36 | | 0.32 | |
| Correlation Gender \| T1 Math | -0.27 | | -0.40 | | -0.39 | | -0.31 | |
| Within-class variance | 0.4807 | | 0.4711 | | 0.4713 | | 0.3982 | |
| Deviance (-2 log L) | 1251112.5 | | 1250806.8 | | 1247356.8 | | 1158166.8 | |

Note that the within-class variance was initially, in the empty model, of 0.8351 and in the complete final model of 0.3982, thus this full model explained 52,31% (= (0.8351 – 0.3982)/ (0. 8351)*100) of the within-class variance of math results at T3.

**Supplementary Table S17.** Multilevel regression model for Math at T1 among children of typical age at T1.

| | Math at T1 | | | | | | | |
|---|---|---|---|---|---|---|---|---|
| Cohort | **2018** | | **2019** | | **2020** | | **2021** | |
| N | 569,771 | | 665,632 | | 695,449 | | 722,230 | |
| Classes | 39,573 | | 46,671 | | 49,010 | | 49,701 | |
| Fixed effects | Estimate (sd) | p | Estimate (sd) | p | Estimate (sd) | p | Estimate (sd) | p |
| Intercept | -0.3449 (0.0033) | < 0.0001 | -0.3519 (0.0038) | < 0.0001 | -0.3403 (0.0037) | < 0.0001 | -0.3330 (0.0037) | < 0.0001 |
| Age at T1 (month) | 0.0551 (0.0003) | < 0.0001 | 0.0541 (0.0005) | < 0.0001 | 0.0522 (0.0004) | < 0.0001 | 0.0520 (0.0004) | < 0.0001 |
| Gender (Boys) | -0.0044 (0.0050) | NS (0.3832) | 0.0190 (0.0047) | 0.0001 | 0.0135 (0.0046) | 0.0031 | 0.0216 (0.0045) | < 0.0001 |
| SES score at T1 | 0.2666 (0.0024) | < 0.0001 | 0.2674 (0.0024) | < 0.0001 | 0.2905 (0.0024) | < 0.0001 | 0.2552 (0.0023) | < 0.0001 |
| Class size | -0.0779 (0.0025) | < 0.0001 | -0.0746 (0.0025) | < 0.0001 | -0.0767 (0.0024) | < 0.0001 | -0.0642 (0.0024) | < 0.0001 |
| Boys-Girls ratio per class | -0.0084 (0.0022) | 0.0001 | -0.0019 (0.0023) | NS (0.3906) | -0.0009 (0.0022) | NS (0.6736) | -0.0048 (0.0022) | 0.0313 |
| Gender * Age at T1 | 0.0010 (0.0007) | NS (0.1577) | 0.0018 (0.0006) | 0.0056 | 0.0022 (0.0006) | 0.0005 | 0.0008 (0.0006) | NS (0.1749) |
| Gender * SES score | 0.0257 (0.0026) | < 0.0001 | 0.0172 (0.0024) | < 0.0001 | 0.0171 (0.0024) | < 0.0001 | 0.0135 (0.0023) | < 0.0001 |
| Gender * Class size | -0.0088 (0.0026) | 0.0007 | -0.0014 (0.0024) | NS (0.5701) | -0.0047 (0.0024) | 0.0481 | -0.0003 (0.0023) | NS (0.9013) |
| Gender * Boys-Girls ratio per class | 0.0008 (0.0025) | NS (0.7573) | 0.0030 (0.0024) | NS (0.2094) | 0.0030 (0.0023) | NS (0.1861) | 0.0023 (0.0023) | NS (0.3125) |
| Between-class variance (Level 2) | | | | | | | | |
| Intercept variance | 0.1512 | | 0.1266 | | 0.1278 | | 0.1349 | |
| Within-class variance (Level 1) | 0.7530 | | 0.7629 | | 0.7540 | | 0.7603 | |
| Deviance (-2 log L) | 1508033.3 | | 1766520.4 | | 1838940 | | 1916132.3 | |

Note: The formula implemented was as follow: *Math at T1 ~ Age at T1 + Gender + SES score at T1 + Class size + Boys-Girls ratio per class + Gender * Age at T1 + Gender * SES score + Gender * Class size + Gender * Boys-Girls ratio per class + (1 | class)*

**Supplementary Table S18.** Multilevel regression model for Math at T2 among children of typical age at T1.

| | Math at T2 | | | | | | | |
|---|---|---|---|---|---|---|---|---|
| Cohort | **2018** | | **2019** | | **2020** | | **2021** | |
| N | 569,771 | | 665,632 | | 695,449 | | 722,230 | |
| N group (classes) | 39,573 | | 46,671 | | 49,010 | | 49,701 | |
| Fixed effects | Estimate (sd) | p | Estimate (sd) | p | Estimate (sd) | p | Estimate (sd) | p |
| Intercept | -0.0155 (0.0026) | < 0.0001 | -0.1280 (0.0028) | < 0.0001 | -0.1192 (0.0027) | < 0.0001 | -0.0993 (0.0028) | < 0.0001 |
| Language level at T1 | 0.3937 (0.0013) | < 0.0001 | 0.3807 (0.0016) | < 0.0001 | 0.3726 (0.0016) | < 0.0001 | 0.3782 (0.0017) | < 0.0001 |
| Math level at T1 | 0.3838 (0.0014) | < 0.0001 | 0.4090 (0.0017) | < 0.0001 | 0.4216 (0.0017) | < 0.0001 | 0.3882 (0.0017) | < 0.0001 |
| Gender (Boys) | 0.1530 (0.0038) | < 0.0001 | 0.1792 (0.0034) | < 0.0001 | 0.1625 (0.0033) | < 0.0001 | 0.1480 (0.0034) | < 0.0001 |
| Age at T1 (month) | 0.0037 (0.0003) | < 0.0001 | 0.0072 (0.0003) | < 0.0001 | 0.0066 (0.0003) | < 0.0001 | 0.0049 (0.0003) | < 0.0001 |
| First of class is a boy in math at T1 | 0.0062 (0.0019) | 0.0012 | 0.0033 (0.0019) | 0.0740 | 0.0023 (0.0018) | 0.1966 | 0.0045 (0.0018) | 0.0147 |
| Boys-Girls ratio per class | -0.0033 (0.0018) | 0.0662 | -0.0064 (0.0018) | 0.0004 | -0.0049 (0.0017) | 0.0045 | -0.0028 (0.0018) | 0.1236 |
| Class size | -0.0014 (0.0020) | 0.4908 | 0.0015 (0.0020) | 0.4386 | 0.0045 (0.0019) | 0.0173 | 0.0038 (0.0019) | 0.0443 |
| SES score | -0.0028 (0.0020) | 0.1602 | -0.0196 (0.0020) | < 0.0001 | -0.0377 (0.0019) | < 0.0001 | -0.0220 (0.0019) | < 0.0001 |
| Heterogeneity of level in math at T1 | -0.0346 (0.0018) | < 0.0001 | -0.0098 (0.0018) | < 0.0001 | -0.0093 (0.0017) | < 0.0001 | -0.0070 (0.0017) | 0.0001 |
| Gender * Language level at T1 | -0.0179 (0.0025) | < 0.0001 | -0.0214 (0.0022) | < 0.0001 | -0.0252 (0.0022) | < 0.0001 | -0.0251 (0.0022) | < 0.0001 |
| Gender * Math level at T1 | 0.0211 (0.0025) | < 0.0001 | 0.0381 (0.0022) | < 0.0001 | 0.0346 (0.0022) | < 0.0001 | 0.0367 (0.0022) | < 0.0001 |
| Gender * Age at T1 | -0.0006 (0.0005) | 0.2464 | -0.0010 (0.0005) | 0.0247 | 0.0004 (0.0005) | 0.3632 | 0.0006 (0.0005) | 0.1740 |
| Gender* First of class is a boy in math at T1 | -0.0053 (0.0018) | 0.0041 | -0.0027 (0.0016) | 0.0980 | -0.0024 (0.0016) | 0.1351 | -0.0034 (0.0016) | 0.0384 |
| Gender * Boys-Girls ratio per class | -0.0001 (0.0020) | 0.9491 | 0.0008 (0.0017) | 0.6328 | -0.0015 (0.0017) | 0.3731 | -0.0024 (0.0017) | 0.1708 |
| Gender * Class size | -0.0021 (0.0020) | 0.2948 | -0.0014 (0.0017) | 0.4177 | 0.0001 (0.0017) | 0.9494 | 0.0012 (0.0017) | 0.4684 |
| Gender * SES score | 0.0148 (0.0020) | < 0.0001 | 0.0089 (0.0018) | < 0.0001 | 0.0135 (0.0018) | < 0.0001 | 0.0055 (0.0018) | 0.0018 |
| Gender * Heterogeneity in math at T1 | -0.0024 (0.0018) | 0.1918 | -0.0029 (0.0016) | 0.0651 | -0.0030 (0.0016) | 0.0549 | -0.0010 (0.0016) | 0.5493 |
| Random effects | | | | | | | | |
| Between-class variance Intercept | 0.1026 | | 0.0910 | | 0.0833 | | 0.0956 | |
| Class level \| Gender | 0.0041 | | 0.0032 | | 0.0036 | | 0.0053 | |
| Class level \| T1 Math | 0.0068 | | 0.0034 | | 0.0041 | | 0.0094 | |
| Correlation Class level intercept \| Gender | -0.02 | | -0.23 | | -0.20 | | -0.14 | |
| Correlation Class level intercept \| T1 Math | 0.18 | | -0.01 | | 0.01 | | 0.25 | |
| Correlation Gender \| T1 Math | 0.15 | | -0.05 | | -0.13 | | 0.04 | |
| Within-class variance (residuals) | 0.4121 | | 0.3751 | | 0.3759 | | 0.4012 | |
| Deviance (-2 log L) | 1177958.9 | | 1308348.5 | | 1366670.4 | | 1474527.4 | |

Note: The formula implemented was as follow: *Math at T2 ~ Age at T1 + Gender + Math level at T1 + language level at T1 + First of class being a boy in math + SES score at T1 + Class size + Boys-Girls ratio per class + Heterogeneity of level in math in the class + 8 interactions between each variable and Gender + (1 + Gender + Math level at T1 | class)*

**Supplementary Table S19.** Bayes Factors for the null hypothesis of Math at T1 and of Math at T3.

| | **Model with Math T1** | | | | **Model with Math T3** | | | |
|---|---|---|---|---|---|---|---|---|
| | Multilevel regression model | | Bayes Factor | | Linear regression model | | Bayes Factor | |
| N | 569,771 | p | 569,771 | Interpretation $H_0$: Null Hypothesis $H_1$: Alternative Hypothesis | 569,771 | p | 569,771 | Interpretation $H_0$: Null Hypothesis $H_1$: Alternative Hypothesis |
| Classes | 39,573 | | 39,573 | | 39,573 | | 39,573 | |
| Language individual level at T1 | - | - | - | - | 0.3605 (0.0013) | < 0.0001 | $5.0908 \times 10^{15428}$ ± 2.24% | Extreme evidence for $H_1$ |
| Math individual level at T1 | - | - | - | - | 0.3646 (0.0013) | < 0.0001 | $5.9850 \times 10^{16134}$ ± 2.37% | Extreme evidence for $H_1$ |
| Gender (Boys) | **-0.0044 (0.0050)** | **NS (0.3832)** | **0.0040 ± 1.04%** | **Extreme evidence for $H_0$** | 0.3009 (0.0020) | < 0.0001 | $6.4527 \times 10^{5258}$ ± 2.05% | Extreme evidence for $H_1$ |
| SES score at T1 | 0.2666 (0.0024) | < 0.0001 | $8.1240 \times 10^{7058}$ ± 1.69% | Extreme evidence for $H_1$ | 0.0516 (0.0011) | < 0.0001 | $2.3405 \times 10^{507}$ ± 2.83% | Extreme evidence for $H_1$ |
| Age at T1 (month) | 0.0551 (0.0003) | < 0.0001 | $1.6819 \times 10^{4993}$ ± 2.56% | Extreme evidence for $H_1$ | 0.0234 (0.0011) | < 0.0001 | $7.0375 \times 10^{104}$ ± 6.21% | Extreme evidence for $H_1$ |
| Heterogeneity of level in math at T1 | - | - | - | - | -0.0374 (0.0010) | < 0.0001 | $1.3040 \times 10^{334}$ ± 2.84% | Extreme evidence for $H_1$ |
| Boys-Girls ratio per class | -0.0084 (0.0022) | 0.0001 | $1.3475 \times 10^{7}$ ± 1.74% | Extreme evidence for $H_1$ | 0.0012 (0.0010) | NS 0.21930 | 0.0102 ± 6.03% | Very strong evidence for $H_0$ |
| First of class is a boy in math at T1 | - | - | - | - | 0.0091 (0.0020) | < 0.0001 | 227.5226 ± 2.83% | Extreme evidence for $H_1$ |
| Class size | -0.0779 (0.0025) | < 0.0001 | $9.9464 \times 10^{499}$ ± 1.49% | Extreme evidence for $H_1$ | -0.0028 (0.0010) | 0.00563 | 0.2436 ± 4.47% | Moderate evidence for $H_0$ |
| Gender * Language individual level at T1 | - | - | - | - | -0.0045 (0.0026) | NS 0.08303 | 0.0245 ± 4.67% | Very strong evidence for $H_0$ |
| Gender * Math individual level at T1 | - | - | - | - | 0.0674 (0.0026) | < 0.0001 | $4.2791 \times 10^{144}$ ± 2.83% | Extreme evidence for $H_1$ |
| Gender * SES score at T1 | 0.0257 (0.0026) | < 0.0001 | $8.7220 \times 10^{10}$ ± 1.33% | Extreme evidence for $H_1$ | 0.0034 (0.0021) | NS 0.10861 | 0.0181 ± 2.85% | Very strong evidence for $H_0$ |
| Gender * Age at T1 | 0.0010 (0.0007) | NS (0.1577) | 0.0118 ± 1.34% | Very strong evidence for $H_0$ | -0.0120 (0.0021) | < 0.0001 | $0.7098 \times 10^{5}$ ± 3.96% | Extreme evidence for $H_1$ |
| Gender * Heterogeneity of level at T1 | - | - | - | - | -0.0022 (0.0019) | NS 0.24260 | 0.0104 ± 5.24% | Very strong evidence for $H_0$ |
| Gender * Boys-Girls ratio per class | 0.0008 (0.0025) | NS (0.7573) | 0.4656 ± 1.79% | Anectotal evidence for $H_0$ | -0.0080 (0.0020) | < 0.0001 | 15.7365 ± 2.84% | Strong evidence for $H_1$ |
| Gender * First of class is a boy in math at T1 | - | - | - | - | 0.0327 (0.0039) | < 0.0001 | $4.4989 \times 10^{12}$ ± 2.81% | Extreme evidence for $H_1$ |
| Gender * Class size | -0.0088 (0.0026) | 0.0007 | 0.1702 ± 5.09% | Moderate evidence for $H_0$ | 0.0053 (0.0021) | 0.00932 | 0.1438 ± 2.84% | Moderate evidence for $H_0$ |

Note that the Bayes Factor denominator used was the full following model for Math at T1 : "Math at T1 ~ Age + Gender + SES score + Class size + Boys-Girls ratio per class + Gender * Age + Gender * SES score + Gender * Class Size + Gender * Boys-Girls ratio per class" and the Bayes Factor denominator used was the full following model for Math at T3 : "Math at T3 ~ Age + Gender + Math at T1 + Language at T1 + First of class in Math at T1 + SES score + Class size + Boys-Girls ratio per class + Heterogeneity of level in Math at T1 + Gender * Age + Gender * Math at T1 + Gender * Language at T1 + Gender * First of class in Math at T1 + Gender * SES score + Gender * Class Size + Gender * Boys-Girls ratio per class + Gender * Heterogeneity of level in Math at T1"

## Supplementary Figures

**SI Figure S1. Distribution of birth dates for children born around January 1st and taking math tests, for all cohorts from 2018 to 2021** (n = 2,653,082 children).

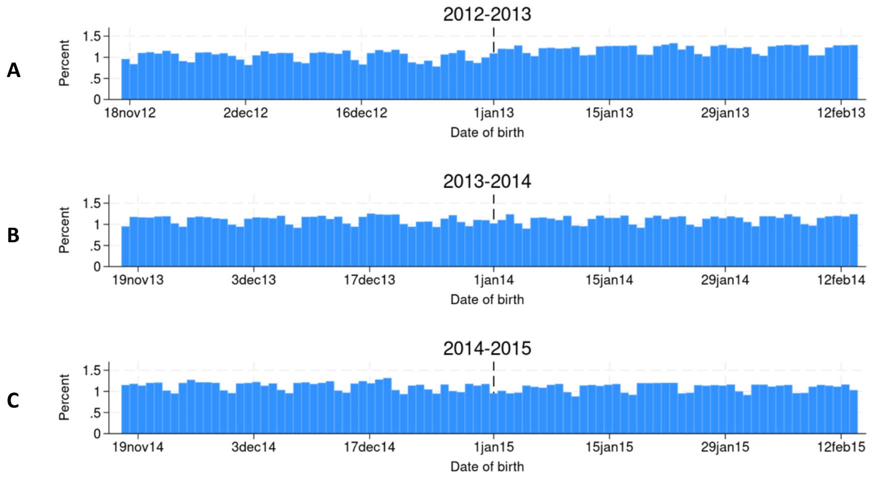


Each cohort was merged with the next cohort to obtain continuity in birth dates around January 1st. Therefore, **(A)** shows the birth dates continuity of children who entered first grade in 2018 (born until December 31st 2012) and in 2019 (born from January 1st 2013); **(B)** Birth dates of children who entered first grade in 2019 and in 2020; **(C)** Birth dates of children who entered first grade in 2020 and 2021. The distribution of birth dates revealed some very local fluctuations: there were fewer births during weekends, and, to some extent, on January 1st. This is probably because some births (e.g. caesarean) were planned on weekdays or outside holidays.

**SI Figure S2. Age at T1 and math scores at T1 as a function of day of birth around 1st January 2013** (n = 2,653,082 children).

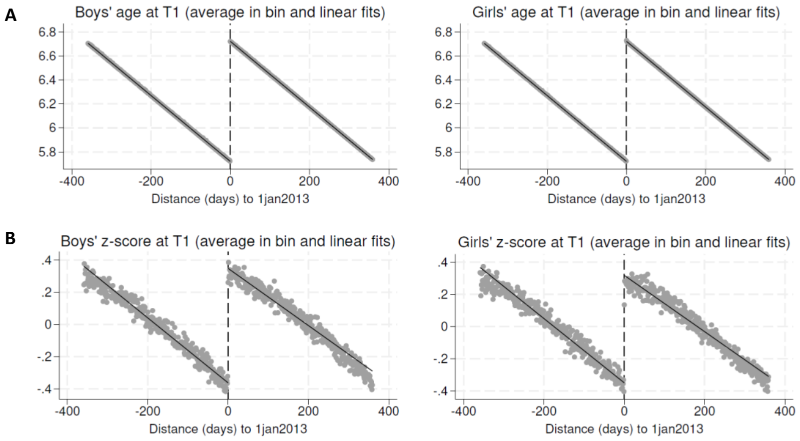


This figure showed discontinuities in age at T1 and in T1 math scores as a function of day of birth at the first available cut-off (1st January 2013) for girls and boys separately, picturing a broad 2-year window on birth date. **(A)** Age when taking the T1 test as a function of day of birth, making it clear that (i) obviously, the later they are born, the younger children are when taking T1, but (ii) this is no longer true when they cross the January 1st institutional threshold on school entry, as they become one year older at T1. This effect of the date of birth on age at T1 is very similar for girls and boys, as showed in a Regression Discontinuity Design (see SI). **(B)** T1 math scores as a function of day of birth, indicating that (i) the later they were born, the worse children performed at T1, (ii) this was no longer true when they crossed the January 1st threshold: as they were one year older, their performance increased by about 0.7 SD (see SI). Crucially, the effect of the date of birth on math performance at T1 was comparable for girls and boys.

## Supplementary Discussion

### Supplementary discussion on the possible specificities of France

We were able to collect data from one country (France), yet in a fully exhaustive manner and across 4 years. To what extent are the present findings generalizable to other countries? It seems likely that the observed triggering of the math gender gap by schooling in 1st grade will generalize, given its universal reproducibility across ages, SES, initial level in math, types of classes, and 4 consecutive years. Nevertheless, it cannot be excluded that our results are affected at least in part by peculiarities of France's education system. Here we document a few specificities that may or may not be relevant.

First, France implements a strong distinction between "maternelle" (preschool) and "école primaire" (primary school). Until very recently, preschool was only considered as a preparatory stage based essentially on games and collective activities, where formal teaching was explicitly banned. Thus, formal teaching started quite suddenly with entry into first grade, both for reading and for math, and this may explain the suddenness with which the math gender gap appears in our data. As explained in the main text, the situation changed recently, as ministry instructions now enforce more formal preparation to reading and math already in preschool. This change may explain why in the most recent cohorts of 2019, 2020 and 2021, a small but significant math gender gap is already perceptible at T1. A prediction of this view, which could be tested in the future, is that countries that introduce teaching of a more formal nature at an earlier age may evidence earlier and perhaps stronger gender gaps in math.

A second specificity of France is the highly competitive nature of the domain of mathematics. France has a long history of successfully training high-level mathematicians, for instance ranking 2nd in the world for Fields medalists. Some mathematicians achieve high social rank and fame. At school, for decades, mathematical grades were used as a selection tool for children to enter the most selective and difficult classes. The curriculum was often viewed, by parents and educators alike, as a one-dimensional scale where mathematics was placed on top, while other disciplines such as humanities or, to an even greater extent, artistic or manual training, were considered less prestigious. This bias is now receding, as French teenagers now have the possibility of choosing their own mix of majors and minors. Furthermore, it is unclear whether it ever affected 1st and 2nd grade, but it cannot be excluded that it contributed to the present data.
