# Supplementary material for: Rapid emergence of a maths gender gap in first grade
Source: Nature. Author manuscript; Available in PMC 2025 Dec 11. (PMC7618463; doi:10.1038/s41586-025-09126-4)
Supplement: Supplementary Materials [file EMS210438-supplement-Supplementary_Materials.pdf]

nature portfolio

Corresponding author(s): Pauline MARTINOT and Stanislas DEHAENE

Last updated by author(s): Apr 11, 2025

Reporting Summary

Nature Portfolio wishes to improve the reproducibility of the work that we publish. This form provides structure for consistency and transparency in reporting. For further information on Nature Portfolio policies, see our Editorial Policies and the Editorial Policy Checklist.

Statistics

For all statistical analyses, confirm that the following items are present in the figure legend, table legend, main text, or Methods section.

n/a	Confirmed	
☐	☒	The exact sample size (*n*) for each experimental group/condition, given as a discrete number and unit of measurement
☒	☐	A statement on whether measurements were taken from distinct samples or whether the same sample was measured repeatedly
☐	☒	The statistical test(s) used AND whether they are one- or two-sided<br>*Only common tests should be described solely by name; describe more complex techniques in the Methods section.*
☐	☒	A description of all covariates tested
☐	☒	A description of any assumptions or corrections, such as tests of normality and adjustment for multiple comparisons
☐	☒	A full description of the statistical parameters including central tendency (e.g. means) or other basic estimates (e.g. regression coefficient) AND variation (e.g. standard deviation) or associated estimates of uncertainty (e.g. confidence intervals)
☐	☒	For null hypothesis testing, the test statistic (e.g. *F*, *t*, *r*) with confidence intervals, effect sizes, degrees of freedom and *P* value noted<br>*Give P values as exact values whenever suitable.*
☐	☒	For Bayesian analysis, information on the choice of priors and Markov chain Monte Carlo settings
☐	☒	For hierarchical and complex designs, identification of the appropriate level for tests and full reporting of outcomes
☐	☒	Estimates of effect sizes (e.g. Cohen's *d*, Pearson's *r*), indicating how they were calculated

Our web collection on statistics for biologists contains articles on many of the points above.

Software and code

Policy information about availability of computer code

Data collection	Data were collected on a national scale and centralized by the DEPP (National department of statistics and research at the National Education Ministry) team at the Ministry of Education in France. Their ethical committee ensured that all parents were fully informed and gave their consent for the analysis of their children's data.<br><br>A formal agreement was established between our research lab and the DEPP structure, enabling local use and access to the data. The R software, version 4.3.2, was utilized, and it can be found at the following link: https://cran.r-project.org/bin/windows/base/.<br><br>All scripts, encompassing both data management and analysis, are openly accessible on the following repository: https://github.com/PauMdlm/Gendergaps.<br><br>As outlined in both our article and the methodology section, we applied a specific data management and analysis approach to all children in the academic years 2018-2019, 2019 -2020, 2020-2021, and 2021-2022.<br><br>Given that the data adhered to the GDPR European law on data protection, extraction of data from the DEPP structure was not permitted. However, we shared a simulated dataset to gain an initial understanding of how the data were organized and how the data management was structured.
Data analysis	For all the data management and data analysis, we used the R software in the latest version 4.3.2.<br>All of our scripts - both for data management and data analysis - were presented in open access on the following repository : https://github.com/PauMdlm/Gendergaps.

The scripts are ordered by numbers, and we added a README document to precise the duration of script running as well as how to obtain the access to the data at the DEPP structure based in Paris, France.

R Packages used included : rstatix, FactoMineR, dplyr, tidyverse, broom, ggplot2, jtools, LambertW, reshape2, lmerTest, knitr, rmarkdown, MatchIt, remotes, rcpp, glmertree, BayesFactor, mice, tableone, cohens_d; all of them using R software 4.3.2 version. In addition, RDD was performed with Stata (version 18, 2023) using the following packages: rdrobust

For manuscripts utilizing custom algorithms or software that are central to the research but not yet described in published literature, software must be made available to editors and reviewers. We strongly encourage code deposition in a community repository (e.g. GitHub). See the Nature Portfolio guidelines for submitting code & software for further information.

Data

Policy information about availability of data

All manuscripts must include a data availability statement. This statement should provide the following information, where applicable:

- Accession codes, unique identifiers, or web links for publicly available datasets
- A description of any restrictions on data availability
- For clinical datasets or third party data, please ensure that the statement adheres to our policy

Original data cannot be openly accessed; they are housed in a secure repository made available to researchers who establish an agreement with the DEPP Statistical Department of the Ministry of Education, located in central Paris, France. This agreement took nine months to be finalized, granting us access to the data.
Local computers are provided, with secure access to both the R software and the internet, enabling us to manage and retrieve the data.

Furthermore, the DEPP department is currently working on making the data accessible remotely online, with the anticipated launch scheduled for 2025.

Research involving human participants, their data, or biological material

Policy information about studies with human participants or human data. See also policy information about sex, gender (identity/presentation), and sexual orientation and race, ethnicity and racism.

Reporting on sex and gender	The primary focus of our study centered around the gender gap.<br><br>Within our research, gender was recorded in a binary manner, specifically as either male or female, and reported by the teacher.<br><br>Consistent with recommendations from the literature, we consistently employed the term "gender" rather than "sex" throughout this manuscript, emphasizing that the gender classification was provided by an external source.
Reporting on race, ethnicity, or other socially relevant groupings	Reporting on either race or ethnicity are not allowed in the research fied in France, therefore we did not have any reports about these domains.<br><br>We used three types of socioeconomical data :<br><br>1) firstly, we used the "socioeconomical status index" created by the statistical department research team in France and described as followed in our material and methods, in the subsection "School socioeconomical status (SES)".<br>This score reflected the environment socioeconomical status surrounding children. Computed by DEPP, it was a combination of the following data: parents 'diploma level, material conditions level, family composition, cultural capital, cultural ambition, parental implication levels and cultural practices. All these data were registered in 6th grade only. Thus, a retrospective projection of every child's socioeconomical characteristics was implemented, by post-hoc attribution of the SES score to the primary school that a given 6th grader had attended. Finally, the school SES score was computed as the mean of all SES scores of children who attended the same primary school (see Rocher et. al.). Ultimately, SES was a numerical variable, defined by the DEPP, going from ~ 50 to ~ 150 and representing school socioeconomic status, 50 being the lowest, and 150 the most advantageous.<br><br>2) Secondly, we analyzed the type of school children went to, described in the subsection "School category" in material and methods.<br>This variable was defined by the DEPP and the ministry of education using a combination of school status (private or public) and of four additional characteristics: the proportion of disadvantaged socio-professional categories in the geographic area surrounding the school; the proportion of students benefitting from social aid and scholarships in the living area surrounding the school; the proportion of pupils living in a sensitive urban area within the school; and the proportion of pupils attending the school who repeated a school year before their sixth grade.<br><br>3) Thirdly, we analyzed the profession of each parents, declared by children at the entry of grade 6, and linked to their profile and results at grade 1.<br><br>Our study's exhaustive data allowed us to consider all children, especially the lower SES subpopulations - which are frequently withdrawn from studies because of their higher rate of missing data.
Population characteristics	The population of our study is about age 6, with children being in advanced (Age 5) or late (Age 7) when starting their first grade, in France. Both gender were included in our study, all socioeconomical levels were included and the exhaustive data were analyzed (no sample) for the following school years : 2018-2019, 2019-2020, 2020-2021, 2021-2022.
Recruitment	Every first and second grade's teachers of France had to make their students go through the national evaluations for 2 days

Recruitment

all at the same time (end of september Year 1, end of january and end of september Year 2). For every child of their class, teachers reported their results in a secured file, that the district gathered, anonymized and send at the national level (to the DEPP department). We accessed four consecutive longitudinal French national assessment cohorts, targeting all French children entering 1st grade respectively in 2018, 2019, 2020 and in 2021 and all 2nd graders entering 2nd grade in 2019, 2020, 2021 and 2022.

More precisely as mentionned in the material and methods section, 46 tests were administered by teachers over a 12-months period. Assessments were implemented at three specific times: beginning of 1st grade (between the 3rd and 4th week of September), hereafter called T1; middle of 1st grade (between the 3rd and 4th week of January; T2) and beginning of 2nd grade (between the 3rd and 4th week of September; T3). Each test aimed to assess specific skills in oral language, reading, mathematic and problem solving.

In the days following testing, teachers and schools were responsible for entering every individual response in a dedicated computerized system, then the data were copied and anonymized at the regional level and sent to the national level where they were stored following the European General Data Protection Regulation (GDPR). Parents were informed about these national assessments. All children were tested, but parents could refuse the use of their children's data for further statistical purposes. As the whole population of children in France per year was included, no subgroups nor samples were selected, therefore, "selection biases" are not applicable.

Ethics oversight

The organization that approved the ethical statement and study protocol was the DEPP at the Ministry of Education.

Inclusion and ethic statement
Every year – 2018, 2019, 2020 and 2021-2022, all children of first and second grades were tested at school, within their classroom, in France. Once data were anonymized, they were sent and stored at the national statistical institution of the ministry of education (DEPP) on approved European General Data Protection Regulation (GDPR) servers. The National Education Data Ethics Committee, composed of qualified members, ensures compliance with legal framework regarding both educational data protection and use. Parents were informed about these national assessments and data second use in research. All children were tested, but parents could - at any moment - refuse the use of their children's data for further statistical purposes.

Note that full information on the approval of the study protocol must also be provided in the manuscript.

Field-specific reporting

Please select the one below that is the best fit for your research. If you are not sure, read the appropriate sections before making your selection.

☐ Life sciences ☒ Behavioural & social sciences ☐ Ecological, evolutionary & environmental sciences

For a reference copy of the document with all sections, see nature.com/documents/nr-reporting-summary-flat.pdf

Behavioural & social sciences study design

All studies must disclose on these points even when the disclosure is negative.

Study description

This study is a quantitative longitudinal study based on observational data, using multi level models, growth models, Bayesian models, Regression discontinuity design models, Matching and causal inference methods, on observational data, to estimate the associations between school exposure and gender gaps in math in first and second grade.

Research sample

As we included the whole population of France that started first and second grade from 2018 to 2022, we did not present with any sample, but rather an exhaustive population. Age was of 6.5 years in average and both gender were quasi equally represented (slightly more boys than girls, every year).

Sampling strategy

As the whole population of a children's age was included, no sample nor sub-cohorts were defined, neither were any sampling strategy. The whole population data was available every year from 2018 to 2022.

Data collection

The researcher was blind when data was collected. Teachers and children were provided with national evaluation notebook and a pen. Once the testing session was over, teachers had to enter manually the results for each child, in a specific file on a computer. This file was then sent to the education district, where it was anonymized and then sent to the national district of education. There, the DEPP built a secured access to these exhaustive collected data.

Timing

Start: September 2018
Stop : September 2022
There was no interruption, even during the Covid-19 school year, teachers and administrators managed to plan the national evaluations and gather results for the whole population of first and second graders in France.

Data exclusions

Among the four cohorts (which respectively comprised 610,905, 711,452, 743,734, and 804,989 children for a total of 2,871,080 children), we excluded the following outliers : age outliers (A total of 169, 310, 261 and 446 children had aberrant birthdates respectively in 2018, 2019, 2020 and 2021) ; the missing values on three entire sessions (A total of 75, 101, 128 and 1222 children were excluded because all sessions were missing, respectively in 2018, 2019, 2020 and 2021); missing values on gender (A total of 60, 41, 135 and 0 children were removed respectively in 2018, 2019, 2020 and 2021)

Non-participation

As we included the whole population of France that started first and second grade from 2018 to 2022 we presented with an

Non-participation	exhaustive population, then excluded the above mentioned outliers which represented about 0.0003 % age outliers and 0.0001% missing all the sessions.
Randomization	N/A. The study population was exhaustive. Participants were not allocated to subgroups nor needed any randomization.

Reporting for specific materials, systems and methods

We require information from authors about some types of materials, experimental systems and methods used in many studies. Here, indicate whether each material, system or method listed is relevant to your study. If you are not sure if a list item applies to your research, read the appropriate section before selecting a response.

Materials & experimental systems

n/a	Involved in the study
☒	☐ Antibodies
☒	☐ Eukaryotic cell lines
☒	☐ Palaeontology and archaeology
☒	☐ Animals and other organisms
☒	☐ Clinical data
☒	☐ Dual use research of concern
☒	☐ Plants

Methods

n/a	Involved in the study
☒	☐ ChIP-seq
☒	☐ Flow cytometry
☒	☐ MRI-based neuroimaging

Plants

Seed stocks	n/a
Novel plant genotypes	n/a
Authentication	n/a
